# Structural analysis of lanthanide–DOTAM coordination complexes and use of machine learning to rationally design materials with molecular recognition for selective rare earth recovery

**DOI:** 10.1039/d6sc03531k

**Published:** 2026-07-15

**Authors:** Nicole M. Shapiro, Harindu Rajapaksha, Xiaohui Qu, Sara E. Mason, David M. Cwiertny, Tori Z. Forbes

**Affiliations:** a Department of Chemistry, University of Iowa Iowa City IA 52242 USA tori-forbes@uiowa.edu; b Department of Civil and Environmental Engineering, University of Iowa Iowa City IA 52240 USA; c Center for Functional Nanomaterials, Brookhaven National Laboratory Upton NY 11973 USA

## Abstract

Rare earth elements (REEs) pose challenges for chemical separation due to similarities in charge and ionic radii. Developing materials with high metal specificity, such as molecularly imprinted polymers, could enhance selectivity, but there are limited details available to support the rational design of selective REE binding motifs. In the current study, structural characterization of REE–DOTAM (1,4,7,10-tetrakis(carbamoylmethyl)-1,4,7,10-tetraazacyclododecane) complexes was combined with density functional theory (DFT) and machine learning (ML) to provide specific structural descriptors that can be used to develop selective REE sites. Nineteen REE–DOTAM complexes were synthesized from mixed solvent systems (H_2_O/DMF, H_2_O/DMSO, and H_2_O/DMA) and characterized using single-crystal X-ray diffraction. The resulting structural data were used to construct perfect templates (fixed ligand geometries without further relaxation) and relaxed templates (fully geometry-optimized structures). DFT-derived substitution energies revealed that selectivity between heavy and light REEs could be achieved, with larger energetic penalties observed for the perfect templates. Machine learning analysis attributed selectivity primarily to hydration energies in the perfect templates, with twist angles as the dominant structural control in the relaxed systems. Additional analysis of the ML output suggested specific conditions that could be targeted for rationally designed DOTAM-based materials for improved REE selectivity.

## Abstract 2

Structural characterization of 19 rare earth element DOTAM complexes, Density Functional Theory (DFT) and Machine Learning (ML) methods were used to identify chemical descriptors for the rationally design of materials with molecular recognition.

## Graphical abstract

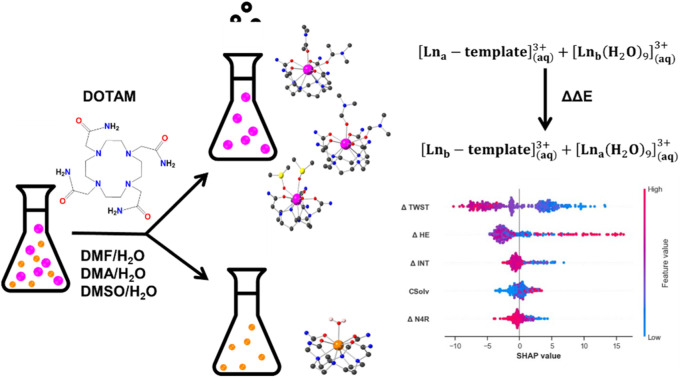

## Introduction

There is a significant demand for rare earth elements (REEs) in energy and technological applications; however, their similar chemistries (*i.e.* oxidation state, size, coordination geometries, *etc*.) prove challenging for separating these elements in pure form.^1^ Solvent extraction is the dominant industrial method for large-scale REE separations, where phosphonate (tri-*n*-butyl phosphate) and acidic organophosphorus-based extractants (P204, P507, *etc*.) are used to extract REEs from acidic aqueous solutions.^2–5^ While these systems have good selectivity for large scale separations, issues with chemical similarity between REEs require hundreds of extraction stages, complex processing, and large volumes of acidic waste streams for effective partitioning.^2,6^ Classical ion exchange processes use resins with sulfonate, carboxylate, or phosphonate groups for REE separations, where gradient elution produces the separated product.^2^ These systems, however, face challenges due to slow kinetics, column fouling, complex elution strategies, and volume of effluent that can cause difficulties in larger scale operations.^7–9^ Therefore, continued efforts are needed to develop separation strategies with higher REE selectivity, recyclability, and smaller waste streams.

Emerging strategies in REE separations focus on modern adsorbents with specific interest in creating substrates with molecular recognition capacity.^10,11^ Mesoporous silica, metal organic frameworks, and polymer substrates serve as scaffolds for organic functional groups in selective REE extraction, but they traditionally rely on tethering the active site to selectively bind the REE of interest. Many organic chelators used in REE recovery also bind to other elements in the periodic table, resulting in non-competitive binding and decreased selectivity in the separation.^12,13^ Polymer substrates offer additional opportunities as a scaffold due to flexible interactions with the organic chelators. These molecules can be tethered to the surface of a polymer substrate or incorporated into specific pore spaces that potentially create specific metal binding pockets that mimic those observed in biological molecules and can be used for enhanced separations. These polymer inclusion membranes or molecularly imprinted polymers are currently being evaluated as novel substrates for REE separations, using metal chelators such as EDTA or phosphonate-based extractants.^14,15^ In these materials, the chelated REE cation is first incorporated into the polymer matrix and then removed, leaving a cavity with specific binding affinity to the metal of interest. However, there is limited information on the rational design of these cavity spaces, particularly on the specific challenges associated with the chemical similarities between the REEs.

**Fig. 1:**
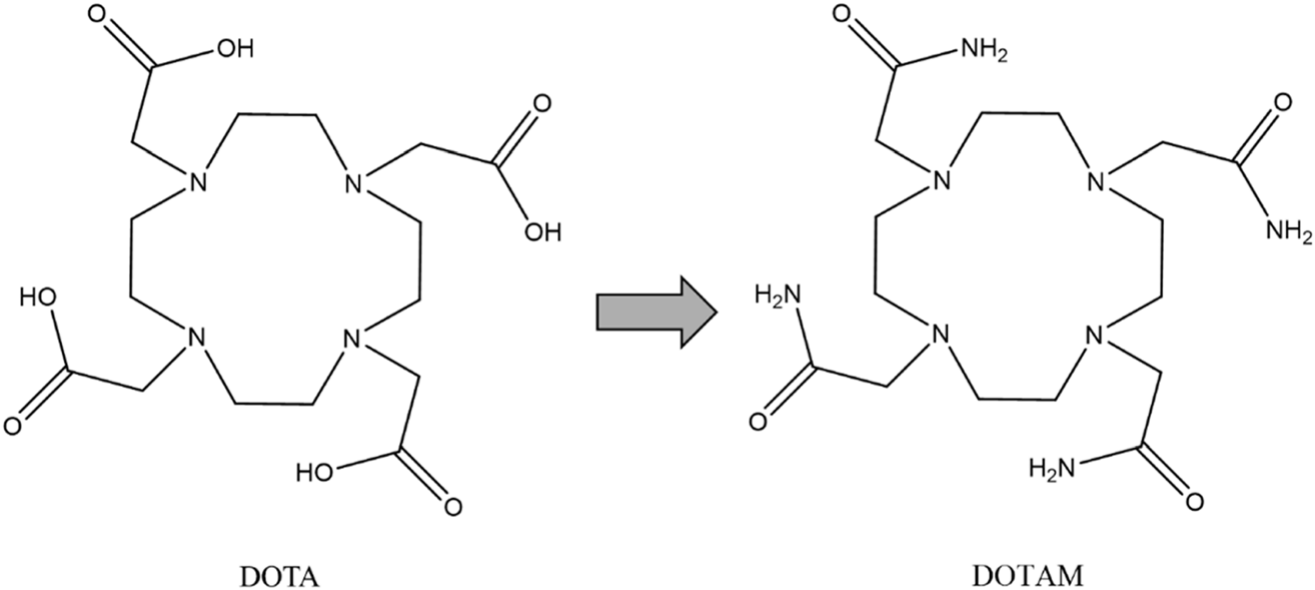
Structural representation of the well-known 1,4,7,10-tetraazacyclododecane-1,4,7,10-tetraacetic acid (DOTA) ligand used in REE chelation and the 1,4,7,10-tetrakis(carbamoylmethyl)-1,4,7,10-tetraazacyclododecane (DOTAM) molecule evaluated in the current study.

We are interested in rationally designing molecularly imprinted polymer substrates with higher separation factors for REEs. To start the design process, it is critical to identify specific structural and chemical parameters for the metal coordination environment to initially separate heavy rare earth (HRE) and light rare-earth (LRE) elements. For an initial system, we chose a 1,4,7,10-tetraazacyclododecane-1,4,7,10-tetraacetic acid (DOTA)-based extractant because it is commonly used as a metal chelator for radiopharmaceuticals due to its high stability constants for REEs.^16–18^ DOTA has not been extensively explored as an extractant for REE separations due to its slow kinetics at room temperature;^19–21^ thus, we chose a derivatized version, 1,4,7,10-tetrakis(carbamoylmethyl)-1,4,7,10-tetraazacyclododecane (DOTAM) because of higher p*K*_a_ and decreased stability constants (10–15 orders of magnitude lower than that of DOTA) may improve REE release (Fig. 1).^22^ In addition, we explored mixed solvent systems (H_2_O/DMF, H_2_O/DMSO, and H_2_O/DMA) that would be utilized in the synthesis of polymer inclusion membranes or molecularly imprinted polymers to evaluate the impact of solvent identity on metal chelation. Herein, nineteen different solid state coordination compounds were synthesized with rare earth elements across the lanthanide series (LRE = La^3+^, Ce^3+^, Nd^3+^; HRE = Eu^3+^, Tb^3+^, Dy^3+^, Y^3+^) and were characterized using single-crystal X-ray diffraction and Raman spectroscopy. Structural analysis of the solid-state materials provided descriptors that were then used in density functional theory and machine learning methods to assess key drivers for achieving molecular recognition by DOTAM-based polymer substrates.

## Results and discussion

### Structural characterization and detailed steric analysis of the REE–DOTAM complexes

**Fig. 2:**
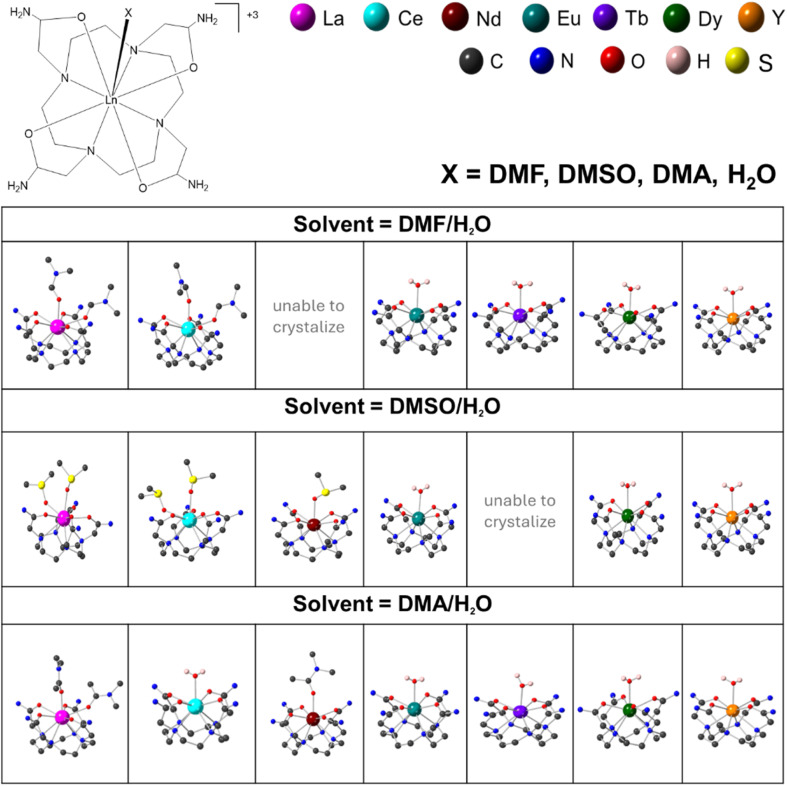
Successful crystallization and structural characterization of the REE–DOTAM compounds are represented as ball-and-stick models. The metal coordination complexes are shown with the solvent molecule (X) in the axial position. Two compounds could not be isolated and are shown as an empty box. The color for each atom type is detailed in the legend, and H atoms were removed from the models for clarity.

Nineteen of the possible 21 REE–DOTAM complexes were successfully crystallized and characterized (Fig. 2), but suitable crystals were not obtained for the Nd–DMF and Tb–DMSO systems. In the case of Nd–DMF, solid crystals were obtained, but the quality was not suitable for structure determination by single-crystal X-ray diffraction due to a high degree of twinning. For the Tb–DMSO experiments, high quality single crystals were produced, but structure solution determined that the DOTAM ligand did not form complex with the Tb^3+^ cation. Additional attempts to isolate the desired material in these systems were not successful.

The general metal coordination environment and structural features are similar throughout the crystallized REE–DOTAM compounds. The DOTAM ligand bonds to each metal center in an eight-coordinate fashion through the four nitrogen atoms in the tetra-aza core and the four oxygen atoms from the carbamoylmethyl branching arms, forming a cage-like structure around the metal cation (Fig. 2). Unlike the more commonly used DOTA ligand, DOTAM does not need to undergo deprotonation before bonding, leaving the ligand in a neutral state and resulting in a trivalent charge on the overall complex. Additional negative charge is required for neutrality of the solid-state compound, which is mediated by chloride or nitrate anions from the metal salt reagents. These anions, along with interstitial waters and solvent molecules, are observed in the second-coordination sphere around the chelated metal.

Each metal complex also contains an additional capping ligand(s) that binds to the metal center through an oxygen atom, with systematic variations in number and identity as we traverse the lanthanide series. Either water, DMF, DMSO, or DMA serve as the capping ligand, depending on the specifics of the REE and mixed solvent system (Fig. 2). The HRE (Eu^3+^, Tb^3+^, Dy^3+^, and Y^3+^) are capped with a single ligated water for all mixed solvent systems, resulting in the formation of nine-coordinated metal complexes. For the LRE (La^3+^, Ce^3+^, and Nd^3+^), there is significant variability in the identity and number of capping ligands. The La^3+^ cation is found as a ten-coordinate metal with two organic molecules (DMA, DMSO, or DMF) capping the metal center, and Nd^3+^ is observed as a nine-coordinate complex with DMA or DMSO coordinating in the axial position. Higher variability is observed for the Ce^3+^–DOTAM complexes with two DMF or DMSO molecules located within the first coordination sphere, to create 10-fold coordination, or one water molecule in the DMA/H_2_O system, resulting in a nine-coordinate metal center. These observed trends fit well with the overall hydration energy associated with the REEs, with values increasing from the LRE to HRE.^23^ There are known fluctuations, particularly between Nd^3+^ and Tb^3+^, and this is again captured in the variability in the capping ligand and challenges in the crystallization process.^24^

Previously reported structural data for the related REE–DOTA system were compared to the current REE–DOTAM system, and the analysis provides additional structural metrics that can be considered for the computational assessment. Crystal structures containing REE and DOTA were available for 10 of the 14 REE plus Y^3+^ and Sc^3+^ and were used by Viola-Villegas and Doyle in a detailed structural analysis.^21^ Their work indicated that the chelated REE–DOTA complexes all have a coordination number of 9, which occurs with an 8-fold coordination with DOTA in a square antiprismatic geometry and an additional water molecule bonding in the axial position. This is different from the current DOTAM system where there is variability in both the number and identity of the capping ligand. This variation may be influenced by the strength of the bonds to the branching arms or sterics of the carboxylate (DOTA) *versus* the carbamoylmethyl (DOTAM) functional groups on the ligand. Viola-Villegas and Doyle also indicated that the bond length between the REE cation and the axial water molecule may be an important structural descriptor because the bond strength influences the overall stability of the REE–DOTA complex. In addition, they suggested that the twist angle of the square prismatic geometry also influences the encapsulation of the metal, which is controlled by subtle differences in the ionic radii. While these structural parameters (coordination number, metal–nitrogen bonds lengths, metal–water bond lengths, and twist angle) have been previously identified as potential drivers in metal binding, their individual or combined importance has yet to be fully understood.

**Fig. 3:**
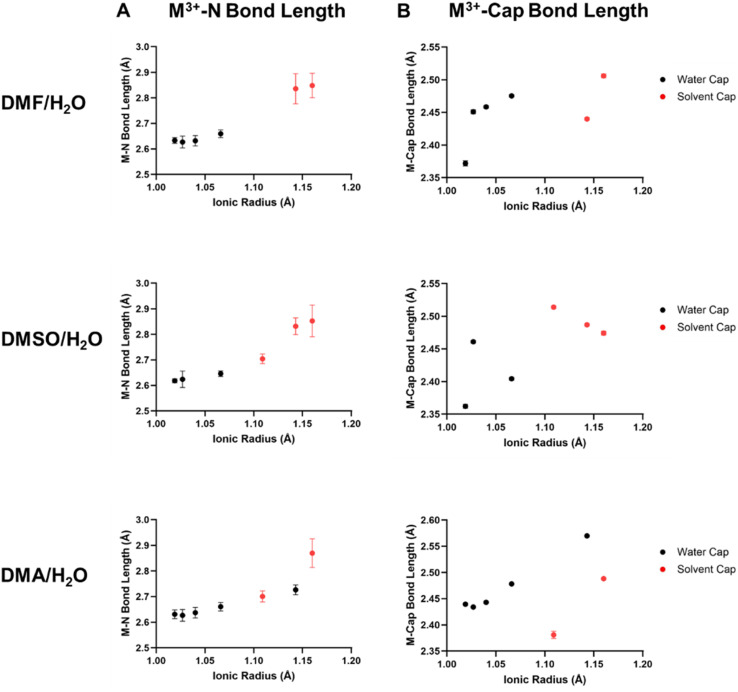
Metal–nitrogen bond lengths and the metal–capping oxygen bond lengths plotted as a function of the Ln^3+^ ionic radius.

To further evaluate these previously identified structural descriptors in the REE–-DOTAM system, we analyzed trends in bond distances across the lanthanide series. There is an increase in metal–nitrogen bond lengths as the ionic radius gets larger, with the average M–N bond length ranging from 2.62(1) to 2.90(6) Å for all systems (Fig. 3a and Table S5). The largest change in bond length is observed between Nd^3+^ and Eu^3+^ complexes, which is also the transition point from the heavy (HRE) to light (LRE) elements. Looking closer at the bond distances between the metal cation and the N atoms of the chelating DOTAM ligand, we note that the sterics and coordination number of the metal play a significant role. The 10-coordinated Ce^3+^ and La^3+^ systems capped by the larger solvent molecule have bond distances of 2.85(1) Å. When the metal is changed to Nd^3+^ and the coordination number drops to 9, the M–N bond length is approximately 2.70(2) Å. Moving farther along the lanthanides, we note that Eu^3+^, Tb^3+^, Dy^3+^, and Y^3+^ are all nine coordinate systems capped by water, with bond lengths between 2.60(0.01) and 2.66(0.02) Å. The complex crystallized from the Ce–DMF system (10 coordinate, capped by DMF) has an average metal–nitrogen bond length of 2.84(6) Å, which is similar to the value observed for the Ce–DMSO system (10-coordinate, capped by the larger DMSO) at 2.83(3) Å. However, when the capping ligand is water, (*i.e.* the Ce–DMA system (coordination number 9)), the metal–nitrogen bond length decreases to 2.73(2) Å.

Identity of the ligand and coordination number of the metal cation also exert influence on the metal-to-capping ligand distances. The average metal-to-capping oxygen bond length ranges from 2.36–2.57 Å for all systems (Fig. 3b and Table S5). In general, there is a lengthening of the bond distance as the ionic radius increases, although the correlation is not as strong as the data associated with the metal–nitrogen distances. This difference could be due to the different capping ligands (H_2_O, DMF, DMSO, and DMA), which contribute to the variations in sterics and bond strengths for each of the solvent systems.

It is important to note that even in the HRE systems (where the capping ligand is always water), there are instances where the bond distance varies for the same REE cation. An example of this is, for Y^3+^, although the capping ligand is consistently water, the bond distance is 2.372(4), 2.362(3), and 2.439(2) Å for the DMF/H_2_O (YDOTAMDMF), DMSO/H_2_O (YDOTAMDMSO) and DMA/H_2_O (YDOTAMDMA) systems, respectively. The M–N bond lengths within these systems also vary, but the magnitude is much less (0.02 *vs.* 0.1 Å), indicating that the second coordination environment may play a major role. Further evaluating the second coordination sphere environment, we note that the YDOTAMDMF and YDOTAMDMSO systems have two additional interactions with either Cl^−^ anions or H_2_O molecules, respectively. In the YDOTAMDMA system, there is only one H_2_O molecule interacting with the capping H_2_O in an asymmetric fashion and may be tied to the M–H_2_O bond elongation.

Raman spectroscopy (Fig. S8–S26 and Table S6) was used to further evaluate the impacts of the second coordination sphere on the axial solvent molecule, with the Y^3+^ again serving as a model for discussion. Rudolph and Irmer utilized the *ν*_1_ symmetric stretching mode (*ν*_1_) of the REE–(OH_2_) bond and correlated it to the bond distance for free REE hydrates in dilute aqueous perchlorate systems.^25^ The *ν*_1_ band for [Y(H_2_O)_8_]^3+^ was observed as a single feature at 384 cm^−1^, and the bond distance for the Y–OH_2_ bond is 2.35 Å. Similar *ν*_1_ frequencies are observed for YDOTAMDMF and YDOTAMDMSO at 385 and 387 cm^−1^, respectively, indicating that the hydration energy of the Y^3+^ cation has not been significantly perturbed by chelation. However, the YDOTAMDMA system is redshifted to 380 cm^−1^, suggesting bond elongation/weakening that may destabilize the metal chelate. This again highlights the importance of the second coordination environment in controlling metal bonding.

Last, the overall geometry and twist angle of the DOTAM cage structure were evaluated (Fig. S27–S30 and Table S7), with the twist angle being defined by Aime *et al.* as the angle between the oxygen and nitrogen planes of the chelator (SI, Fig. S27).^26^ With nine-coordinate HRE complexes, the cage structure can be identified as a square anti-prism geometry with twist angles ranging between 39(2) and 40(2)°. Only two of the LRE complexes form nine-fold coordination (NdDOTAMDMSO and CeDOTAMDMA), and in both cases they form the twisted square anti-prism geometry with twist angles of 24(4) and 23(3)°. For the other LRE systems, the cage structure becomes distorted because the oxygen plane is shifted to accommodate the additional solvent molecules; thus, we report maximum and minimum twist angles. The maximum twist angle ranges from 33 to 38° and follows a trend where DMA < DMSO < DMF. There is no significant trend associated with the minimum angle, which was measured between −7 and 10°.

Structural characterization of the REE–DOTAM complexes has provided a wealth of information regarding the subtle changes in bonding and coordination environment across the series. The study by Viola-Villegas and Doyle also provides possible relationships between structural metrics and overall stability of the metal chelate system.^21^ While some of these correlations may be relevant to the DOTAM system, others, such as how the strength of the M–H_2_O bond may influence protonation reactions with the branching arms, may be less important. Thus, we turned to DFT and ML methods to further analyze the importance of the subtle differences in the first coordination sphere on metal binding and selectivity and identify important structural descriptors that can be used to rationally design materials with molecular recognition.

### Computational assessment of the REE–DOTAM system

Initial DFT calculations were performed to assess the selective binding of the cavity template to REEs. In this case, the template is defined as the structural constraints brought by the DOTAM molecule and the capping solvent and was determined by computing ΔΔ*E* for eqn (1).

1 [Ln_a_–template]_(aq)_^3+^ + [Ln_b_(H_2_O)_9_]_(aq)_^3+^ → [Ln_b_–template]_(aq)_^3+^ + [Ln_a_(H_2_O)_9_]_(aq)_^3+^

**Fig. 4:**
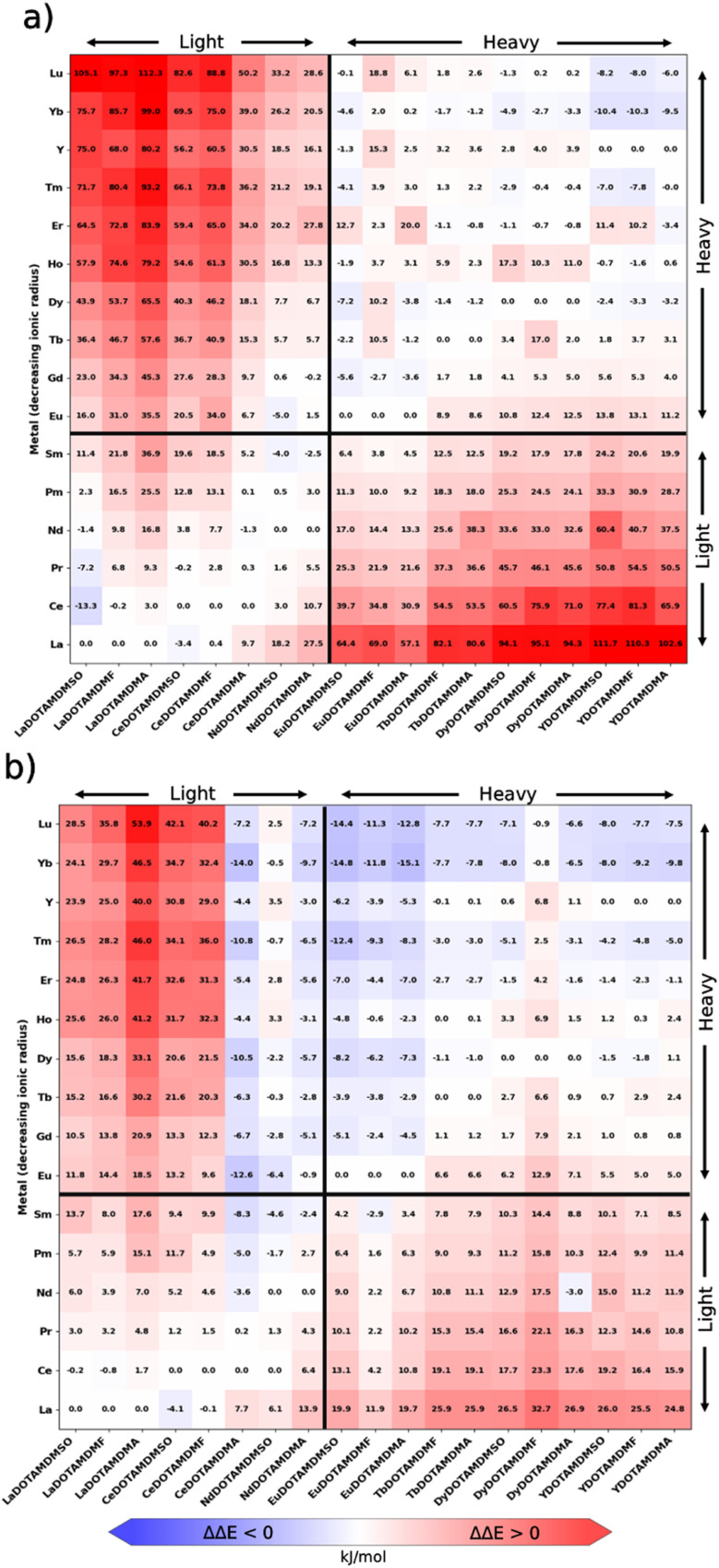
Computed ΔΔ*E* (kJ mol^−1^) for reaction given in eqn (1) for (a) perfect template and (b) relaxed template scenarios. Specific templates used for the models are provided on the *x*-axis and the metals used in the substitution are on the *y*-axis. Bolded lines separate the HRE and LRE elements. Red coloring in the box indicates when the ΔΔ*E* is positive, white occurs when ΔΔ*E* = 0, and blue indicates that the ΔΔ*E* is negative.

Here, ΔΔ*E* captures the change in energy that results from the exchange of one rare earth metal cation (Ln_a_^3+^) bound to the template with a solvated rare earth metal cation (Ln_b_^3+^). As shown in Fig. 4, the computed ΔΔ*E* values provide a systematic comparison of exchange energetics across the LRE and HRE and template environments. This approach takes advantage of error cancellation in electronic structure calculations, geometry optimization, and solvation modeling, and it has been used successfully to study metal extraction by solvent extraction.^27–31^ We calculated ΔΔ*E* rather than ΔΔ*G* to reduce the computational cost because the species on both sides of the equation are similar, which largely cancels entropy contributions. Prior work by Summers *et al.*^30^ has shown that this method does not meaningfully change DFT trends for lanthanide extraction, although it is noteworthy to mention that even “small” energetic differences matter in separation chemistry. For example, a ΔΔ*G* of 10 kJ mol^−1^ at 298 K corresponds to a separation factor (*α*) of ∼57 (*α* = *e*Δ^(Δ*G*)^/*RT*); thus, ΔΔ*E* values between 5 and 15 kJ mol^−1^ can translate into substantial differences in separation factors within these systems. Using ΔΔ*E* as the ML target focuses the model on relative selectivity and yields more chemically interpretable feature attributions for rational design.

To develop the REE models used in these calculations, the local coordination environment around the metal must be further evaluated. Taking the experimental data into consideration, we considered two scenarios for [Ln_a_–template]_(aq)_^3+^.

(a) The “Perfect template” uses the atomic positions from the crystal structure data to create the metal coordination environment for [Ln_a_–template]_(aq)_^3+^ and the energies of [Ln_a/b_–template]_(aq)_^3+^ were obtained from single-point calculations without prior geometry optimization. We refer to this as the perfect template because it represents an idealized situation in which all atoms of the template are fully restrained.

(b) The “Relaxed template” uses the atomic positions from the crystal structures to create the [Ln_a/b_−template]_(aq)_^3+^ complex, but these models were allowed to relax through geometry optimization. This case more closely reflects experimental conditions for metal–ligand binding, since there is no practical way to restrain every atom of the ligand and some rearrangement is expected due to metal–ligand interactions.

We consider both cases essential for building a fundamental picture of templating effects on rare earth metals. Importantly, the perfect template is not intended to represent a solution-phase minimum, since the crystallographic geometry may reflect contributions from counterions, lattice solvent, and crystal-packing interactions. Instead, comparison with the relaxed template allows us to evaluate how much of the predicted energetic differentiation is retained when these rigid structural constraints are removed. In both (i) and (ii), the solvated species [Ln_a/b_(H_2_O)_9_]_(aq)_^3+^ underwent geometry optimization before the energy calculations. These different scenarios were then used in the DFT calculations to calculate the ΔΔ*E* values.

In scenario (a), the ΔΔ*E* values indicate that the templates discriminate strongly between LRE (La^3+^ to Sm^3+^) and HRE (Eu^3+^ to Lu^3+^ plus Y^3+^). For the eight templates prepared with LRE cations, there are 80 possible cross substitutions by HRE cations (8 templates × 10 HRE, LRE → HRE) and 97.5% of them were energetically unfavorable (Fig. 3a). The only two cases with ΔΔ*E* < 0 involve Nd-templated complexes substituted by Eu^3+^ or Gd^3+^. Among the 40 possible substitutions with other LREs, 31 (77.5%) gave ΔΔ*E* > 0, showing some selectivity even within the LRE series. A similar pattern holds for HRE templated structures: all substitutions with LRE cations (11 templates × 6 LRE, HRE → LRE) are unfavorable. Within the HRE series, selectivity is weaker than in the LRE case. Across 11 HRE templated structures there are 99 possible substitutions with other HRE cations (HRE_*i*_ → HRE_*j*_), and 56 (about 56.6%) have ΔΔ*E* > 0. Among HRE templated complexes, EuDOTAMDMSO and the Y-templates show more favorability toward exchange or the weakest selectivity. In the case of the Y-templates, there is only unfavorable exchange with Eu^3+^-Tb^3+^ and Er^3+^, which likely relates to changes in ionic radii. EuDOTAMDMSO disfavors only Er^3+^ and all other HRE substitutions are favorable.

The Y-templates (YDMSO, YDMF, YDMA) can be used to highlight how subtle differences in the templates impact the energetic behavior. Favorability for exchange is similar for closely related HRE such as Lu^3+^ and Yb^3+^, with ΔΔ*E* values differing by only a few kJ mol^−1^ across YDMSO, YDMF, and YDMA templates (From Fig. 4: Lu^3+^: −8.2, −8.0, and −6.0 kJ mol^−1^, respectively; Yb^3+^: −10.4, −10.3, and −9.5 kJ mol^−1^). As the substituting cation changes to Tm^3+^, differentiation among the Y-templates becomes more evidence, with the YDMSO and YDMF complexes remaining unfavorable (−7.0 and −7.8 kJ mol^−1^), while YDMA is less unfavorable (−5.0 kJ mol^−1^). In the case of Er^3+^, the change is quite pronounced in the opposite direction, where YDMSO and YDMF are unfavorable (+11.4 and +10.2 kJ mol^−1^) and YDMA is energetically favorable for REE substitution (−3.4 kJ mol^−1^). Experimentally, the major structural difference in these systems was the REE–H_2_O bond length associated with the capping ligand. The computed ΔΔE results support the conclusion that the strength (and related length) of this bond can influence the energetics of the REE substitution.

In scenario (b), the relaxed template also shows partial separation between LRE and HRE cations. For the eight templates prepared with LRE cations, 62.5% of cross substitutions (LRE → HRE) are unfavorable, indicating that separation persists even after relaxation. Two differences from the perfect templates stand out in the case of the LRE substitution. First, the magnitude of unfavorable ΔΔ*E* values decreases by roughly 25% to 75%. Second, the CeDOTAMDMA and Nd templates lose their ability to separate LRE and HRE, whereas the other LRE templated systems retain their selectivity. Relaxation has little impact on the cross substitutions from HRE-templated to LRE cations (HRE → LRE): about 97% remain unfavorable, although the energetic penalties decrease by about 25% to 75% depending on the case. In contrast, selectivity among HRE cations declines markedly after relaxation, falling from 56.6% to 34.3% in the unrelaxed form. The exception is the DyDOTMADMF, where there is an increase in the ΔΔ*E* after relaxation within the (HRE → HRE).

Comparing the perfect template and relaxed template geometries by Root Mean Square Deviation (RMSD) shows substantial motion of the capping solvent during relaxation. The LRE templated complexes are capped by organic solvents (DMF, DMA, or DMSO), whereas the HRE templated complexes are capped by water. Most of the larger geometry changes occur in the organic capping ligands, giving the LRE templates an average RMSD of 0.373 Å, compared with 0.160 Å for the HRE templates (SI, Fig. S31). Across the substitution series, RMSD varies little, indicating consistent ligand relaxation with only subtle changes in bonding.

Taken together, the perfect and relaxed cases demonstrate that DOTAM templating clearly distinguishes between LRE and HRE ions, with the strongest effects under rigid, preorganized conditions, but is still observable (although weaker) after relaxation. Relaxation generally reduces the magnitude of unfavorable substitutions but preserves cross-class selectivity, while selectivity within a class diminishes. We note that energetic values on the order of 5–15 kJ mol^−1^ are comparable to typical uncertainties associated with DFT calculation and therefore should not be interpreted as quantitatively precise separation factors. Instead, these values are used here as relative energetic descriptors for identifying qualitative template–metal compatibility and selectivity trends. Although small free-energy differences can translate into appreciable separation factors at room temperature, the present ΔΔ*E* values should be viewed as indicators of possible energetic differentiation rather than definitive quantitative predictions of separation performance.

## Using machine learning for rational design

The goal of the machine learning analysis was to identify which structural descriptors govern the ΔΔ*E* trend across the rare earth series and to determine which experimental levers can be tuned to enhance the separation between LRE and HRE ions. The final descriptors and their definitions used to achieve this goal are listed in Table 1. Within a given template, the bond lengths, bond orders, and donor atom partial charges associated with the metal–DOTAM and metal–capping solvent bonds are largely dictated by the ionic radius of the metal cation and are therefore correlated. To reduce dimensionality within the system, we used average bond lengths, bond orders, and partial charges. When possible, descriptors were computed as differences (Δ) between [Ln_a_−template]_(aq)_^3+^ and [Ln_b_−template]_(aq)_^3+^. This choice offered two advantages: (i) it reduced the number of descriptors needed to represent both species by half and (ii) it helped cancel systematic errors in DFT electronic structure calculations and geometry optimization. In total, we selected nine descriptors for both the perfect template (scenario a) and relaxed template (scenario b).

**Table 1:** Summary of the descriptors used in the ML analysis. Here, abbreviations with the letter a refer to descriptors that are purely based on the template ([Ln_a_−template]_(aq)_^3+^), while abbreviations with the symbol Δ refer to engineered descriptors calculated from the difference between [Ln_a_−template]_(aq)_^3+^ and [Ln_b_−template]_(aq)_^3+^

Abbreviation	Description	Used in scenario
CSolv	Compresses the four solvent counts of the template into one integer *via* base-*B* positional encoding, where *B* = 3. The counts [DMA, DMF, DMSO, water] are weighted by [1, *B*, *B*^2^, *B*^3^] and dotted to yield a unique, reversible code	a and b
	DMA 1, 2 = 1,2 DMF 1, 2 = 3, 6; DMSO 1, 2 = 9,18, and water 1, 2 = 27, 54	
ΔHE	Change in hydration energies (kJ mol^−1^) ΔHE = ΔHE_Lnb_ − ΔHE_Lna_	a and b
	ΔHE_Ln_a/b__ = *E*_[Ln_a/n_(H_2_O)_9_](aq)_^3+^ − (*E*_[Ln_a/b_](g)_^3+^ + 9*E*_(H_2_O)(aq)_)^32^	
a AL/ΔAL	Average metal ligand bond length (Å)	a AL: a
		ΔAL: b
ΔAQ	Average Mayer partial charge on ligand donor atoms	a and b
ΔAB	Average Mayer bond order of the metal ligand bond	a and b
a TWST/ΔTWST	TWIST angle (°)	a TWST: a
		ΔTWST: b
a INT/ΔINT	Interplanar angle (°)	a INT: a
		ΔINT: b
a N4R/ΔN4R	RMSD of four nitrogen square from 90°	a N4R: a
		ΔN4R: b
a O4R/ΔO4R	RMSD of four oxygen square from 90°	a O4R: a
		ΔO4R: b

**Fig. 5:**
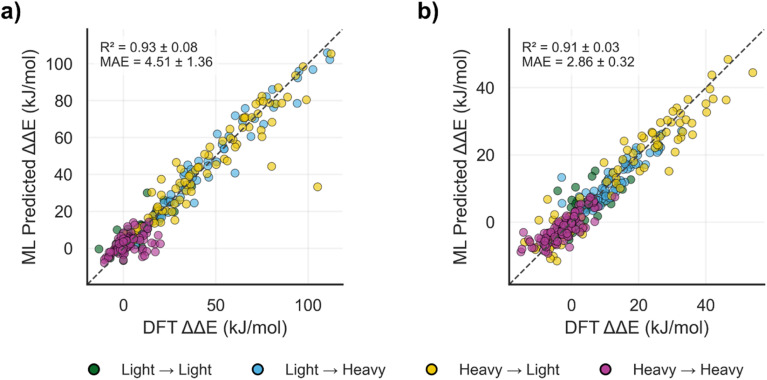
ΔΔ*E* prediction by CatBoost *vs.* DFT calculated ΔΔ*E* with the (a) perfect template [scenario (a)] and (b) relaxed template [scenario (b)]. Markers are colored by substitution class: green, LRE to LRE; blue, LRE to HRE; yellow, HRE to LRE; purple, HRE to HRE.

Across the models used for this study (Random Forest, XGBoost, LightGBM, and CatBoost), out-of-fold prediction of ΔΔ*E vs.* DFT calculated ΔΔ*E* plots followed an almost 1 : 1 line with tight dispersion, indicating low bias and good calibration. In both scenarios (a) and (b), the cross-fold SD of *R*^2^ and MAE were uniformly small for all models, indicating stable generalization. CatBoost showed the tightest variability on both metrics; its SD(*R*^2^) and SD(MAE) were among the lowest across models, highlighting consistent performance across outer folds (Fig. 5 and SI, Fig. S32 and S33). Learning curves were well behaved and largely monotonic, with training and validation performance converging as the sample size increased. These results indicate no evidence of data leakage, overfitting, or underfitting (SI, Fig. S34 and S35). Next, we ran a Y scramble test (50 times target permutation) using the same tuning and cross validation protocol. Permutation scores collapsed toward the null and were far worse than the true run, and no permuted replicate approached the accuracy of the real model. This confirms that performance arises from genuine structure rather than chance alignment (SI, Fig. S36 and S37). Taken together, these diagnostics show that the pipelines capture robust, reproducible relationships in the features for both scenario (a) and (b). We therefore conclude that the performance of the models is strong, with trustworthy out of sample accuracy suitable for interpretation.

We used SHAP analysis to identify the five descriptors most strongly associated with the ΔΔ*E* trend and quantify the relationship between each descriptor and ΔΔ*E*. To ensure the feature interpretations are consistent across cross-validation folds, we evaluated the stability of the mean |SHAP| values for the top five descriptors using *s* = 1/(1 + *cv*), where *cv* = SD(|SHAP|)/mean(|SHAP|). Values of *s* near one indicate a descriptor's effect is stable and the interpretation is reliable; values near zero indicate instability. In scenario (a), all models achieved *s* > 0.63, with Random Forest being the highest at *s* ≈ 0.73. In scenario (b), stability improved further (average *s* > 0.85), and CatBoost was the highest at *s* ≈ 0.91 (SI, Fig. S40 and S45). Taken together with prior validation checks, these results indicate that all four models perform well; however, we selected CatBoost as the primary model for discussion because of its consistently stronger predictive accuracy across substitution classes (LRE → LRE, LRE → HRE, HRE → LRE, and HRE → HRE).

**Fig. 6:**
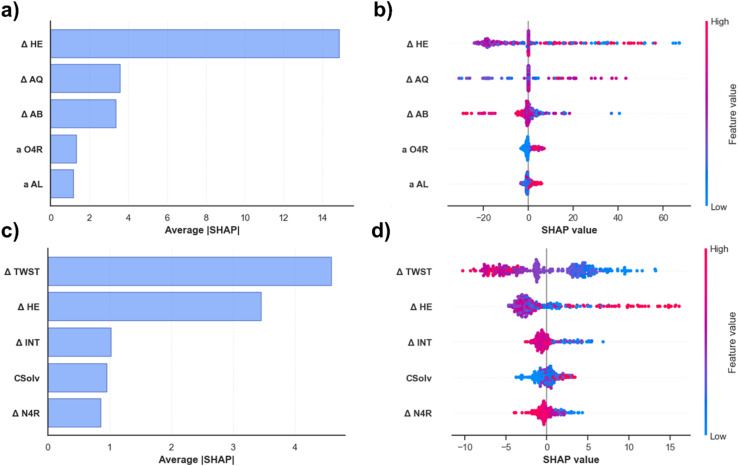
(a and c) The mean |SHAP| plot and (b and d) the beeswarm plot of the perfect template [scenario (a)] and the relaxed template [scenario (b)], respectively, calculated using CatBoost.

In the perfect template case (scenario a), CatBoost places the bulk of explanatory weight on ΔHE. It dominates the mean |SHAP| ranking and shows a wide, asymmetric spread in the beeswarm plot, consistent with a strong, near-monotonic effect on ΔΔ*E* (Fig. 6a and b). Points with larger ΔHE values fall on the positive SHAP side, indicating that more endothermic ΔHE pushes ΔΔ*E* upward (more endothermic), whereas exothermic ΔHE pulls ΔΔ*E* downward (more exothermic). The ΔAQ and ΔAB descriptors form a second tier with smaller magnitudes. For ΔAB, the descriptor skews positive overall, suggesting that when [Ln_b_−template]_(aq)_^3+^ exhibits stronger Ln–template bonding (higher bond order) than [Ln_a_−template]_(aq)_^3+^, ΔΔ*E* shifts in the favorable direction. By contrast, ΔAQ shows a mix of positive and negative contributions, pointing to context-dependent effects. The remaining terms (a O4R and a AL) cluster tightly around zero with short spreads, implying minor, local adjustments rather than primary drivers. A model-to-model check tells the same story: ΔHE ranks first by a wide margin in all four models, while mid-rank descriptors (ΔAB, ΔAQ, a AL, a INT, and a O4R) reshuffle modestly across algorithms, consistent with secondary roles sensitive to model bias and interaction handling (SI, Fig. S38 and S39). Overall, the dominance and directional consistency of ΔHE across models support it as the key lever for ΔΔ*E*, with ΔAQ and ΔAB providing smaller but meaningful incremental signals.

**Fig. 7:**
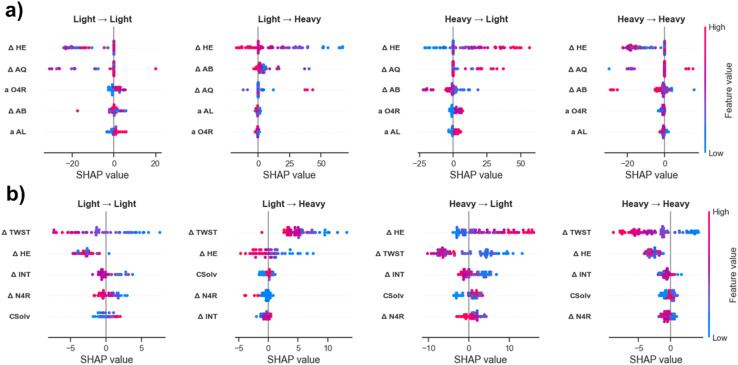
Beeswarm plots CatBoost for the top 5 variables under scenarios (a) and (b)*,* subdivided into four substitution types (LRE → LRE, LRE → HRE, HRE → LRE, and HRE → HRE).

Partitioning CatBoost's SHAP results by substitution type preserves the global pattern but reveals class-specific directionality (Fig. 7a and SI, S41). The ΔHE remains the dominant driver in every class, yet its sign depends on the receiver metal. With substitution of LRE → LRE, the larger ΔHE generally lowers ΔΔ*E* (more exothermic), whereas for LRE → HRE and HRE → LRE, larger ΔHE typically raises ΔΔ*E* (more endothermic). For HRE → HRE, ΔHE remains the dominant driver with a tighter spread, indicating a more uniform effect. Secondary terms rotate by class, with ΔAQ as the main complement in LRE → LRE and HRE → LRE, whereas ΔAB gains importance in LRE → HRE. Both act as smaller, context-dependent adjustments to the ΔHE baseline. Features a O4R and a AL remain close to zero across all classes, consistent with fine-tuning rather than control. Practically, the CatBoost SHAP analysis indicates that for the perfect template, ΔHE functions as the universal “control knob”, with ΔAQ or ΔAB modulating its impact depending on whether the substitution targets a light or heavy receiver.

In the relaxed template case (scenario b), CatBoost shifts the emphasis from ΔHE to ΔTWST, which now has the largest mean |SHAP| and a broad, asymmetric beeswarm plot (Fig. 6c and d). Low ΔTWST values fall mostly on the positive SHAP side, whereas high ΔTWST values trend negative, indicating that smaller twist changes raise ΔΔ*E* (more endothermic) and larger twist changes lower ΔΔ*E* (more exothermic). The ΔHE descriptor remains the second-strongest contributor with a largely positive, more endothermic ΔHE pushing ΔΔ*E* upward, mirroring its role in scenario (a) but with a secondary influence. Remaining features (ΔINT, CSolv, and ΔN4R) show shorter, centered spreads around zero, consistent with secondary, context-dependent adjustments rather than global control. All four models tell the same story, where ΔTWST and ΔHE are the top contributors, with only minor reshuffling among lower-impact descriptors. This cross-model agreement strengthens the conclusion that ΔTWST drives ΔΔ*E* in relaxed templates, with ΔHE providing a robust, directional secondary signal.

Breaking relaxed templates out by substitution class clarifies how ΔTWST and ΔHE operate across regimes (SI, Fig. S46). Overall, CatBoost ranks ΔTWST and ΔHE as the top contributors to ΔΔ*E* in all four classes. In LRE → LRE and LRE → HRE, ΔTWST clearly leads, while ΔHE plays a supporting role when the receiver is LRE. Thus, geometry changes dominate, and hydration enthalpy modulates the baseline. In HRE → HRE, the same geometry-first pattern holds (ΔTWST > ΔHE), indicating that heavy sites remain highly twist-sensitive even when both sides are heavy. The exception is HRE → LRE, where ΔHE overtakes ΔTWST; here, the hydration enthalpic change term captures the main penalty/benefit of moving from a heavy to a light environment. Across all classes, ΔINT, ΔN4R, and CSolv make smaller, localized adjustments, reinforcing that geometry (ΔTWST) and thermochemistry (ΔHE) govern the physics of ΔΔ*E* in the relaxed template. Practically, minimizing unfavorable twist changes is the most effective lever for LRE → LRE, LRE → HRE, and HRE → HRE substitutions, whereas tuning enthalpy is the most leveraged intervention for HRE → LRE moves.

The partitioned beeswarm plots refine the descriptors further within the relaxed template (Fig. 7b). For ΔTWST, distinct bands are created by class. In LRE → LRE and LRE → HRE, a dense, right-shifted band appears at moderate ΔTWST, while larger positive ΔTWST values trend negative, yielding a clear gradient from small-twist (positive SHAP, more endothermic) to large-twist (negative SHAP, more exothermic). HRE → HRE displays the tightest clustering around a negative SHAP mode, indicating strong sensitivity to twist penalties, such that once ΔTWST exceeds a modest threshold, contributions become consistently exothermic. In HRE → LRE, clustering of the ΔTWST data points is broader and more symmetric around zero, consistent with its secondary role in this class. In this case, the geometry still matters, but its sign depends more on the context set by ΔHE. For ΔHE, beeswarms are more class-separated and closer to monotonic. HRE → LRE exhibits a long, right-shifted plume (large positive SHAP), showing that more endothermic ΔHE strongly raises ΔΔ*E*, hence its top rank in this class. LRE → HRE also trends positive with a shorter spread, LRE → LRE centers near zero with mixed signs (supporting role), and HRE → HRE shows a compact, mildly negative mode, implying limited but directional influence once both sites are heavy.

Finally, we used the CatBoost SHAP results to define design windows that maximize the separation between LRE and HRE (Table 2). The goal was to use SHAP to identify intervals of each descriptor that satisfy two selectivity conditions: (i) under an LRE template, the window makes LRE → LRE favorable (ΔΔ*E* < 0) while making LRE → HRE unfavorable (ΔΔ*E* > 0) and (ii) under an HRE template, the window makes HRE → HRE favorable (ΔΔ*E* < 0) while making HRE → LRE unfavorable (ΔΔ*E* > 0). For each top descriptor, we smoothed the SHAP–feature relationship within each substitution class, located the SHAP zero-crossing (where the effect changes sign), and then took robust SHAP-supported ranges on the negative side (driving ΔΔ*E* < 0) and on the positive side (driving ΔΔ*E* > 0). Intersecting the LRE-negative range with the LRE → HRE-positive range yielded a light-selective window, whereas intersecting the HRE-negative range with the HRE → LRE-positive range yielded a heavy-selective window.

**Table 2:** Design windows to maximize light and heavy separation based upon CatBoost SHAP results

Scenario	Light selective	Heavy selective
	LRE → LRE: ΔΔ*E*↓	HRE → HRE: ΔΔ*E*↓
	LRE → HRE: ΔΔ*E*↑	HRE → LRE: ΔΔ*E*↑
Scenario (a): perfect template	ΔHE: −107.85 to −22.90 kJ mol^−1^	No overlap
	ΔAQ: −0.0023 to −0.014	
Scenario (b): relaxed template	ΔTWST: −1.24 to −0.35°	ΔINT: −0.10° to 0°
	ΔHE: −118.33 to −22.89 kJ mol^−1^	
	Cap solv: DMA 1 or DMA 2	

These SHAP-only windows identify computational descriptor ranges where the model predicts opposite outcomes for competing substitutions, providing theoretical design targets for prioritizing synthetic variables rather than direct experimental tolerances for individual binding sites (Table 2). This distinction is particularly important for narrow descriptors such as ΔTWST and ΔAQ. Molecularly imprinted polymers are expected to contain heterogeneous ensembles of binding sites, and current synthetic approaches are unlikely to enforce degree-level twist-angle control or narrowly tune ΔAQ in each binding pocket. Therefore, ΔTWST and ΔAQ are best viewed as mechanistic descriptors that identify the type of geometric preorganization and charge redistribution associated with light-selective binding, rather than as direct synthetic targets. From an experimental design perspective, the more actionable guidance comes from energetic descriptors and capping-solvent identity.

In the perfect template, the light-selective windows are identified for ΔHE where LRE templating is predicted to be stabilized while LRE → HRE substitutions are penalized. In the relaxed template, ΔHE and the DMA 1–2 capping environment provide experimentally relevant handles that can be explored through solvent selection and template identity. Within this framework, the SHAP trends provide concise qualitative guidance: sufficiently exothermic enthalpy and solvent/capping environments that promote small structural rearrangements are associated with LRE → LRE substitutions over LRE → HRE substitutions, whereas moving outside these energetic and capping-environment regimes may help discourage LRE capture. Thus, Table 2 provides a theoretical design map for prioritizing polymer-library synthesis and experimental validation, rather than a claim that polymer matrices can enforce exact geometric descriptor values.

To assess which of these design windows are most reliable, the stability of the SHAP attributions was evaluated across cross-validation folds using the stability score (*s*). In the perfect template, ΔHE exhibits substantially higher stability (*s* = 0.64) than ΔAQ (*s* = 0.31), indicating that hydration energy change control provides a more robust basis for rational design than ΔAQ (SI, Fig. S40). In contrast, feature stability improves markedly in the relaxed template (*s* = 0.87–94), suggesting that the associated design windows are more reliable after structural relaxation (SI, Fig. S45). Within this regime, the reliability follows the trend ΔINT ≈ ΔTWST ≈ cap solvent > ΔHE. These stability trends allow the design windows to be ranked according to their reliability, clarifying which rules provide predictable control and which require more careful considerations. The absence of overlapping heavy-selective windows for all five descriptors does not reduce the value of this framework. For HRE → HRE and HRE → LRE, the SHAP–feature curves frequently shift in the same direction across the supported domain, which prevents a single one-dimensional interval from driving one pathway negative (ΔΔ*E* < 0) while forcing the other positive (ΔΔ*E* > 0). In addition, some descriptors have limited leverage or sparse support, so their positive and negative regions are narrow and rarely align across classes. Importantly, the DFT results previously demonstrated a clear baseline separation in ΔΔ*E* between LRE and HRE in the rigid perfect template, with meaningful separation preserved after relaxation. The design windows therefore sharpen control where sign changes occur and help avoid regimes that erode selectivity. When no clear overlapping window appears for a single variable, the result points to a collective effect: separation arises from the joint action of multiple descriptors rather than any single feature. In such cases, multi-descriptor windows or joint tuning of ΔTWST, ΔHE, and secondary terms is the appropriate strategy, and the SHAP maps indicate which combinations should be tested first.

These results suggest that the solvent environment and polymer rigidity may be important variables for enhancing REE selectivity in DOTAM-based molecularly imprinted polymers. The use of DMSO, DMF, or DMA in appropriate concentrations could be used to tune the capping environment around the DOTAM–REE complex and potentially favor template geometries associated with LRE/HRE differentiation. However, preserving these templating effects after removal of the metal ion would require the second coordination sphere and polymer matrix to provide sufficient rigidity to maintain the preferred binding-site geometry. This requirement is particularly relevant for the perfect template case, which represents an idealized upper limit of structural preorganization. A similar concept applies to HRE selectivity, where polymer environments that preserve favorable twist or interplanar angles around the metal active site may help bias the binding pocket toward HRE-compatible geometries.

As stated earlier, the descriptor ranges and design windows identified here should be viewed as computational design targets for prioritizing future polymer synthesis and screening, rather than as experimentally validated tolerances or final synthetic rules. A direct experimental test would involve preparing DOTAM-based imprinted polymer libraries using selected REE templates while systematically varying solvent composition, functional monomer identity, crosslinking density, and polymerization conditions, followed by competitive REE uptake measurements to determine whether the predicted LRE/HRE selectivity trends are observed. While the present analysis is specific to DOTAM-derived coordination environments, the same workflow may be extendable to other macrocyclic chelators, including DOTA and DOTA/DOTAM variants, as they share related preorganized coordination motifs. However, the specific descriptor ranges and selectivity predictions will depend on pendant-arm, donor strength, capping environment, and ligand flexibility and therefore should be recalculated and experimentally validated before being generalized beyond the DOTAM systems studied here.

## Conclusion

In the current study, we evaluated the structural features of 19 REE–DOTAM coordination complexes with the goal of understanding the important chemical descriptors for the rational design of molecularly imprinted polymers with selectivity to REEs. Solvent systems for crystallization were varied to include water and DMF, DMSO, or DMA, which resulted in coordination of the organic solvents to the LRE, while the HRE consistently maintained a single water molecule in the capping site. This trend was correlated to the higher hydration energy of the HRE compared to the LRE. With the incorporation of the organic solvent, the coordination environment changed from 9-fold to 10-fold for La^3+^ and Ce^3+^. In these structural studies, DFT calculations were used to evaluate the energetics for LRE and HRE exchange in both the perfect and relaxed template environments. Overall, the perfect templates offered more energy penalties for exchange, suggesting the possibility for separation between HRE and LRE. These penalties were still maintained with the relaxed template, but the effect was less pronounced. Machine learning of this system provided a deeper understanding of the important chemical descriptors for metal binding, and SHAP analysis gave practical guidance on how to induce selectivity by providing rigidity to the cavity and working with mixed solvent systems. While this study did not test REE selectivity directly, it lays the groundwork for creating molecularly imprinted polymers with targeted goals for REE separations. It also provides a strategy to utilize the data gathered from structural analysis of metal chelators to inform DFT and ML workflows in support of the rational design of these materials.

## Author contributions

Nicole M. Shapiro: investigation, formal analysis, data curation, visualization, writing – original draft and editing; Harindu Rajapaksha: computational methodology, formal analysis, data curation, visualization writing – original draft and editing; Xiaohui Qu: computational methodology, formal analysis, writing – review & editing; Sara E. Mason: computational methodology, formal analysis, resources, writing – review & editing, supervision; David M. Cwiertny: formal analysis, resources, writing – review & editing, supervision, funding acquisition; Tori Z. Forbes: conceptualization, formal analysis, resources, visualization, writing – review & editing, supervision.

## Conflicts of interest

There are no conflicts of interest to declare.

## Supplementary Material

SC-OLF-D6SC03531K-s001

SC-OLF-D6SC03531K-s002

## Data Availability

The data supporting this article have been included as part of the supplementary information (SI). Supplementary information: optimized geometry data and CCDC numbers. The authors have cited additional references within the SI.^26,33–51^ See DOI: https://doi.org/10.1039/d6sc03531k. CCDC 2523220, 2523221, 2523222, 2523230, 2523231, 2523232, 2523233, 2523234, 2523235, 2523238, 2523239, 2523240, 2523241, 2523242, 2523243, 2523244, 2523245, 2523516 and 2523517 contain the supplementary crystallographic data for this paper.^52*a*–*s*^

## References

[cit1] Fouquet Y. and Martel-Jantin B., in Deep Marine Mineral Resources, ed. Y. Fouquet, D. Lacroix, Springer Netherlands, Dordrecht, 2014, pp. 55–64

[cit2] Gupta C. K., Krishnamurthy N. (2004). Int. Mater. Rev..

[cit3] Liu T., Chen J., Li H., Li K., Li D. (2019). Hydrometallurgy.

[cit4] Su W., Chen J., Jing Y. (2016). Ind. Eng. Chem. Res..

[cit5] Talan D., Huang Q. (2022). Miner. Eng..

[cit6] Haque N., Hughes A., Lim S., Vernon C. (2014). Resources.

[cit7] Beril Gönder Z., Kaya Y., Vergili I., Barlas H. (2006). Desalination.

[cit8] Abrams I. M. , in Studies in Environmental Science, ed. L. Pawlowski, Elsevier, 1982, Vol. 19, pp. 213–224

[cit9] Sharifian S., Wang N.-H. L. (2024). J. Environ. Chem. Eng..

[cit10] Kołodyńska D. , Fila D., Gajda B., Gęga J., Hubicki Z., in Applications of Ion Exchange Materials in the Environment, ed. Inamuddin, M. I. Ahamed and A. M. Asiri, Springer International Publishing, Cham, 2019, pp. 161–185

[cit11] Kondaurov R., Melnikov Y., Agibayeva L. (2023). Polymers.

[cit12] Hidayah N. N., Abidin S. Z. (2018). Miner. Eng..

[cit13] Pathapati S. V., Free M. L., Sarswat P. K. (2023). Processes.

[cit14] Bulin C., Guo T., Zheng R. (2025). Sci. Total Environ..

[cit15] Hovey J. L., Dittrich T. M., Allen M. J. (2023). J. Rare Earths.

[cit16] Stasiuk G. J., Long N. J. (2013). Chem. Commun..

[cit17] Tóth Ė., Brücher E. (1994). Inorg. Chim. Acta.

[cit18] Wang Y.-F., Han G. (2025). ChemistrySelect.

[cit19] Baranyai Z., Tircsó G., Rösch F. (2020). Eur. J. Inorg. Chem..

[cit20] Jackson J. A., Linero V., Bessen N. P., Nash K. L., Shafer J. C. (2021). Sep. Purif. Technol..

[cit21] Viola-Villegas N., Doyle R. P. (2009). Coord. Chem. Rev..

[cit22] Baranyai Z., Bányai I., Brücher E., Király R., Terreno E. (2007). Eur. J. Inorg. Chem..

[cit23] Moldoveanu G. A., Papangelakis V. G. (2012). Hydrometallurgy.

[cit24] Falco A., Neri M., Melegari M., Baraldi L., Bonfant G., Tegoni M., Serpe A., Marchiò L. (2022). Inorg. Chem..

[cit25] Rudolph W. W., Irmer G. (2017). Dalton Trans..

[cit26] Aime S., Botta M., Garda Z., Kucera B. E., Tircso G., Young V. G., Woods M. (2011). Inorg. Chem..

[cit27] Ali S. M., Pahan S., Bhattacharyya A., Mohapatra P. K. (2016). Phys. Chem. Chem. Phys..

[cit28] Keith J. M., Batista E. R. (2012). Inorg. Chem..

[cit29] Lehman-Andino I., Su J., Papathanasiou K. E., Eaton T. M., Jian J., Dan D., Albrecht-Schmitt T. E., Dares C. J., Batista E. R., Yang P., Gibson J. K., Kavallieratos K. (2019). Chem. Commun..

[cit30] Summers T. J., Taylor M. G., Augustine L. J., Janssen J., Perez D., Batista E. R., Yang P. (2025). JACS Au.

[cit31] Yang Y., Liu J., Yang L., Li K., Zhang H., Luo S., Rao L. (2015). Dalton Trans..

[cit32] Ciupka J., Cao-Dolg X., Wiebke J., Dolg M. (2010). Phys. Chem. Chem. Phys..

[cit33] Adamo C., Barone V. (1999). J. Chem. Phys..

[cit34] Neese F. (2022). WIREs Comput. Mol. Sci..

[cit35] Neese F. (2025). WIREs Comput. Mol. Sci..

[cit36] Perdew J. P., Burke K., Ernzerhof M. (1996). Phys. Rev. Lett..

[cit37] Lenthe E. v., Baerends E. J., Snijders J. G. (1993). J. Chem. Phys..

[cit38] van Wüllen C. (1998). J. Chem. Phys..

[cit39] Pantazis D. A., Chen X.-Y., Landis C. R., Neese F. (2008). J. Chem. Theory Comput..

[cit40] Pantazis D. A., Neese F. (2011). J. Chem. Theory Comput..

[cit41] Weigend F., Ahlrichs R. (2005). Phys. Chem. Chem. Phys..

[cit42] Grimme S., Antony J., Ehrlich S., Krieg H. (2010). J. Chem. Phys..

[cit43] Grimme S., Ehrlich S., Goerigk L. (2011). J. Comput. Chem..

[cit44] Marenich A. V., Cramer C. J., Truhlar D. G. (2009). J. Phys. Chem. B.

[cit45] Pedregosa F., Varoquaux G., Gramfort A., Michel V., Thirion B., Grisel O., Blondel M., Prettenhofer P., Weiss R., Dubourg V., Vanderplas J., Passos A., Cournapeau D., Brucher M., Perrot M., Duchesnay É. (2011). J. Mach. Learn. Res..

[cit46] Aime S., Barge A., Batsanov A. S., Botta M., Castelli D. D., Fedeli F., Mortillaro A., Parker D., Puschmann H. (2002). Chem. Commun..

[cit47] Tin Kam H. , in Proceedings of 3rd International Conference on Document Analysis and Recognition, Vol. 1, 1995, pp. 278–282

[cit48] Chen T. and Guestrin C., Proceedings of the 22nd ACM SIGKDD International Conference on Knowledge Discovery and Data Mining, arXiv, 2016, preprint, arXiv:1603.02754, 10.48550/arXiv.1603.02754

[cit49] Prokhorenkova L. O. , Gusev G., Vorobev A., Dorogush A. V. and Gulin A., arXiv, 2017, preprint, arXiv:1706.09516, 10.48550/arXiv.1706.09516

[cit50] Ke G. , Meng Q., Finley T., Wang T., Chen W., Ma W., Ye Q. and Liu T.-Y., in Proceedings of the 31st International Conference on Neural Information Processing Systems, Curran Associates Inc., Long Beach, California, USA, 2017, pp. 3149–3157

[cit51] Lundberg S. M. and Lee S.-i., arXiv, 2017, preprint, arXiv:1705.07874, 10.48550/arXiv.1705.07874

[cit52] (a) CCDC 2523220: Experimental Crystal Structure Determination, 2026, 10.5517/ccdc.csd.cc2qpm68

