# Supplementary material for: Structural analysis of lanthanide–DOTAM coordination complexes and use of machine learning to rationally design materials with molecular recognition for selective rare earth recovery
Source: Chem Sci. 2026 Jul 15. Online ahead of print. doi: 10.1039/d6sc03531k (PMC13418661; doi:10.1039/d6sc03531k)
Supplement: SC-OLF-D6SC03531K-s001 [file SC-OLF-D6SC03531K-s001.pdf]

Supplementary Information (SI) for Chemical Science.
This journal is © The Royal Society of Chemistry 2026

Supporting Information

Structural analysis of lanthanide-DOTAM coordination complexes and use of machine learning to rationally design molecular recognition for selective rare earth recovery

Nicole M. Shapiro,[a,b§] Harindu Rajapaksha,[a§] Xiaohui Qu,[c] Sara E. Mason,[c] David M. Cwiertny,[a,b] Tori Z. Forbes[a*]

[a]Department of Chemistry, University of Iowa, Iowa City, IA 52242, United States

[b]Department of Civil and Environmental Engineering, University of Iowa, Iowa City, IA 52242, United States

[c]Center for Functional Nanomaterials, Brookhaven National Laboratory, Upton, NY 11973, United States

[§]Co-first author; *Corresponding author: tori-forbes@uiowa.edu

Table of Contents

1. **Experimental Methods** 6

Synthesis of REE DOTAM Coordination Compounds 6

Structural Determination Using Single-Crystal X-ray Diffraction 6

Raman Spectroscopy 6

DFT Calculations 7

Machine Learning (ML) 7

2. **Additional Information for Experimental Methods** 8

Table S1: Summary of crystallographic yields 8

Table S2: Unit cell parameters and collection conditions for DMF/H_2O crystals 9

Table S3: Unit cell parameters and collection conditions for DMSO/H_2O crystals 10

Table S4: Unit cell parameters and collection conditions for DMA/H_2O crystals 11

3. **Additional Information for Results and Discussion** 12

Table S5: Summary of DOTAM coordination environments and bonding 12

Table S6: Raman peaks. Blue = DOTAM orange = DMSO 13

Figure S1: Asymmetric units of A) LaDOTAMDMF, B) LaDOTAMDMSO, and C) LaDOTAMDMA 14

Figure S2: Asymmetric units of A) CeDOTAMDMF, B) CeDOTAMDMSO, and C) CeDOTAMDMA 15

Figure S3: Asymmetric units of A) NdDOTAMDMSO and B) NdDOTAMDMA................16

Figure S4: Asymmetric units of A) EuDOTAMDMF, B) EuDOTAMDMSO, and C) EuDOTAMDMA..17

Figure S5: Asymmetric units of A) TbDOTAMDMF and B) TbDOTAMDMA 18

Figure S6: Asymmetric units of A) DyDOTAMDMF, B) DyDOTAMDMSO, and C) DyDOTAMDMA 19

Figure S7: Asymmetric units of A) YDOTAMDMF, B) YDOTAMDMSO, and C) YDOTAMDMA 20

Figure S8: Raman overlay of DOTAM and LaDOTAMDMF spectra for comparison Fitted Raman and fitting statistics of LaDOTAMDMF in the spectral region of interest (550-250 cm^{-1}) with a R^2 of 0.9991 and reduced $\chi 2$ of 1.6 x 10^{-5}...21

Figure S9: Raman overlay of DOTAM and CeDOTAMDMF spectra for comparison Fitted Raman and fitting statistics of CeDOTAMDMF in the spectral region of interest (500-300 cm^{-1}) with a R^2 of 0.9981 and reduced $\chi 2$ of 1.1 x 10^{-5}...22

Figure S10: Raman overlay of DOTAM and EuDOTAMDMF spectra for comparison Fitted Raman and fitting statistics of EuDOTAMDMF in the spectral region of interest (620-300 cm-1) with a R2 of 0.9993 and reduced χ2 of 6.0 x 10-5. 23

Figure S11: Raman overlay of DOTAM and TbDOTAMDMF spectra for comparison Fitted Raman and fitting statistics of TbDOTAMDMF in the spectral region of interest (600-300 cm-1) with a R2 of 0.9979 and reduced χ2 of 3.7 x 10-4. 24

Figure S12: Raman overlay of DOTAM and DyDOTAMDMF spectra for comparison Fitted Raman and fitting statistics of DyDOTAMDMF in the spectral region of interest (520-250 cm-1) with a R2 of 0.9997 and reduced χ2 of 4.9 x 10-5. 25

Figure S13: Raman overlay of DOTAM and YDOTAMDMF spectra for comparison Fitted Raman and fitting statistics of YDOTAMDMF in the spectral region of interest (520-300 cm^{-1}) with a R^2 of 0.9997 and reduced $\chi 2$ of 4.1 x 10^{-6}...26

Figure S14: Raman overlay of DOTAM and LaDOTAMDMSO spectra for comparison Fitted Raman and fitting statistics of LaDOTAMDMSO in the spectral region of interest (520-300 cm^{-1}) with a R^2 of 0.9971 and reduced $\chi 2$ of 2.7 x 10^{-5}...27

Figure S15: Raman overlay of DOTAM and CeDOTAMDMSO spectra for comparison Fitted Raman and fitting statistics of CeDOTAMDMSO in the spectral region of interest (520-300 cm^{-1}) with a R^2 of 0.9947 and reduced $\chi 2$ of 1.7 x 10^{-3}...28

Figure S16: Raman overlay of DOTAM and NdDOTAMDMSO spectra for comparison Fitted Raman and fitting statistics of NdDOTAMDMSO in the spectral region of interest (600-300 cm^{-1}) with a R^2 of 0.9994 and reduced $\chi 2$ of 2.0 x 10^{-6}..29

Figure S17: Raman overlay of DOTAM and EuDOTAMDMSO spectra for comparison Fitted Raman and fitting statistics of EuDOTAMDMSO in the spectral region of interest (520-250 cm^{-1}) with a R^2 of 0.9982 and reduced $\chi2$ of 1.7 x 10^{-4}. 30

Figure S18: Raman overlay of DOTAM and DyDOTAMDMSO spectra for comparison Fitted Raman and fitting statistics of DyDOTAMDMSO in the spectral region of interest (520-250 cm^{-1}) with a R^2 of 0.9965 and reduced $\chi2$ of 4.7 x 10^{-4}. 31

Figure S19: Raman overlay of DOTAM and YDOTAMDMSO spectra for comparison Fitted Raman and fitting statistics of YDOTAMDMSO in the spectral region of interest (520-300 cm^{-1}) with a R^2 of 0.9949 and reduced $\chi2$ of 1.8 x 10^{-3}. 32

Figure S20: Raman overlay of DOTAM and LaDOTAMDMA spectra for comparison Fitted Raman and fitting statistics of LaDOTAMDMA in the spectral region of interest (510-250 cm^{-1}) with a R^2 of 0.9971 and reduced $\chi2$ of 2.8 x 10^{-4}. 33

Figure S21: Raman overlay of DOTAM and CeDOTAMDMA spectra for comparison Fitted Raman and fitting statistics of CeDOTAMDMA in the spectral region of interest (500-300 cm^{-1}) with a R^2 of 0.9952 and reduced $\chi2$ of 5.3 x 10^{-3}. 34

Figure S22: Raman overlay of DOTAM and NdDOTAMDMA spectra for comparison Fitted Raman and fitting statistics of NdDOTAMDMA in the spectral region of interest (525-300 cm^{-1}) with a R^2 of 0.9990 and reduced $\chi2$ of 3.5 x 10^{-4}. 35

Figure S23: Raman overlay of DOTAM and EuDOTAMDMA spectra for comparison Fitted Raman and fitting statistics of EuDOTAMDMA in the spectral region of interest (520-300 cm^{-1}) with a R^2 of 0.9997 and reduced $\chi2$ of 5.6 x 10^{-4}. 36

Figure S24: Raman overlay of DOTAM and TbDOTAMDMA spectra for comparison Fitted Raman and fitting statistics of TbDOTAMDMA in the spectral region of interest (550-300 cm^{-1}) with a R^2 of 0.9979 and reduced $\chi2$ of 3.8 x 10^{-4}. 37

Figure S25: Raman overlay of DOTAM and DyDOTAMDMA spectra for comparison Fitted Raman and fitting statistics of DyDOTAMDMA in the spectral region of interest (600-300 cm^{-1}) with a R^2 of 0.9978 and reduced $\chi2$ of 4.8 x 10^{-4}. 38

Figure S26: Raman overlay of DOTAM and YDOTAMDMA spectra for comparison Fitted Raman and fitting statistics of YDOTAMDMA in the spectral region of interest (520-300 cm^{-1}) with a R^2 of 0.9988 and reduced $\chi2$ of 5.6 x 10^{-4}. 39

Table S7: The average and maximum/minimum twist angle of 9- and 10-coordinate Ln DOTAM complexes, respectively. Square anti-prism, twisted square antiprism, and distorted geometries are highlighted in pink, green, and purple, respectively. Angles were found using the angle tool in CrystalMaker 11. 40

Figure S27: The twist angle of the complexes is determined by the angle of rotation between the oxygen and nitrogen planes. A twist angle closer to 45° corresponds to a square antiprism geometry while a smaller twist angle corresponds to a twisted square antiprism geometry. 41

Figure S28: Top-down images of the six DMF complexes A) La, B) Ce, C) Eu, D) Tb, E) Dy, F) Y. Red boxes indicate the oxygen plane. Blue boxes indicate the nitrogen plane...............42

Figure S29: Top-down images of the six DMSO complexes A) La, B) Ce, C) Nd, D) Eu, E) Dy, F) Y. Red boxes indicate the oxygen plane. Blue boxes indicate the nitrogen plane.........43

Figure S30: Top-down images of the seven DMA complexes A) La, B) Ce, C) Nd, D) Eu, E) Tb, F) Dy, G) Y. Red boxes indicate the oxygen plane. Blue boxes indicate the nitrogen plane. 44

Figure S31: RMSD comparing the geometries of the scenario (a, *perfect template*) with scenario (b, *relaxed template*)..45

Figure S32: ΔΔE prediction by the four machine learning models vs DFT calculated ΔΔE under scenario (a), with *perfect template*. 46

Figure S33: ΔΔE prediction by the four machine learning models vs DFT calculated ΔΔE under scenario (b), with *relaxed template*. 47

Figure S34: Learning curves of the four machine learning models used under scenario (a), with *perfect template*. 48

Figure S35: Learning curves of the four machine learning models used under scenario (b), with *relaxed template*. 49

Figure S36: Y-Scramble test for the four machine learning models used under scenario (a), with *perfect template*. 50

Figure S37: Y-Scramble test for the four machine learning models used under scenario (b), with *relaxed template*. 51

Figure S38: Mean |SHAP| value for the top 5 variables under scenario (a), with *perfect template*..52

Figure S39: SHAP Beeswarm for the top 5 variables under scenario (a), with *perfect template*. 53

Figure S40: Stability of the SHAP top 5 feature across folds under scenario (a), with *perfect template*. The stability score was calculated by $s = 1(1 + cv),\ cv = SDSHAPmeanSHAP$ 54

Figure S41: Mean |SHAP| value with CatBoost for the top 5 variables under scenario (a), with *perfect template*, subdivided into four substitution types (LRE→LRE, LRE→HRE, HRE→LRE, and HRE→HRE)...55

Figure S42: Beeswarm plots CatBoost for the top 5 variables under scenario (a), with *perfect template*, subdivided into four substitution types (LRE→LRE, LRE→HRE, HRE→LRE, and HRE→HRE). 56

Figure S43: Mean |SHAP| value for the top 5 variables under scenario (b), with *relaxed template*..57

Figure S44: SHAP Beeswarm for the top 5 variables under scenario (b), with *relaxed template.* 58

Figure S45: Stability of the SHAP top 5 feature across folds under scenario (b), *with relaxed template*. The stability score was calculated by $s = 1(1 + cv),\ cv = SDSHAPmeanSHAP$ 59

Figure S46: Mean |SHAP| value with CatBoost for the top 5 variables under scenario (b), with *relaxed template*, subdivided into four substitution types (LRE→LRE, LRE→HRE, HRE→LRE, and HRE→HRE). 60

Figure S47: Beeswarm plots CatBoost for the top 5 variables under scenario (b), with *relaxed template*, subdivided into four substitution types (LRE→LRE, LRE→HRE, HRE→LRE, and HRE→HRE). 61

1. Experimental Methods

Synthesis of REE DOTAM Coordination Compounds

A 1:1 molar ratio of $LaCl_3$ (0.125mmol) (Fisher Scientific) and DOTAM (0.125mmol) (Macrocyclics) solids were placed to a glass scintillation vial, followed by addition of 15 mL of deionized water and 400µL of organic solvent (DMF, DMA, or DMSO). The resulting solution was then stirred for 30 minutes at ~60 °C until complete dissolution of all solid reagents. The vial was left uncapped in a chemical fume hood to slowly evaporate, whereby high quality, colorless crystals formed within two weeks with yields of 74.1(1.7) – 101.8(1.8)% based upon La. Other rare earth compounds were synthesized with the same metal:ligand ratio and organic solvents but substituting the REE salt ($Ce(NO_3)_3$, $NdCl_3$, $EuCl_3$, $Tb(NO_3)_3$, $Dy(NO_3)_3$, and YCl_3 purchased from Fisher Scientific) for a total of 21 crystallization experiments. Evaporation procedures and time for these compounds were similar and yields varied from 32.4(1.9)-103(1.6)% (Table S1) based upon the REE.

Structural Determination Using Single-Crystal X-ray Diffraction

Single crystals of the RE-DOTAM compounds were mounted on MiTeGen micromounts and X-ray diffraction data was collected at 100 K on a Bruker D8 Quest equipped with an Incoatec IµS 3.0 Microfocus X-ray source (Mo Kα, λ = 0.71073 Å) and a Photon III detector. ShelXT was used to determine the molecular structure via intrinsic phasing methods through the APEX 5 software package. REE and well-resolved C, N, O, S or Cl atoms were located in the initial structure solution, while the remainder were found following least squares refinement of the partial structural model. Hydrogen atoms were placed on the DOTAM and organic solvent molecules using a riding command. To place hydrogen atoms on the water molecules, the positions were located in the difference Fourier map and added using the HFIX command. In some structures (YDMF, LaDMSO, NdDMSO, NdDMA, TbDMA, DyDMA), disorder of the solvent and/or water molecules prevented placing all the hydrogen atoms. Relevant structural and refinement data, including unit cell parameters and collection conditions, are provided in Table S2-S4 and crystallographic information files (CIFs) can be obtained from the Cambridge Structural Database by requesting deposition numbers 2523220-2523222, 2523230-2523235, 2523238-2523245, 2523516-2523517.

Raman Spectroscopy

Raman spectroscopy on the single crystal material was collected on each crystal after single-crystal X-ray diffraction analysis. All experiments were performed on a Renishaw inVia confocal Raman microscope, equipped with a 785 nm laser and a 1200mm grating. The pinhole aperture, and thus the laser width, was three micrometers. Single crystals were isolated on separate glass slides, placed under a confocal microscope, and focused at 5x and 20x magnification to ensure alignment of the optics. WiRE 3.4 served as the operating software utilized to collect all raw data and spectra. Spectra were collected between 250 - 3000 cm^{-1} by averaging five accumulations with laser powers ranging from 10-100%. Incremental scans were used to optimize the laser power and maximum

operating power was 200 mW (laser power at 100%). Background subtracting and peak fitting were performed using the Origin 2025 software.

DFT Calculations

All DFT calculations were performed with ORCA 6.1 using the PBE0 hybrid functional.[33-36] Relativistic effects are included by Zeroth-Order Regular Relativistic Approximation (ZORA) in combination with ZORA-recontracted versions of the def2 basis sets.[37-38] All non-metal atoms are represented by the ZORA-def2-TZVP basis set while metal atoms are represented by the SARC-ZORA-TZVP basis set together with SARC/J coulomb-fitting auxiliary base sets.[39-41] All geometry optimizations were performed with default convergence criteria with set total Energy change, total RMS gradient, and total MAX gradient to $5e^{-6}$ au, $1e^{-4}$ au, and $3e^{-4}$, respectively. All SCF calculations were performed with tight convergence criteria, which set energy change to $1e^{-8}$ au. All calculations included Grimme's DFT-D3 dispersion correction with Becke-Johnson damping (D3BJ) and were performed using Universal Solvation Model (SMD) with water as the solvent.[42-44] For the Ln-template structures, the initial geometry was cleaved from the experimental crystal structures. Analysis of Orca results and extraction of descriptors was performed with Orca Python Interface (OPI).

Machine Learning (ML)

ML analysis was performed with tree-based ensemble methods in their default implementations. Hyperparameters were optimized with randomized search inside nested Cross Validation (CV) to obtain variance aware, leakage free performance estimates. In the outer loop, we used fivefold StratifiedKFold[45] on quantile bins of the continuous target to preserve its distribution across folds and to provide an unbiased estimate of generalization. Specifically, each outer CV split used approximately 80% of the data for training/model selection and 20% as a held-out test set. Splitting was performed at the level of individual template–metal–substitution-pair data points, and the held-out test fold was excluded from imputation, scaling, feature selection, hyperparameter optimization, and model fitting, ensuring that test-set $\Delta\Delta E$ values did not influence model selection. Within each outer training split, the inner loop ran RandomizedSearchCV[45] (num iter = 200) over the model and feature selection space using threefold CV repeated eight times with distinct shuffles, again stratified by target quantiles. This repetition reduces the sampling variance of the inner CV scores. Model selection applied a stability aware refit rule that maximized (mean inner CV score - $\alpha \cdot$standard deviation) with $\alpha = 1$, using negative MAE (Mean Absolute Error) as the optimization metric. This explicitly prefers configurations that are accurate and consistent across splits rather than ones that overfit a lucky partition. Each learning pipeline used median imputation, RobustScaler, and SelectKBest with k treated as a hyperparameter (k $\in$).[45-46] Model families included Random Forest[47], XGBoost[48], CatBoost[49], and LightGBM[50], with conservative, regularization-oriented search ranges. Final performance is reported as mean ± Standard Deviation (SD) across outer folds for R^2 and MAE. For model evaluation, we stacked the out-of-fold predictions from the fivefold outer CV and produced three diagnostics: (i) learning curves from refits of the tuned pipeline on stratified fractions of the training set, (ii) prediction

versus actual plots for the aggregated out-of-fold predictions, and (iii) a y scramble test that reran the same nested CV and tuning protocol on permuted targets (at least 50 permutations) to form a null distribution and compute an empirical p value for overfitting.

Model explainability used SHAP (SHapley Additive exPlanations) on the pipeline transformed feature computed fold wise on out-of-fold data.[51] We analyzed global importance with the mean absolute SHAP value and assessed feature stability with a cross-validation index. For each feature, we computed the across fold coefficient of variation of the absolute values, defined as the sample SD (ddof = 1) divided by the mean, and then mapped it to a bounded score s ($s = {}^{1}/_{(1+cv)}$, $cv = \frac{SD|SHAP|}{mean|SHAP|}$). Interpretation relied on SHAP dependence plots for directional effects and clustered SHAP correlation heat maps to identify co varying drivers. We report conclusions only when SHAP patterns were consistent across folds (high rank correlation and low variability) and aligned with the out-of-fold error profile.

2. Additional Information for Experimental Methods

Table S1: Summary of crystallographic yields

Solvent Environment	Rare Earth Element	Percent Yield
DMF/H_2O	La	100(1.8)
	Ce	83.4(1.7)
	Eu	101(2.9)
	Tb	55.5(2.0)
	Dy	101(2.0)
	Y	98.8(2.1)
DMSO/H_2O	La	74.1(1.7)
	Ce	103(1.6)
	Nd	95.4(1.4)
	Eu	53.4(2.6)
	Dy	95.0(1.8)
	Y	88.5(1.9)
DMA/H_2O	La	102(1.8)
	Ce	89.0(2.0)
	Nd	55.7(1.3)
	Eu	88.5(2.7)
	Tb	32.4(1.9)
	Dy	56.7(2.0)
	Y	101(2.2)

Table S2: Unit cell parameters and collection conditions for DMF/H_2O crystals

crystal	[La(DOTAM)(DMF)$_2$]·Cl_3·3H_2O	[Ce(DOTAM)(DMF)$_2$]·3NO_3·DMF	[Eu(DOTAM)(H_2O)]·Cl_3·6H_2O	[Tb(DOTAM)(H_2O)]·3NO_3·3H_2O	[Dy(DOTAM)(H_2O)]·3NO_3·3H_2O	[Y(DOTAM)(H_2O)]·Cl_3·6H_2O
chemical formula	$C_{22}H_{52}N_{10}O_9Cl_3La$	$C_{25}H_{52}N_{14}O_{16}Ce$	$C_{16}H_{44}N_8O_{11}Cl_3Eu$	$C_{16}H_{40}N_{11}O_{17}Tb$	$C_{16}H_{40}N_{11}O_{17}Dy$	$C_{16}H_{40}N_{11}O_{17}Dy$
crystal system	monoclinic	triclinic	triclinic	triclinic	triclinic	triclinic
space group	$P2_1$	$P\bar{1}$	$P\bar{1}$	$P\bar{1}$	$P\bar{1}$	$P\bar{1}$
a (Å)	9.9201(4)	12.1970(5)	9.8490(2)	10.1852(6)	10.2129(4)	9.8238(6)
b (Å)	14.2097(6)	12.5630(5)	12.3335(2)	10.3373(6)	10.3120(4)	13.7027(9)
c (Å)	12.967(5)	13.5579(6)	13.2181(2)	15.9774(10)	15.9709(7)	14.2044(8)
α (°)	90	90.245(2)	75.2620(10)	91.962(2)	92.135(2)	99.975(3)
β (°)	98.512(10)	109.329(2)	76.6480(10)	99.099(2)	99.227(2)	106.786(3)
γ (°)	90	94.617(2)	83.3250(10)	116.099(2)	116.246(2)	104.021(3)
V (Å^3)	1807.72(13)	1952.99(20)	1508.07(5)	1481.43(16)	1477.90(11)	1714.12(19)
T (K)	100	100	100	100	100	100
Z	2	2	2	2	2	2
R_{int}	0.0516	0.0672	0.0536	0.0294	0.0753	0.0756
R_1	0.0182	0.0250	0.0.170	0.0204	0.0305	0.0790
CCDC deposition number	2523220	2523221	2523222	2523516	2523230	2523231

Table S3: Unit cell parameters and collection conditions for DMSO/H_2O crystals

crystal	[La(DOTAM)(DMSO)$_2$]·Cl_3·6H_2O	[Ce(DOTAM)(DMSO)$_2$]·3NO_3·3H_2O·DMSO	[Nd(DOTAM)(DMSO)]·Cl_3·6H_2O	[Eu(DOTAM)(H_2O)]·Cl_3·6H_2O·DMSO	[Dy(DOTAM)(H_2O)]·3NO_3·2H_2O·DMSO	[Y(DOTAM)(H_2O)]·Cl_3·6H_2O·DMSO
chemical formula	$C_{20}H_{52}N_8O_{12}S_2Cl_3La$	$C_{22}H_{52}N_{11}O_{19}S_3Ce$	$C_{18}H_{34}N_8O_{12}SCl_3Nd$	$C_{18}H_{52}N_8O_{12}SCl_3Eu$	$C_{17}H_{44}N_{12}O_{17}SDy$	$C_{18}H_{52}N_8O_{12}SY$
crystal system	orthorhombic	triclinic	monoclinic	monoclinic	triclinic	monoclinic
space group	$Pna2_1$	$P\bar{1}$	$C2/c$	$P2_1/c$	$P\bar{1}$	$P2_1/c$
a (Å)	13.2632(7)	11.7950(5)	19.7079(6)	10.0951(2)	9.9192(3)	10.0787(4)
b (Å)	21.2334(12)	12.9291(7)	10.4681(3)	24.9601(5)	12.8564(3)	24.9229(9)
c (Å)	13.7569(8)	13.7126(6)	34.6188(12)	14.1368(3)	12.8602(3)	14.0661(5)
α (°)	90	90.822(2)	90	90	96.9830(10)	90
β (°)	90	90.136(2)	101.9740(10)	105.9920(10)	99.1530(10)	106.097(2)
γ (°)	90	103.772(2)	90	90	94.1020(10)	90
V (Å^3)	3874.3(4)	2030.79(17)	6986.6(4)	3424.26(12)	1600.18(7)	3394.7(2)
T (K)	100	100	100	100	100	100
Z	4	2	8	4	2	4
R_{int}	0.0665	0.0704	0.0584	0.0569	0.0599	0.0763
R_1	0.0237	0.0324	0.0325	0.0286	0.0380	0.0506
CCDC deposition number	2523232	2523233	2523234	2523235	2523238	2523239

Table S4: Unit cell parameters and collection conditions for DMA/H_2O crystals

crystal	[La(DOTAM)(DMA)$_2$]·Cl_3·$3H_2O$	[Ce(DOTAM)(H_2O)]·$3NO_3$·$2H_2O$	[Nd(DOTAM)(DMA)$_2$] ·Cl_3·H_2O·(DMA)$_2$	[Eu(DOTAM)(H_2O)]·Cl_3·$7H_2O$	[Tb(DOTAM)(H_2O)]·$3NO_3$·$3H_2O$	[Dy(DOTAM)(H_2O)]·$3NO_3$·$3H_2O$	[Y(DOTAM)(H_2O)]·Cl_3·$7H_2O$
chemical formula	$C_{24}H_{56}N_{10}O_9Cl_3La$	$C_{16}H_{38}N_{11}O_{16}Ce$	$C_{28}H_{41}N_{11}O_8Cl_3Nd$	$C_{16}H_{46}N_8O_{11}Cl_3Eu$	$C_{16}H_{38}N_{11}O_{17}Tb$	$C_{16}H_{38}N_{11}O_{17}Dy$	$C_{16}H_{46}N_8O_{11}Cl_3Y$
crystal system	triclinic	monoclinic	monoclinic	triclinic	triclinic	triclinic	triclinic
space group	$P\bar{1}$	$P2_1/n$	$P2_1$	$P\bar{1}$	$P\bar{1}$	$P\bar{1}$	$P\bar{1}$
a (Å)	12.9225(8)	10.6385(2)	12.3671(8)	9.8585(3)	10.1984(4)	10.2057(3)	9.8204(2)
b (Å)	12.9521(8)	23.5292(5)	13.1787(10)	12.3341(3)	10.4214(3)	10.3694(3)	12.2636(3)
c (Å)	13.7286(8)	12.0379(3)	13.7529(9)	13.2242(5)	15.9176(6)	15.9334(4)	13.2286(3)
α (°)	68.412(2)	90	90	75.2750(10)	91.5730(10)	91.854(2)	75.5350(10)
β (°)	69.340(2)	103.3680(10)	110.110(2)	76.6490(10)	99.1090(10)	99.223(2)	76.5950(10)
γ (°)	60.384(2)	90	90	83.2920(10)	116.1920(10)	116.2480(10)	83.2030(10)
V (Å^3)	1815.5(2)	2931.63(11)	2104.8(3)	1510.28(8)	1489.82(9)	1482.58(7)	1497.54(6)
T (K)	100	100	100	100	100	100	100
Z	2	4	2	2	2	2	2
R_{int}	0.0540	0.0530	0.0892	0.0612	0.0564	0.0665	0.0733
R_1	0.0195	0.0238	0.0500	0.0132	0.0237	0.0421	0.0429
CCDC deposition number	2523240	2523241	2523242	2523243	2523517	2523244	2523245

3. Additional Information for Results and Discussion

Table S5: Summary of DOTAM coordination environments and bonding

REE	Coordination Number	Capping Ligand	Average M-N Bond Length (Å)	M-Cap Bond Length (Å)
**DMF/H_2O**				
La	10	DMF	2.8480 (0.05)	2.506 (3)
Ce	10	DMF	2.8355 (0.06)	2.4399 (2)
Eu	9	H_2O	2.6594 (0.02)	2.4753 (2)
Tb	9	H_2O	2.6318 (0.02)	2.4584 (2)
Dy	9	H_2O	2.6270 (0.02)	2.451 (3)
Y	9	H_2O	2.6330 (0.01)	2.372 (4)
**DMSO/H_2O**				
La	10	DMSO	2.8523 (0.06)	2.474 (3)
Ce	10	DMSO	2.8313 (0.03)	2.487 (2)
Nd	9	DMSO	2.7040 (0.02)	2.514 (2)
Eu	9	H_2O	2.6459 (0.01)	2.4042 (2)
Dy	9	H_2O	2.6240 (0.03)	2.461 (2)
Y	9	H_2O	2.6183 (0.01)	2.362 (3)
**DMA/H_2O**				
La	10	DMA	2.8692 (0.06)	2.4883 (1)
Ce	9	H_2O	2.7264 (0.02)	2.5698 (2)
Nd	9	DMA	2.7005 (0.02)	2.381 (7)
Eu	9	H_2O	2.6606 (0.02)	2.478 (1)
Tb	9	H_2O	2.6373 (0.02)	2.443 (2)
Dy	9	H_2O	2.6268 (0.02)	2.434 (2)
Y	9	H_2O	2.6310 (0.02)	2.439 (2)

Table S6: Solid State Raman spectral features in the spectral window of interest (200-500 cm^{-1}) for the REE-DOTAM compounds.

**DMF/H_2O**						**DMSO/H_2O**						**DMA/H_2O**						
**La**	**Ce**	**Eu**	**Tb**	**Dy**	**Y**	**La**	**Ce**	**Nd**	**Eu**	**Dy**	**Y**	**La**	**Ce**	**Nd**	**Eu**	**Tb**	**Dy**	**Y**
477	472	603	560	490	496	475	495	587	537	492	493	489	486	511	492	537	548	494
470	444	555	542	475	488	451	479	558	490	387	387	469	478	503	482	488	538	485
441	434	530	536	450	385	441	471	537	444	379	380	440	461	457	446	388	492	446
435	417	488	490	390	374	419	444	486	380	307		379	445	445	388	379	484	391
418	383	439	480	380	361	396	418	445	364			359	418	394	378	368	389	380
384	358	388	389	368		385	397	429	349			310	411	323	356		380	374
359		376	379	355		361	379	404	309			297	377	314			369	
328		365	367	341		349	362	387				276	332					
295		354		288		328	351	377					323					
271		338		267		324	332	359										
		316				310	310	343										
								324										

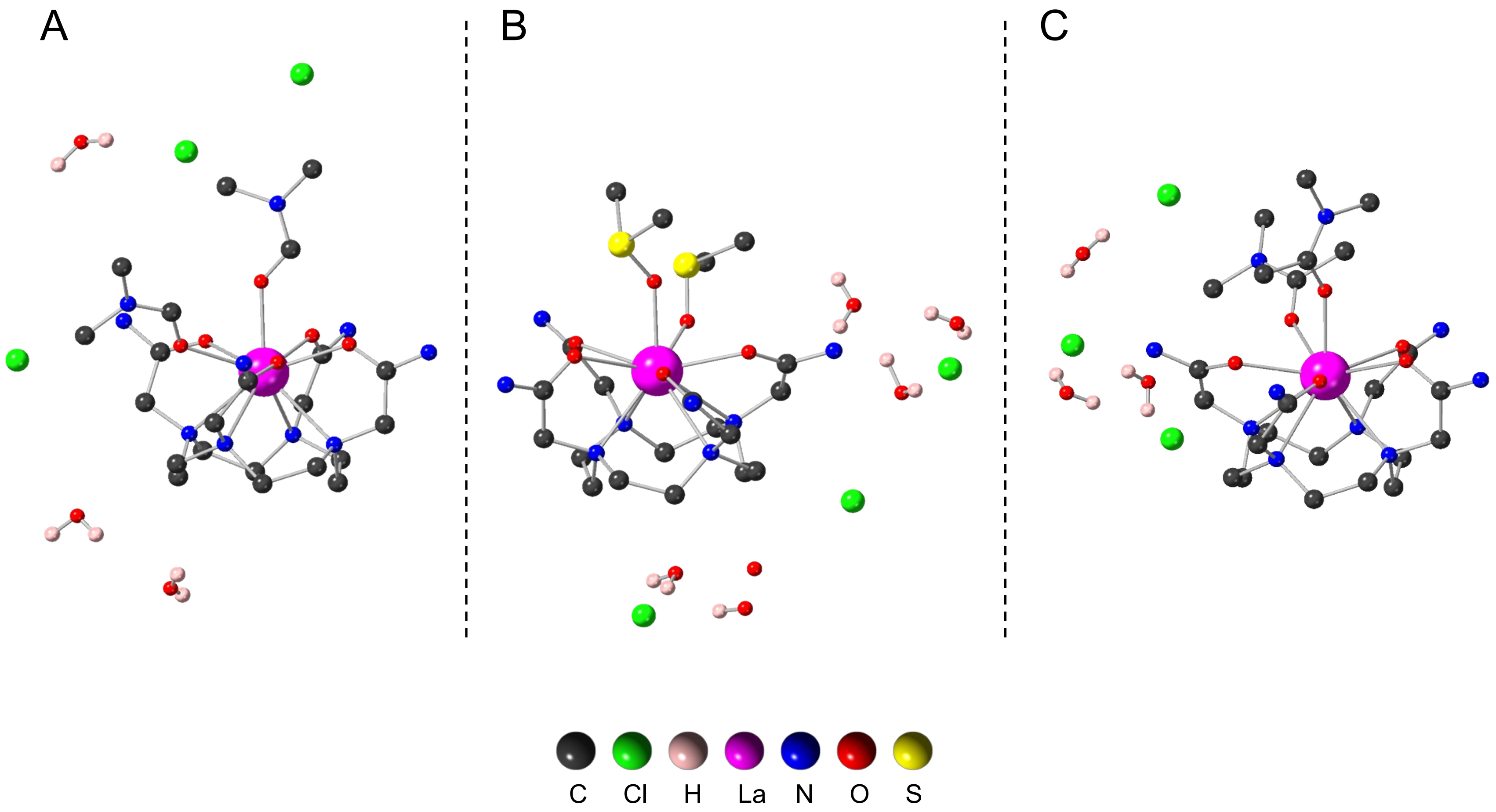

Figure S1: Asymmetric units of A) LaDOTAMDMF, B) LaDOTAMDMSO, and C) LaDOTAMDMA

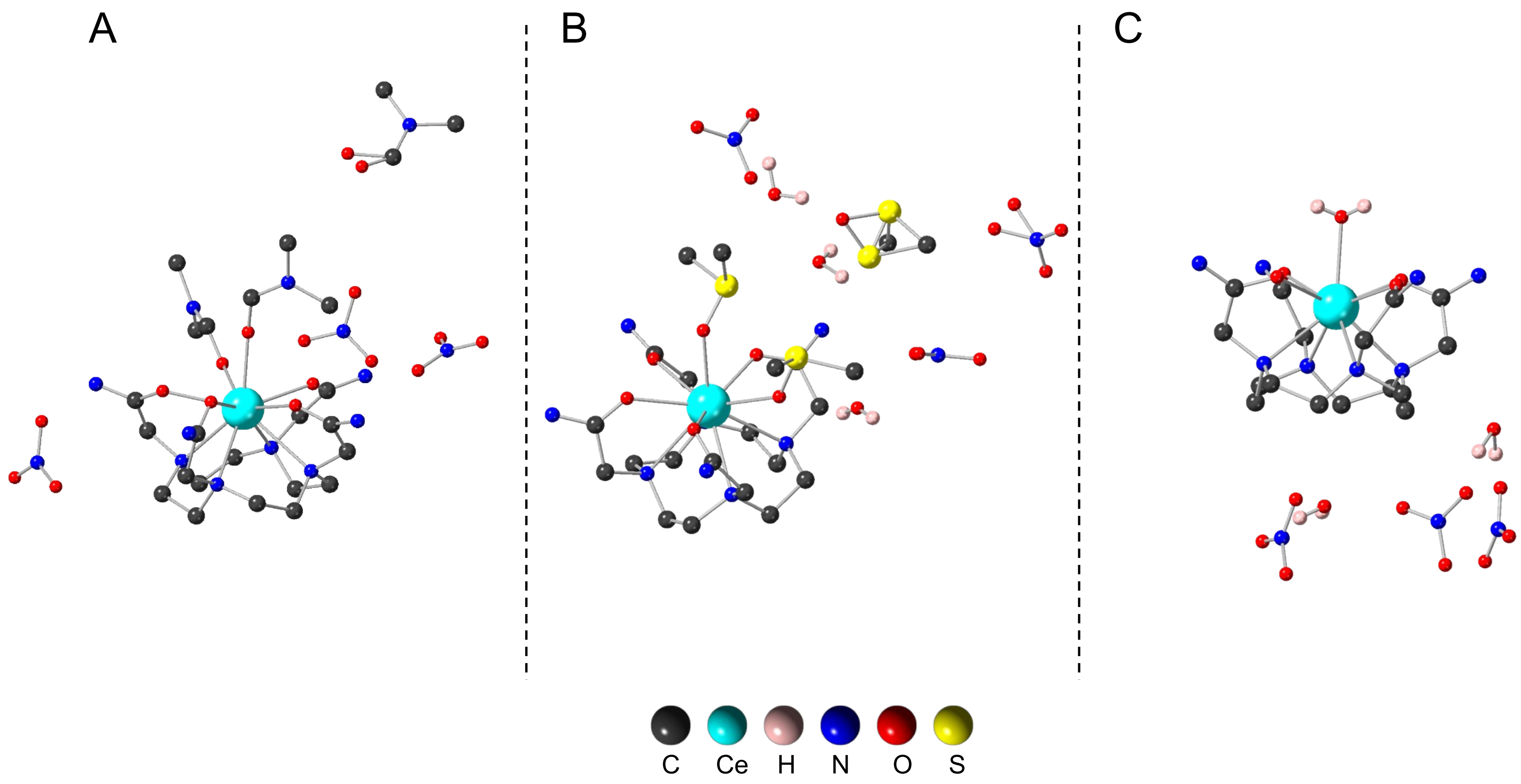

Figure S2: Asymmetric units of A) CeDOTAMDMF, B) CeDOTAMDMSO, and C) CeDOTAMDMA

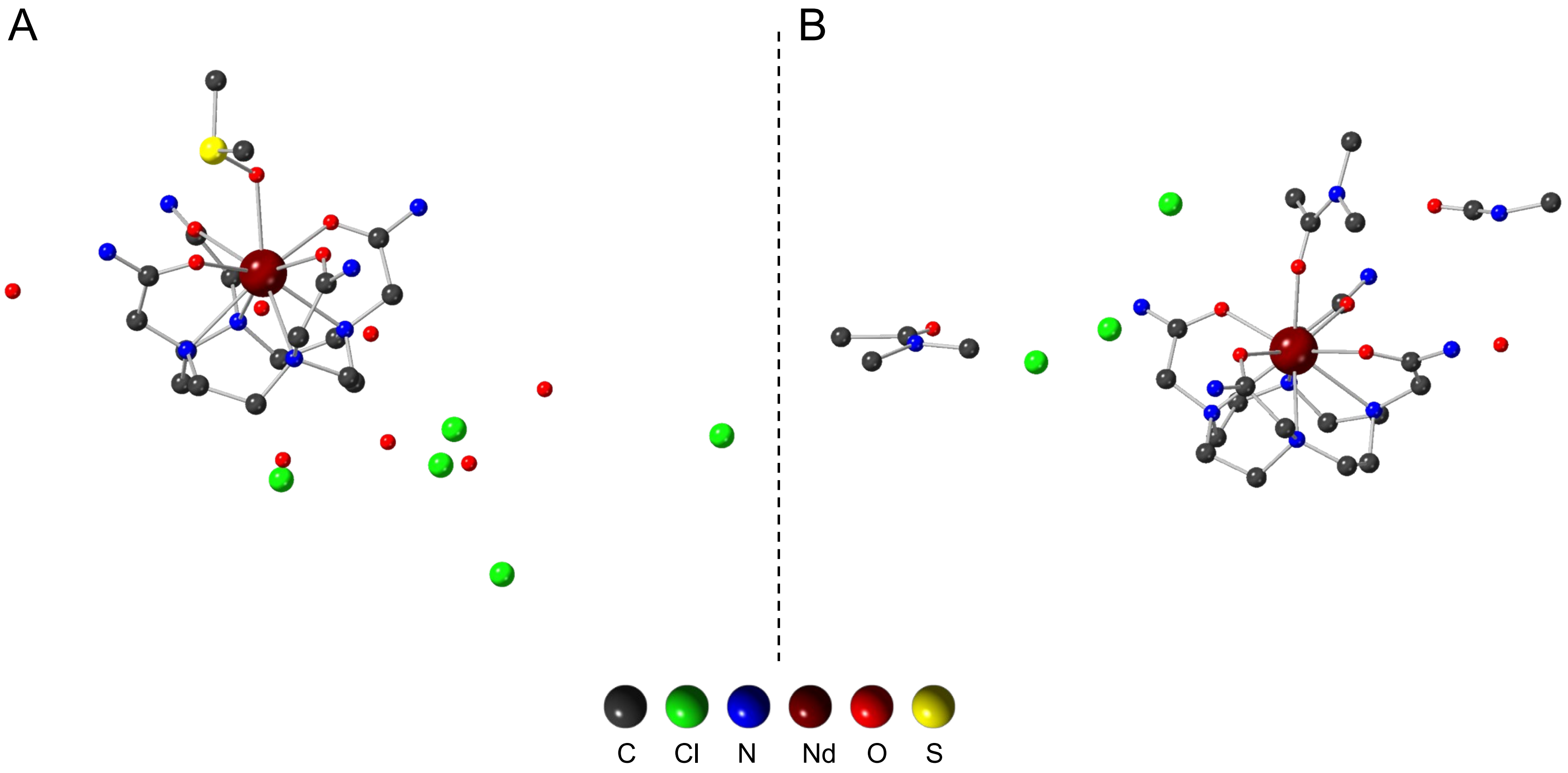

Figure S3: Asymmetric units of A) NdDOTAMDMSO and B) NdDOTAMDMA

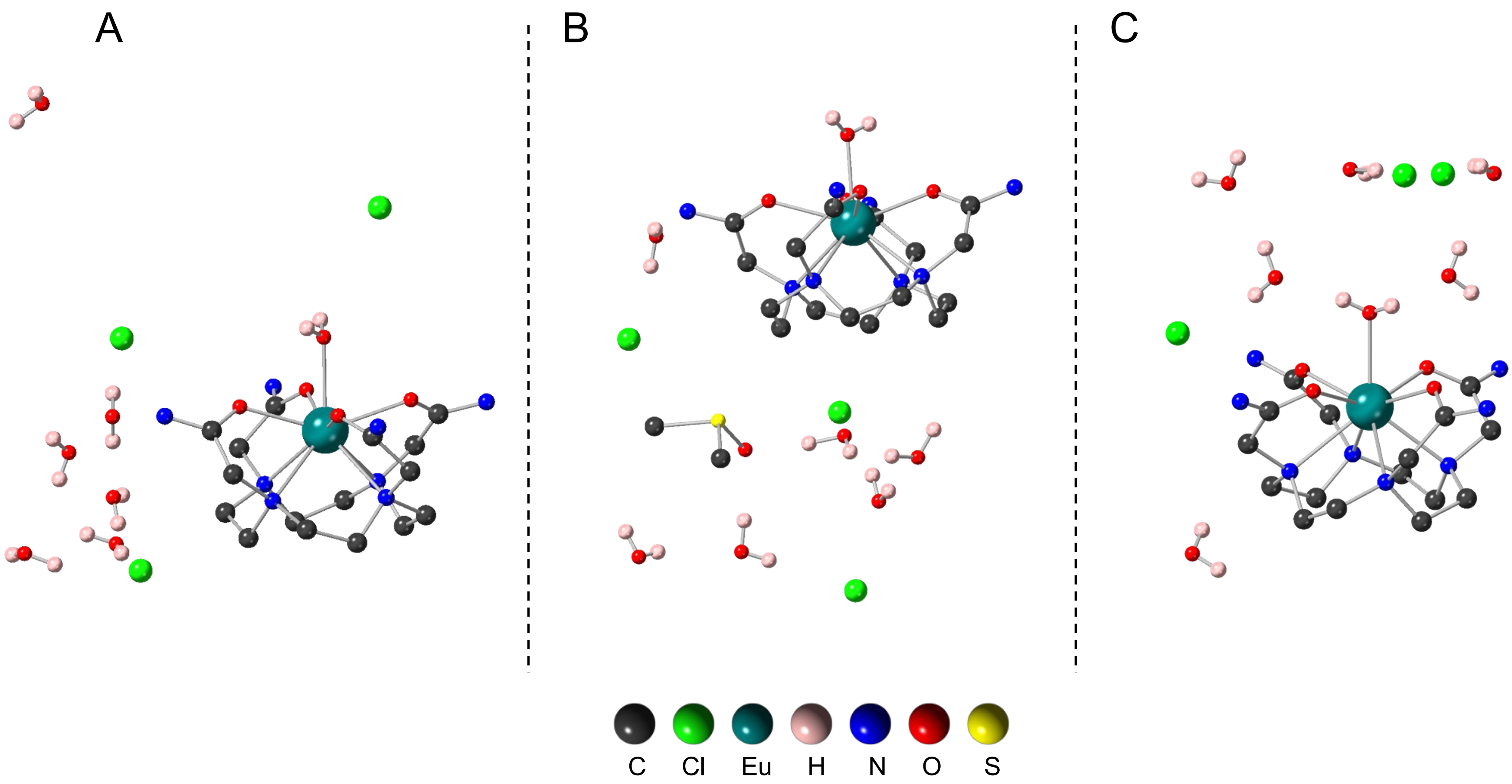

Figure S4: Asymmetric units of A) EuDOTAMDMF, B) EuDOTAMDMSO, and C) EuDOTAMDMA

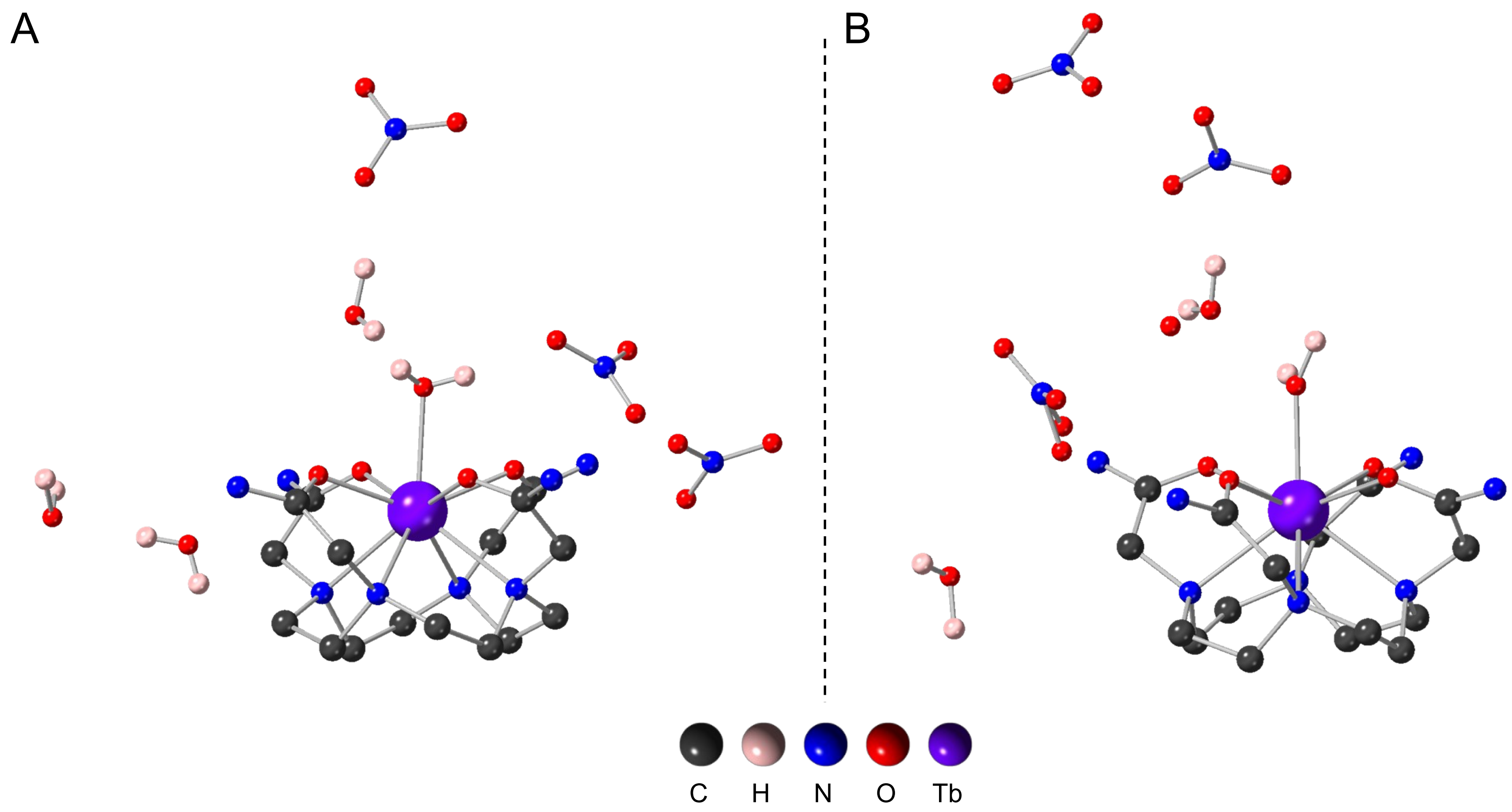

Figure S5: Asymmetric units of A) TbDOTAMDMF and B) TbDOTAMDMA

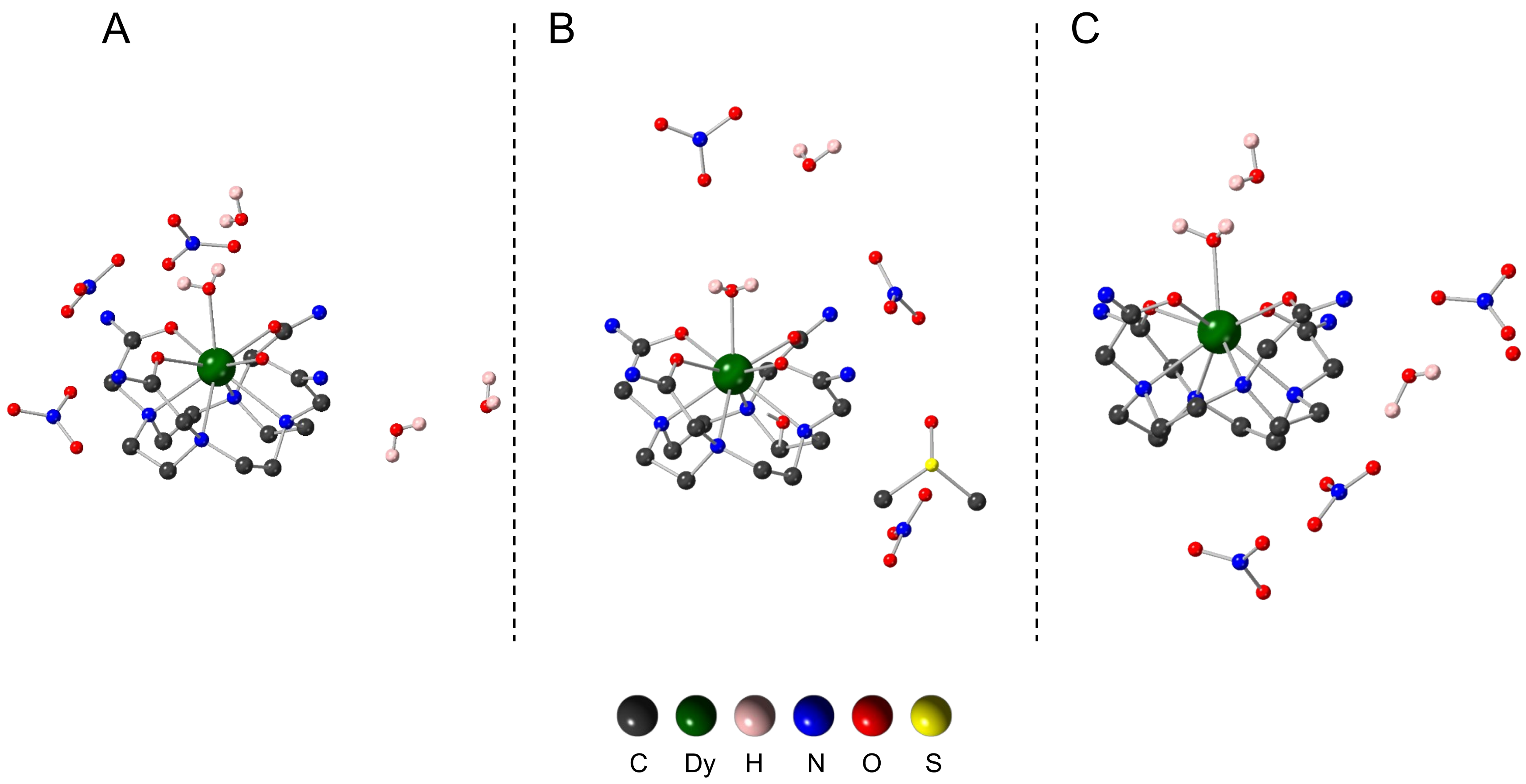

Figure S6: Asymmetric units of A) DyDOTAMDMF, B) DyDOTAMDMSO, and C) DyDOTAMDMA

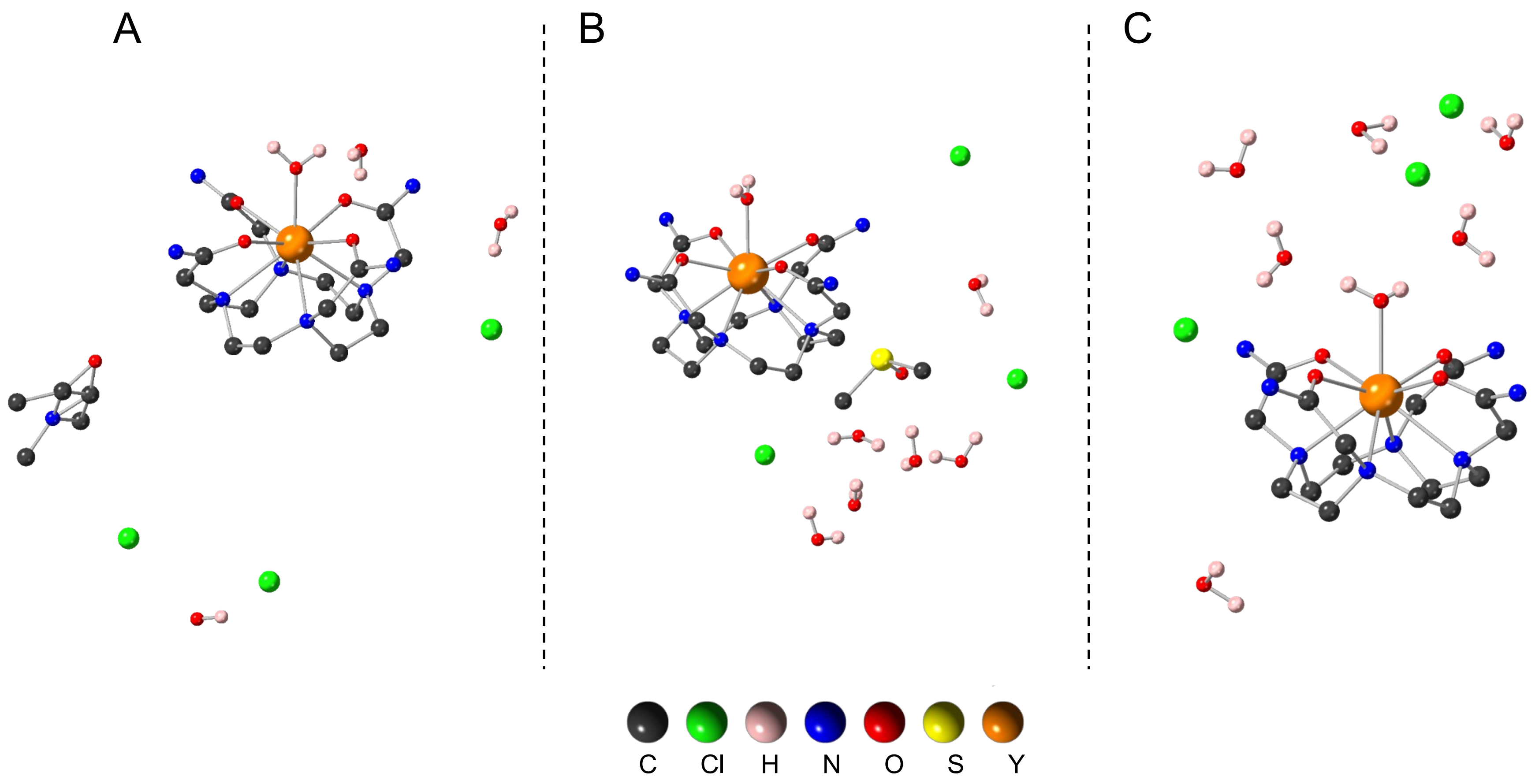

Figure S7: Asymmetric units of A) YDOTAMDMF, B) YDOTAMDMSO, and C) YDOTAMDMA

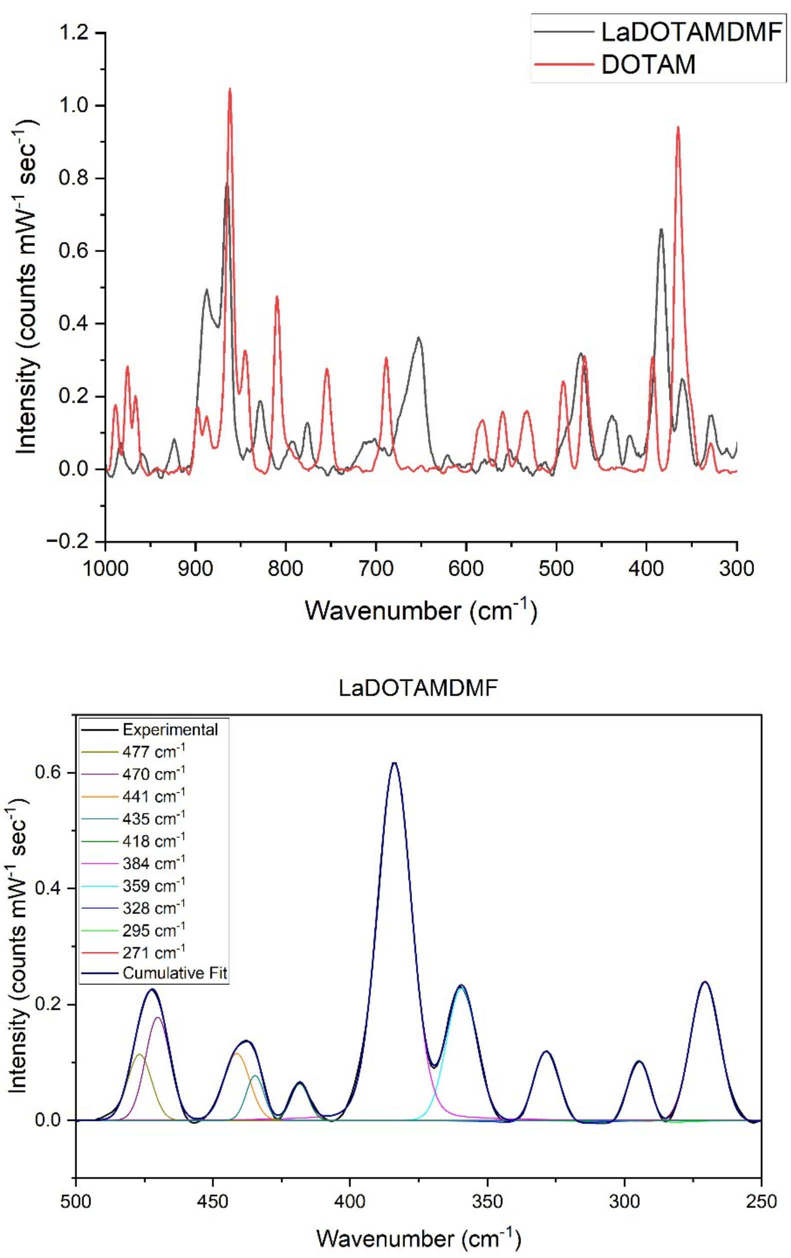

Figure S8: Raman overlay of DOTAM and LaDOTAMDMF spectra for comparison Fitted Raman and fitting statistics of LaDOTAMDMF in the spectral region of interest (550-250 cm^{-1}) with a R^2 of 0.9991 and reduced $\chi 2$ of 1.6 x 10^{-5}.

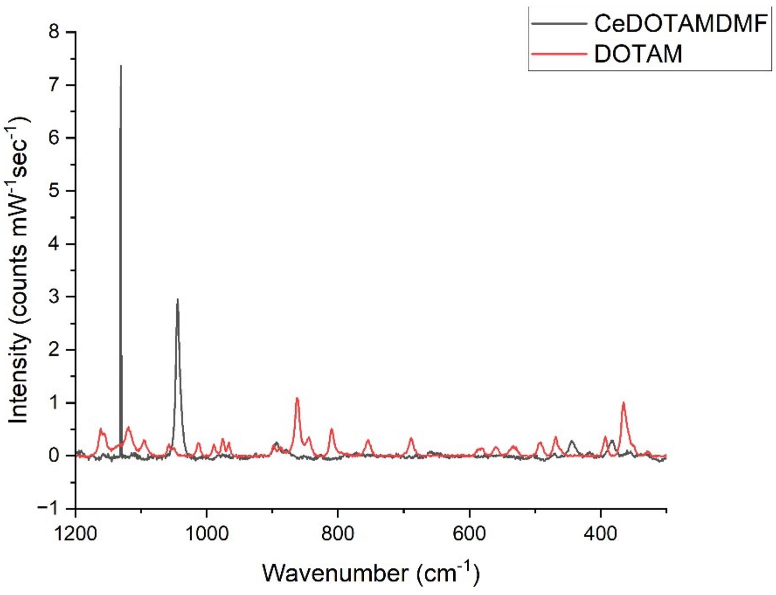

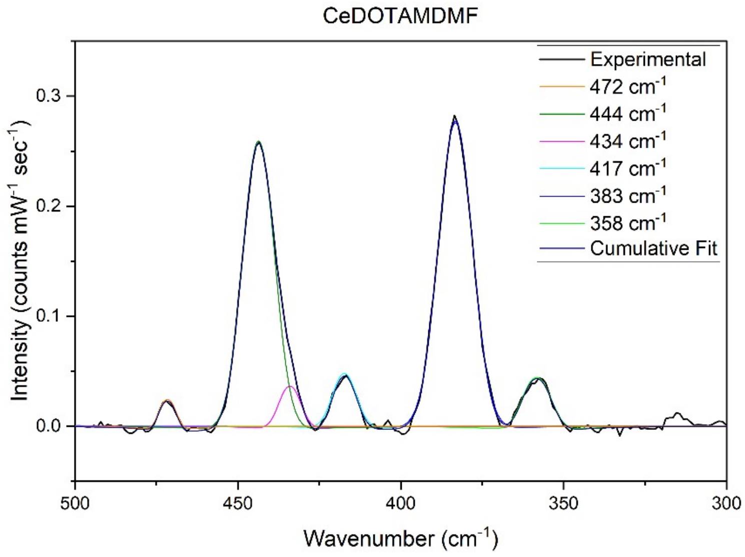

Figure S9: Raman overlay of DOTAM and CeDOTAMDMF spectra for comparison Fitted Raman and fitting statistics of CeDOTAMDMF in the spectral region of interest (500-300 cm^{-1}) with a R^2 of 0.9981 and reduced $\chi 2$ of 1.1 x 10^{-5}.

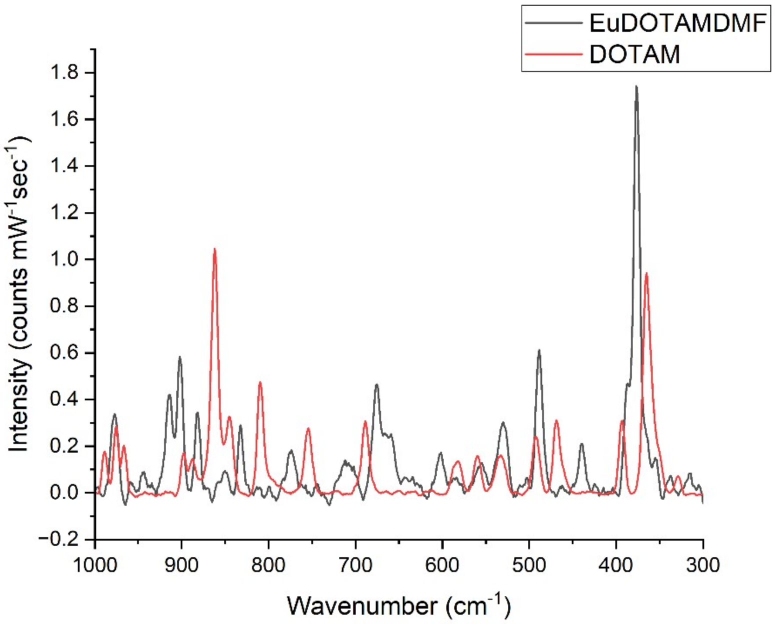

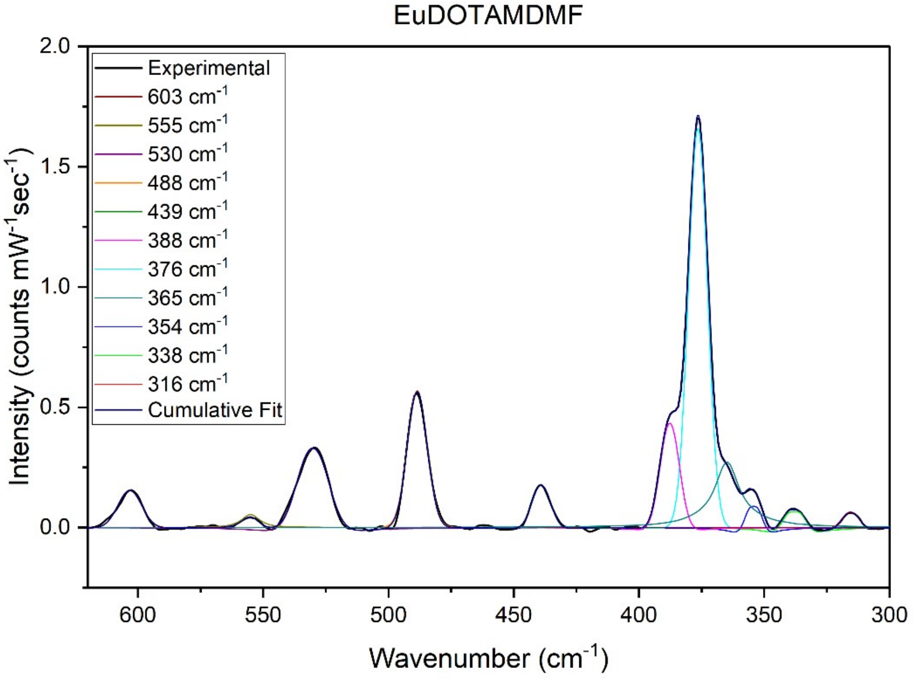

Figure S10: Raman overlay of DOTAM and EuDOTAMDMF spectra for comparison Fitted Raman and fitting statistics of EuDOTAMDMF in the spectral region of interest (620-300 cm^{-1}) with a R_2 of 0.9993 and reduced $\chi 2$ of 6.0 x 10^{-5}.

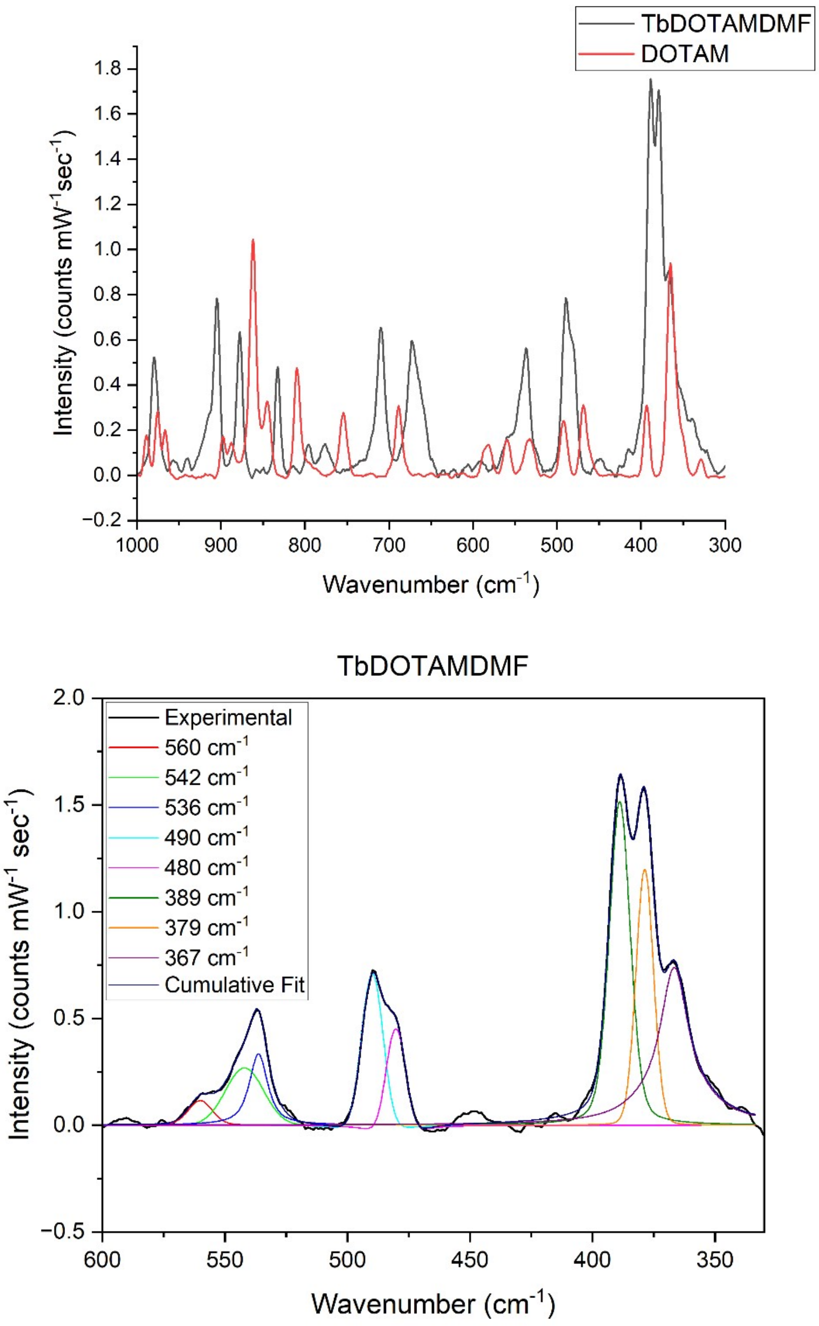

Figure S11: Raman overlay of DOTAM and TbDOTAMDMF spectra for comparison Fitted Raman and fitting statistics of TbDOTAMDMF in the spectral region of interest (600-300 cm^{-1}) with a R^2 of 0.9979 and reduced $\chi 2$ of 3.7 x 10^{-4}.

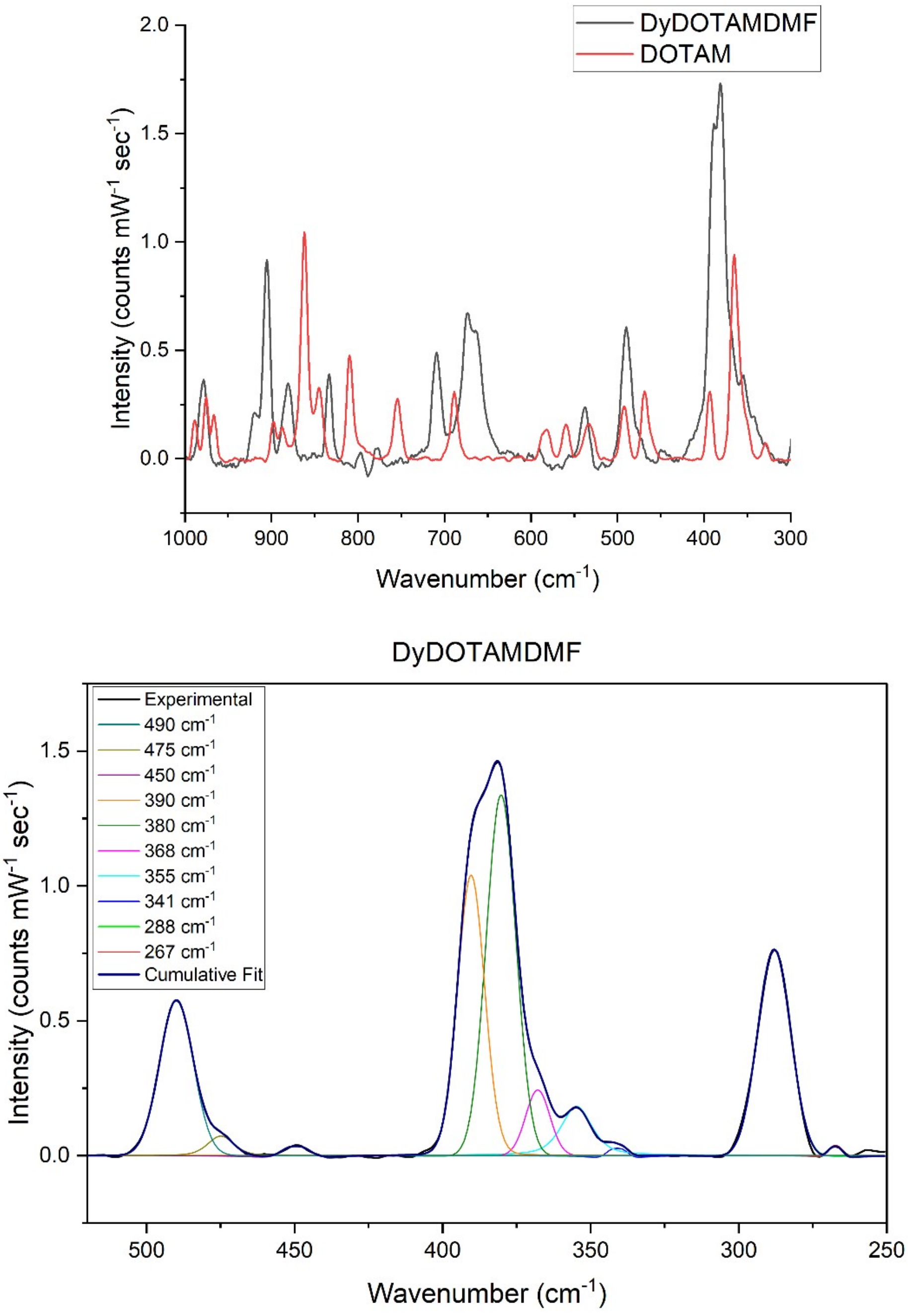

Figure S12: Raman overlay of DOTAM and DyDOTAMDMF spectra for comparison Fitted Raman and fitting statistics of DyDOTAMDMF in the spectral region of interest (520-250 cm^{-1}) with a R^2 of 0.9997 and reduced $\chi2$ of 4.9 x 10^{-5}.

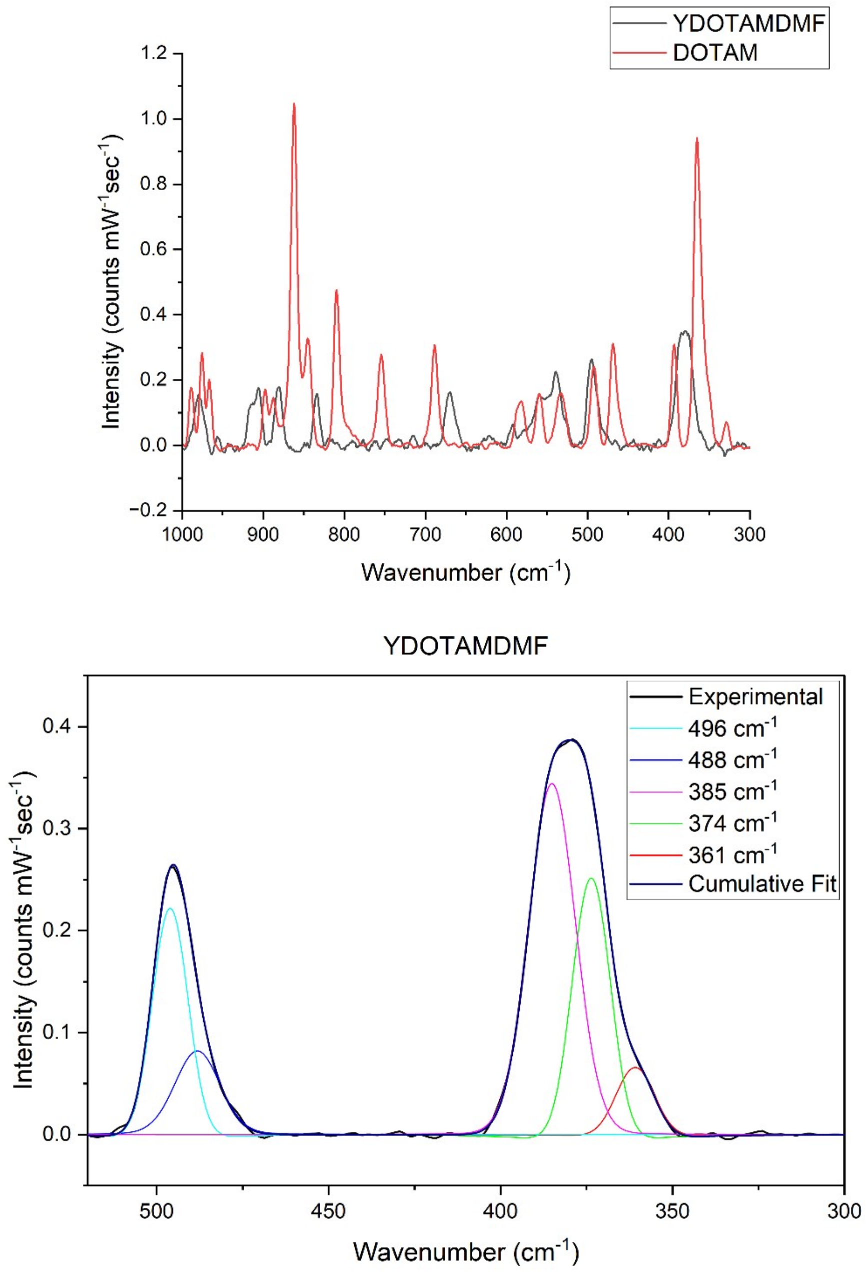

Figure S13: Raman overlay of DOTAM and YDOTAMDMF spectra for comparison Fitted Raman and fitting statistics of YDOTAMDMF in the spectral region of interest (520-300 cm^{-1}) with a R^2 of 0.9997 and reduced $\chi 2$ of 4.1 x 10^{-6}.

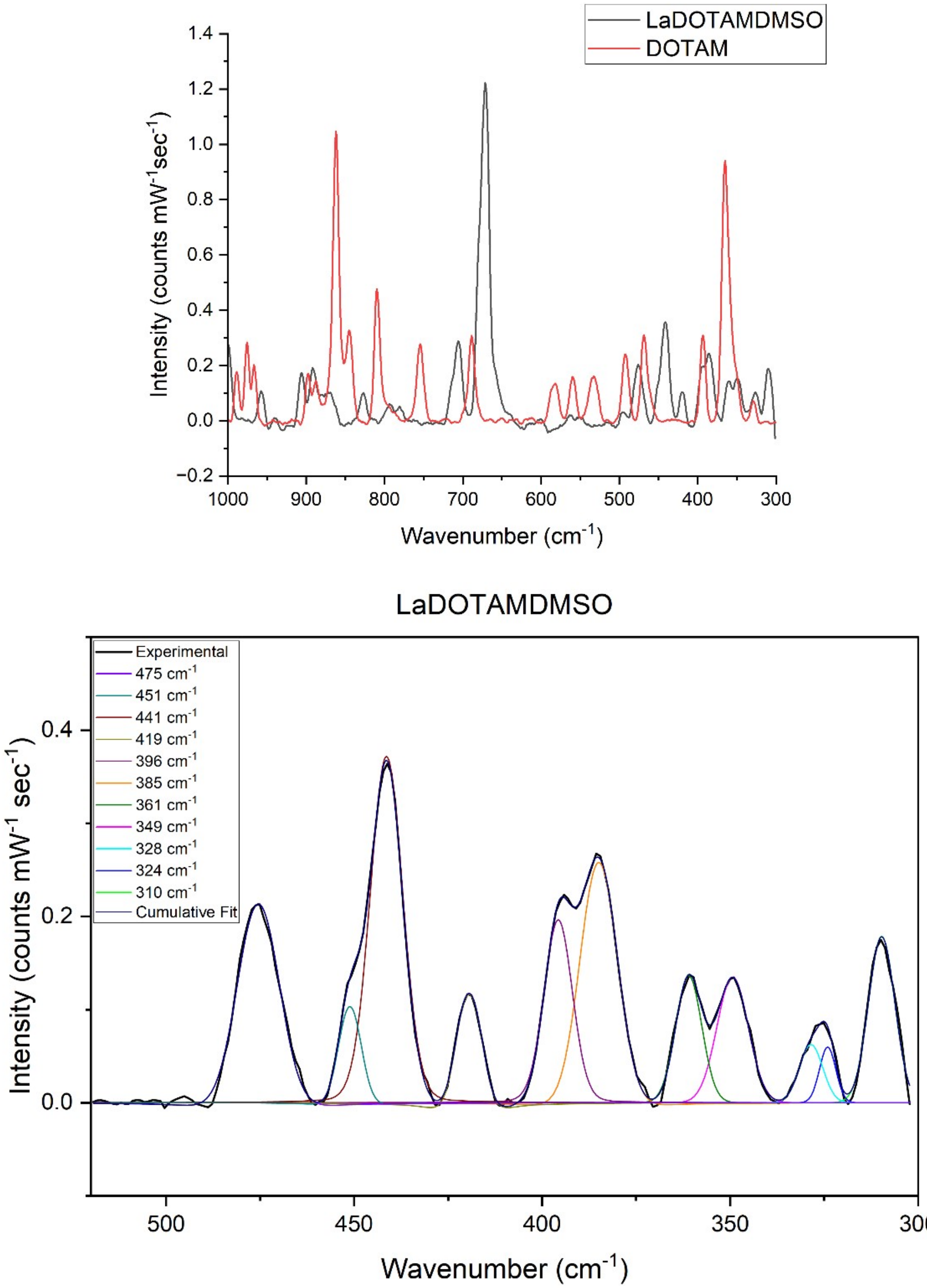

Figure S14: Raman overlay of DOTAM and LaDOTAMDMSO spectra for comparison Fitted Raman and fitting statistics of LaDOTAMDMSO in the spectral region of interest (520-300 cm^{-1}) with a R^2 of 0.9971 and reduced $\chi 2$ of 2.7×10^{-5}.

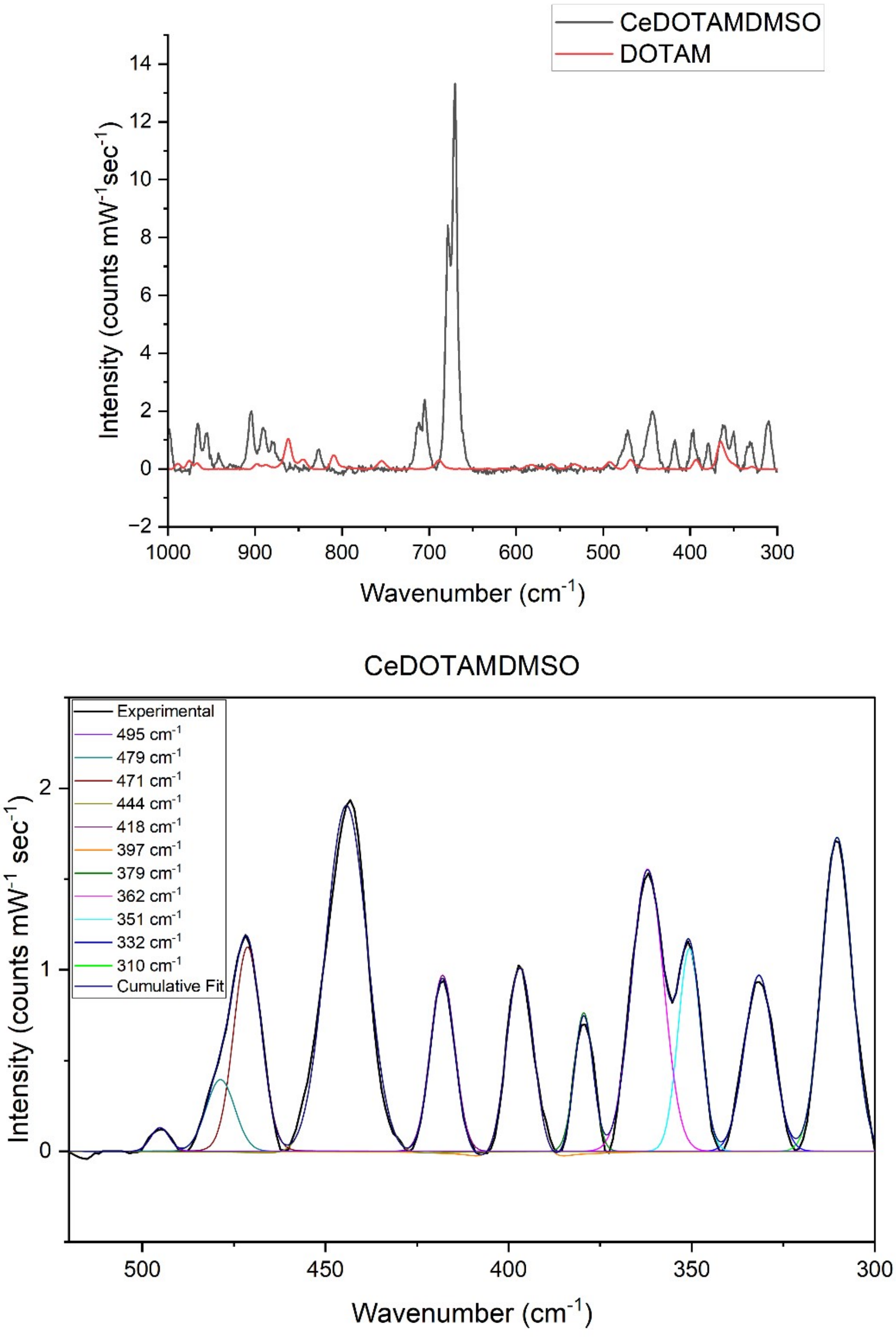

Figure S15: Raman overlay of DOTAM and CeDOTAMDMSO spectra for comparison Fitted Raman and fitting statistics of CeDOTAMDMSO in the spectral region of interest (520-300 cm^{-1}) with a R^2 of 0.9947 and reduced $\chi 2$ of 1.7×10^{-3}.

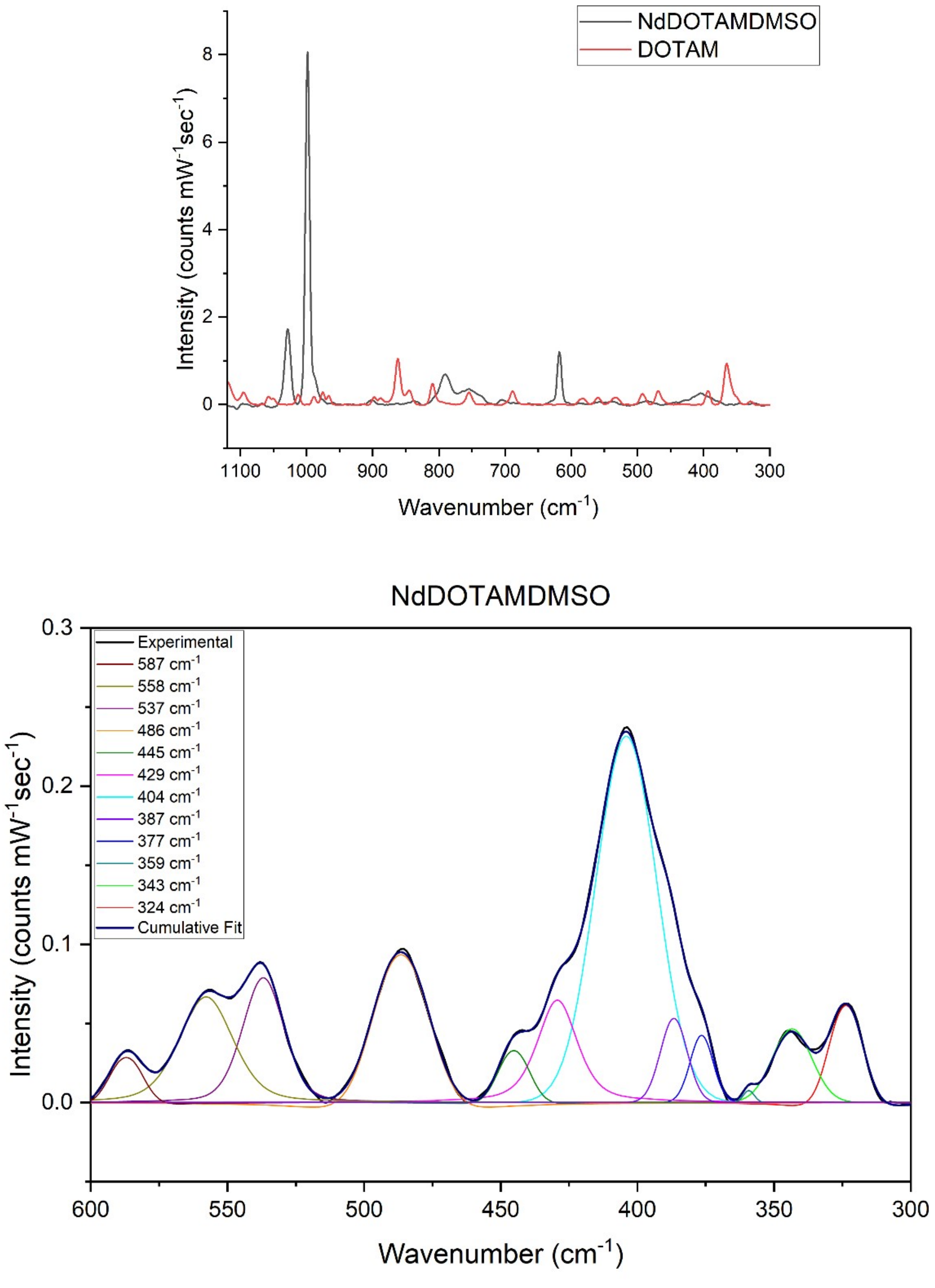

Figure S16: Raman overlay of DOTAM and NdDOTAMDMSO spectra for comparison Fitted Raman and fitting statistics of NdDOTAMDMSO in the spectral region of interest (600-300 cm^{-1}) with a R^2 of 0.9994 and reduced $\chi 2$ of 2.0×10^{-6}.

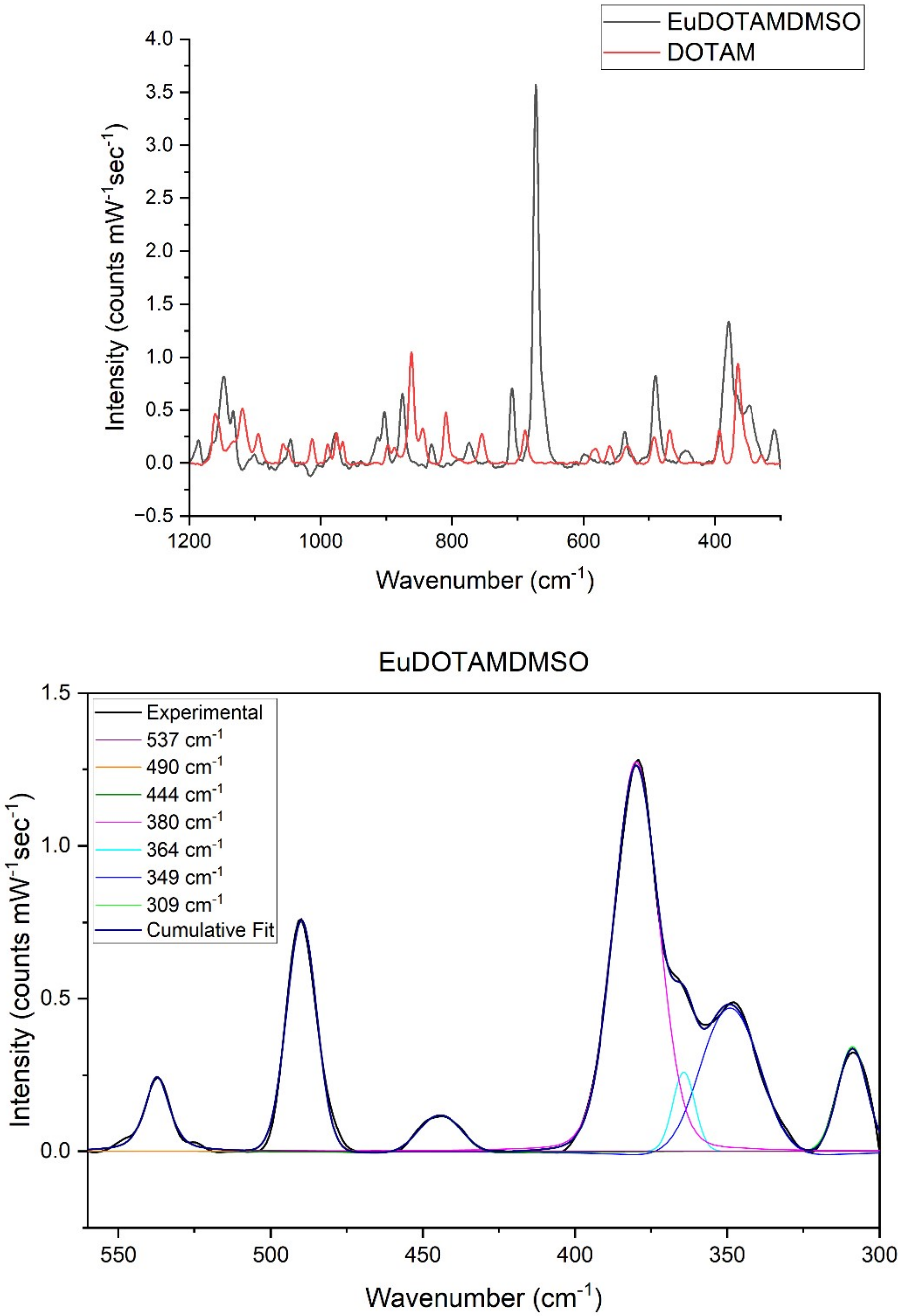

Figure S17: Raman overlay of DOTAM and EuDOTAMDMSO spectra for comparison Fitted Raman and fitting statistics of EuDOTAMDMSO in the spectral region of interest (520-250 cm^{-1}) with a R^2 of 0.9982 and reduced $\chi 2$ of 1.7 x 10^{-4}.

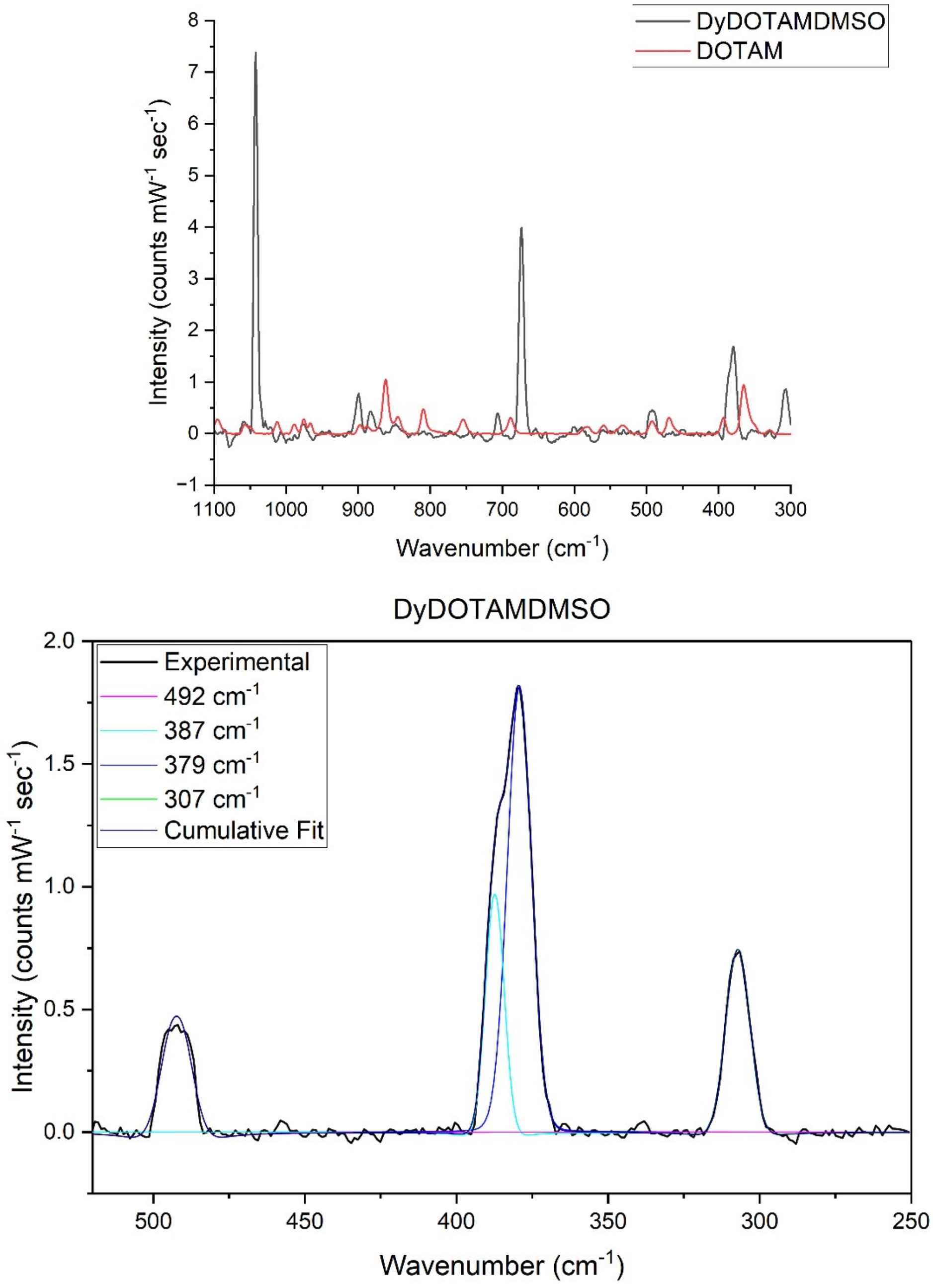

Figure S18: Raman overlay of DOTAM and DyDOTAMDMSO spectra for comparison Fitted Raman and fitting statistics of DyDOTAMDMSO in the spectral region of interest (520-250 cm^{-1}) with a R^2 of 0.9965 and reduced $\chi 2$ of 4.7 x 10^{-4}.

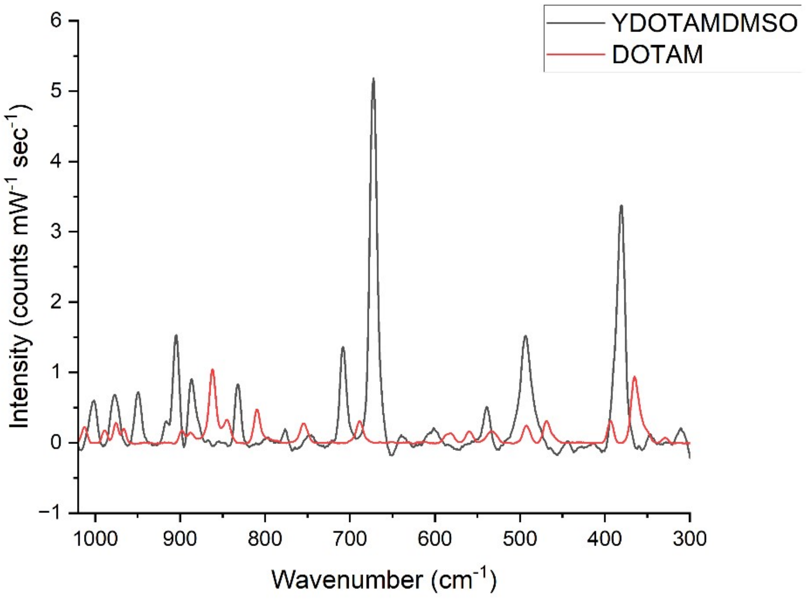

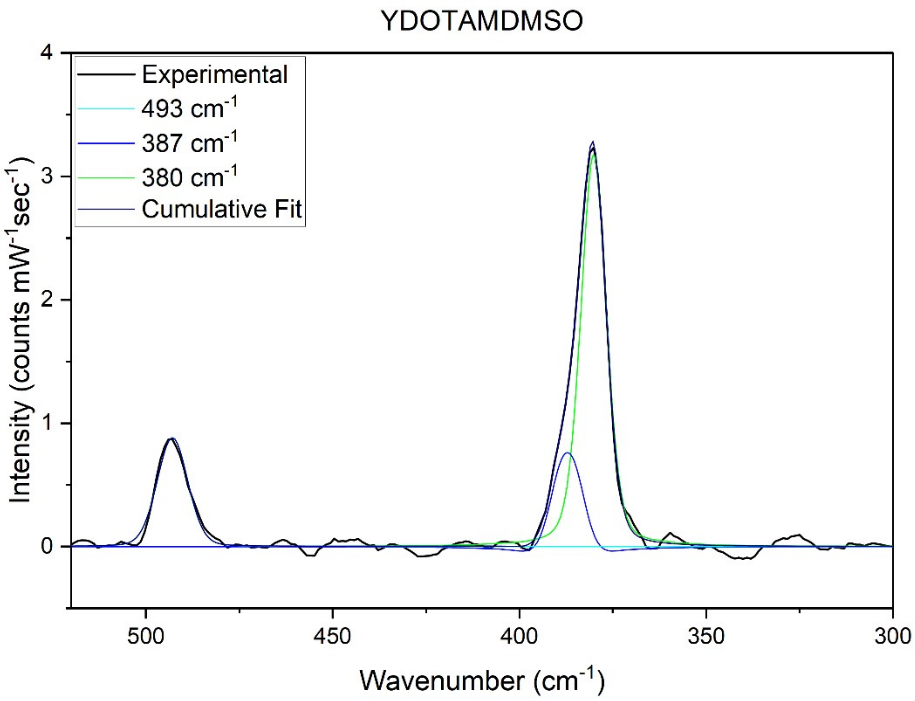

Figure S19: Raman overlay of DOTAM and YDOTAMDMSO spectra for comparison Fitted Raman and fitting statistics of YDOTAMDMSO in the spectral region of interest (520-300 cm^{-1}) with a R^2 of 0.9949 and reduced $\chi 2$ of 1.8 x 10^{-3}.

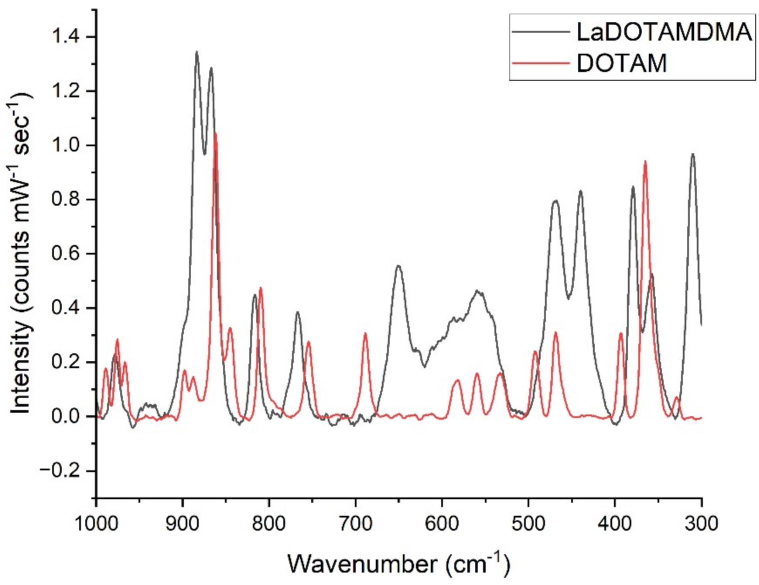

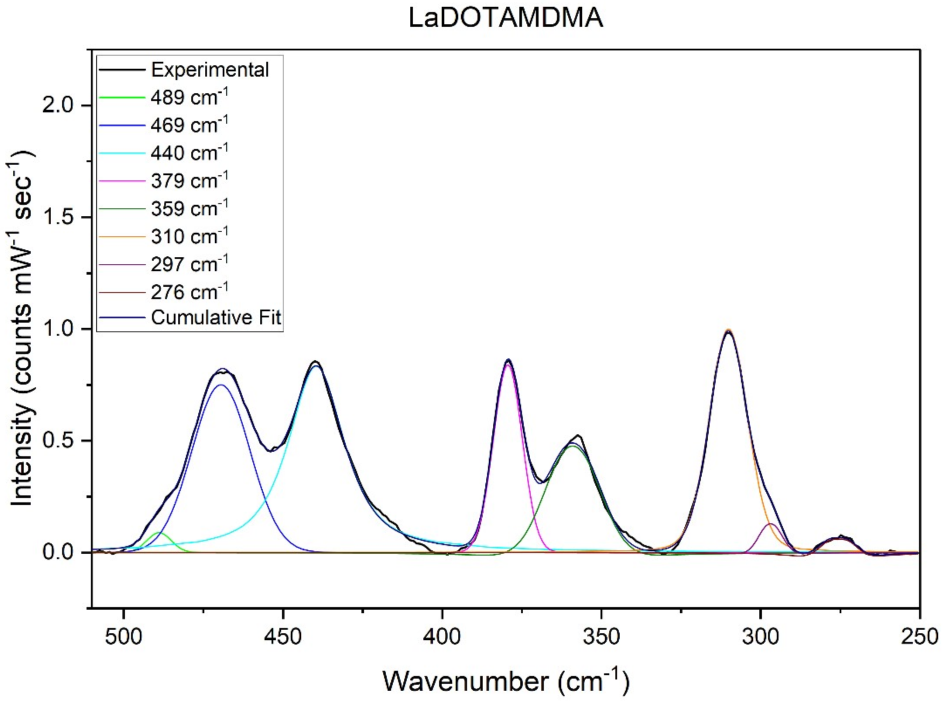

Figure S20: Raman overlay of DOTAM and LaDOTAMDMA spectra for comparison Fitted Raman and fitting statistics of LaDOTAMDMA in the spectral region of interest (510-250 cm^{-1}) with a R^2 of 0.9971 and reduced $\chi 2$ of 2.8 x 10^{-4}.

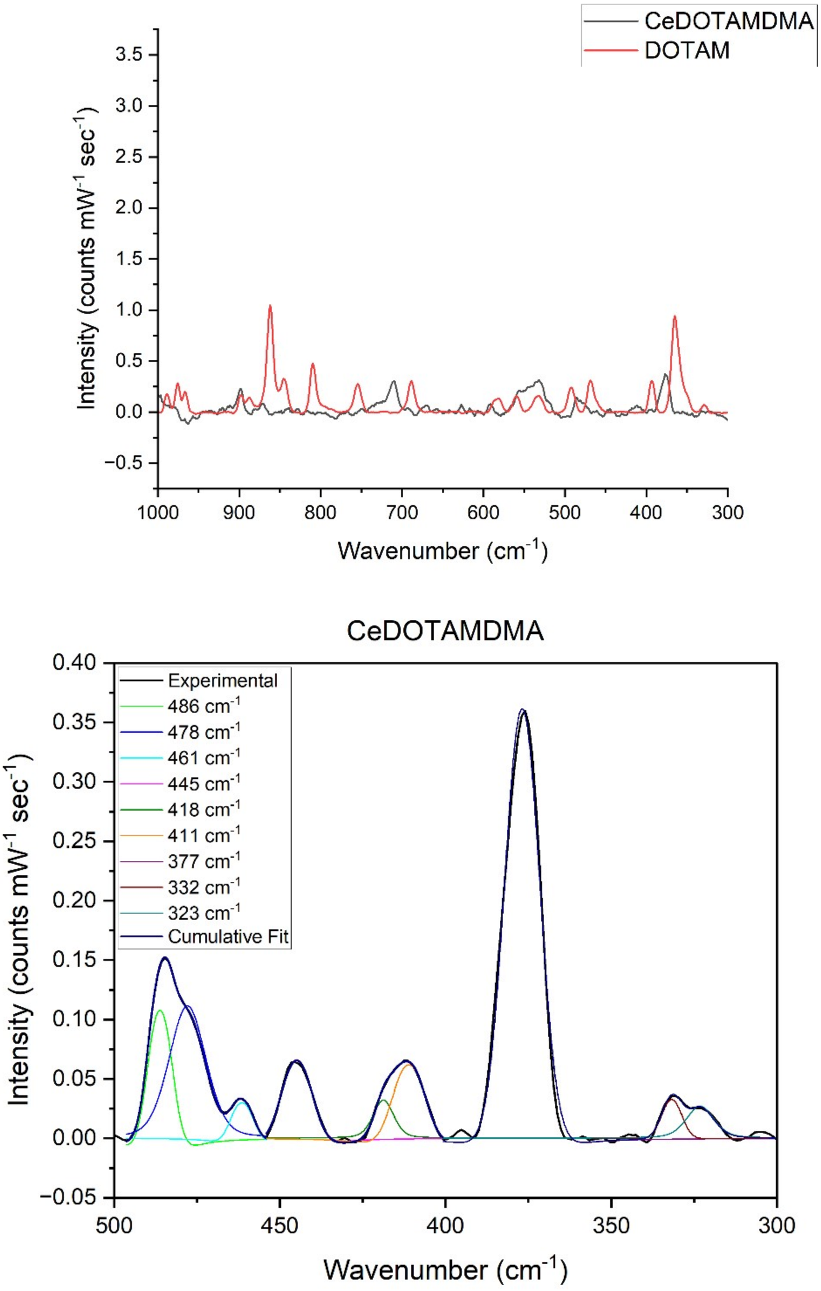

Figure S21: Raman overlay of DOTAM and CeDOTAMDMA spectra for comparison Fitted Raman and fitting statistics of CeDOTAMDMA in the spectral region of interest (500-300 cm^{-1}) with a R^2 of 0.9952 and reduced $\chi 2$ of 5.3 x 10^{-3}.

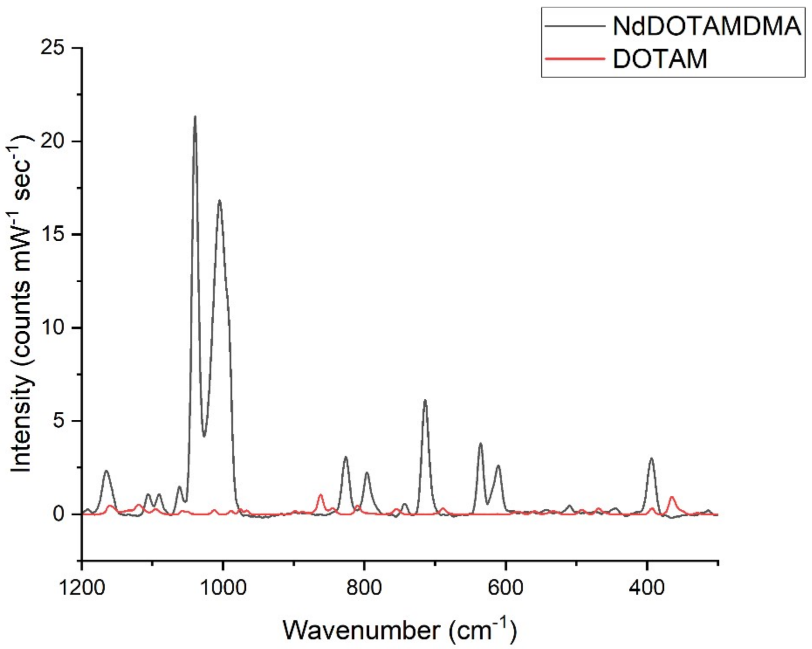

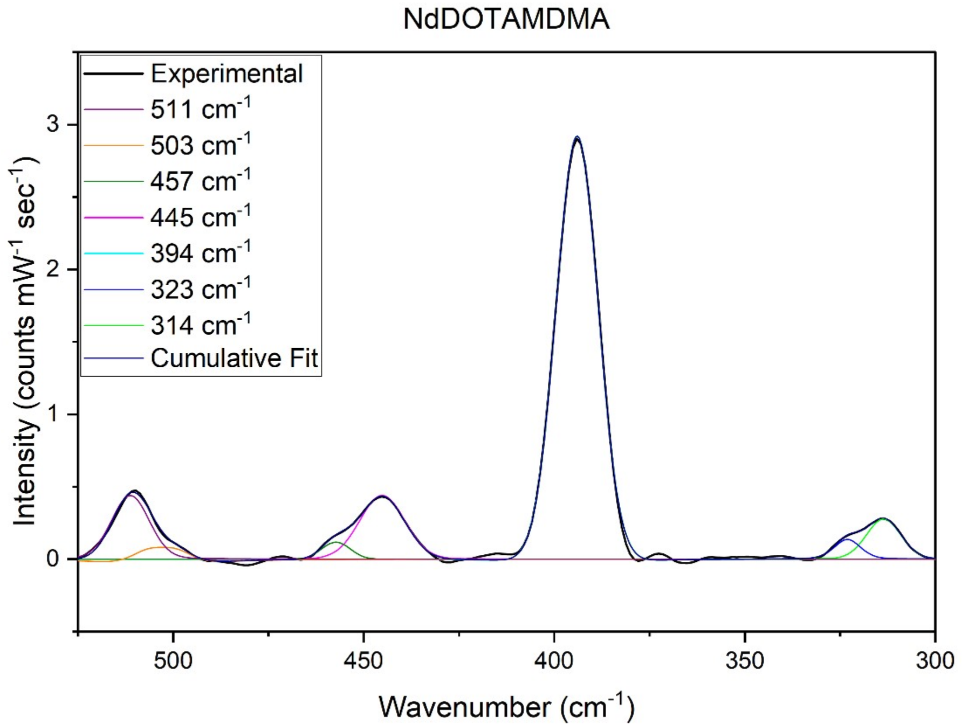

Figure S22: Raman overlay of DOTAM and NdDOTAMDMA spectra for comparison Fitted Raman and fitting statistics of NdDOTAMDMA in the spectral region of interest (525-300 cm^{-1}) with a R^2 of 0.9990 and reduced $\chi 2$ of 3.5 x 10^{-4}.

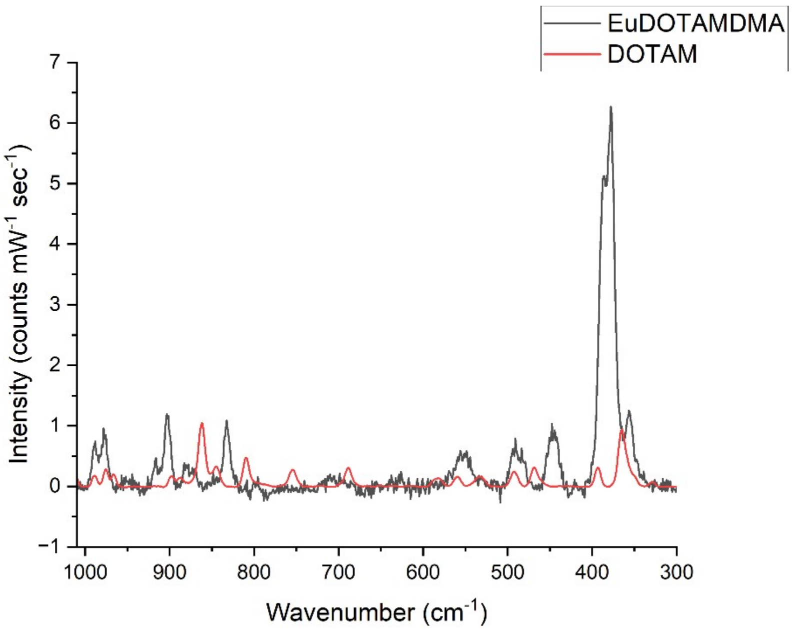

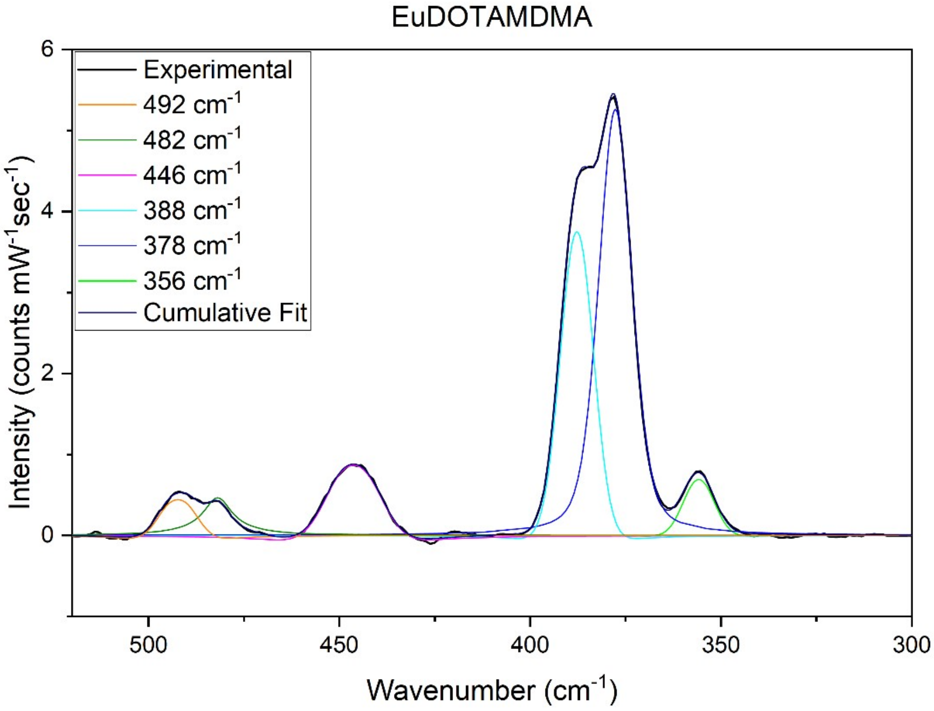

Figure S23: Raman overlay of DOTAM and EuDOTAMDMA spectra for comparison Fitted Raman and fitting statistics of EuDOTAMDMA in the spectral region of interest (520-300 cm^{-1}) with a R^2 of 0.9997 and reduced $\chi 2$ of 5.6 x 10^{-4}.

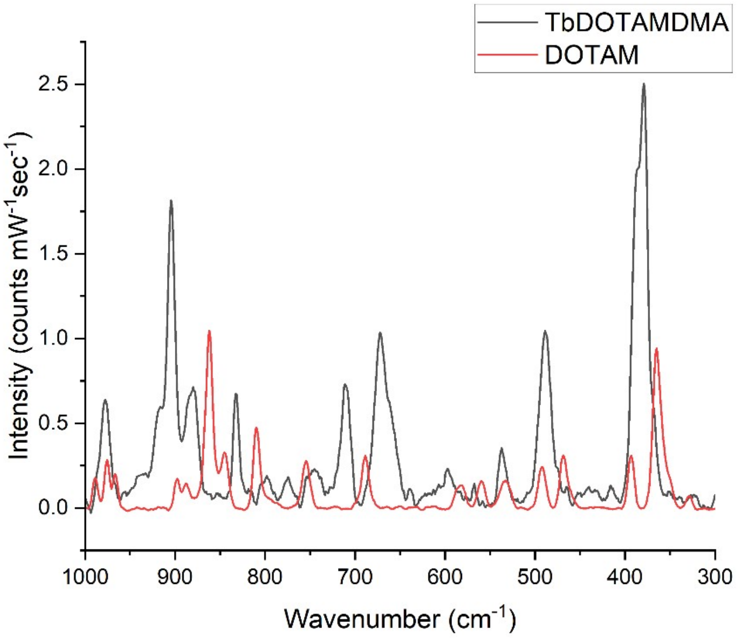

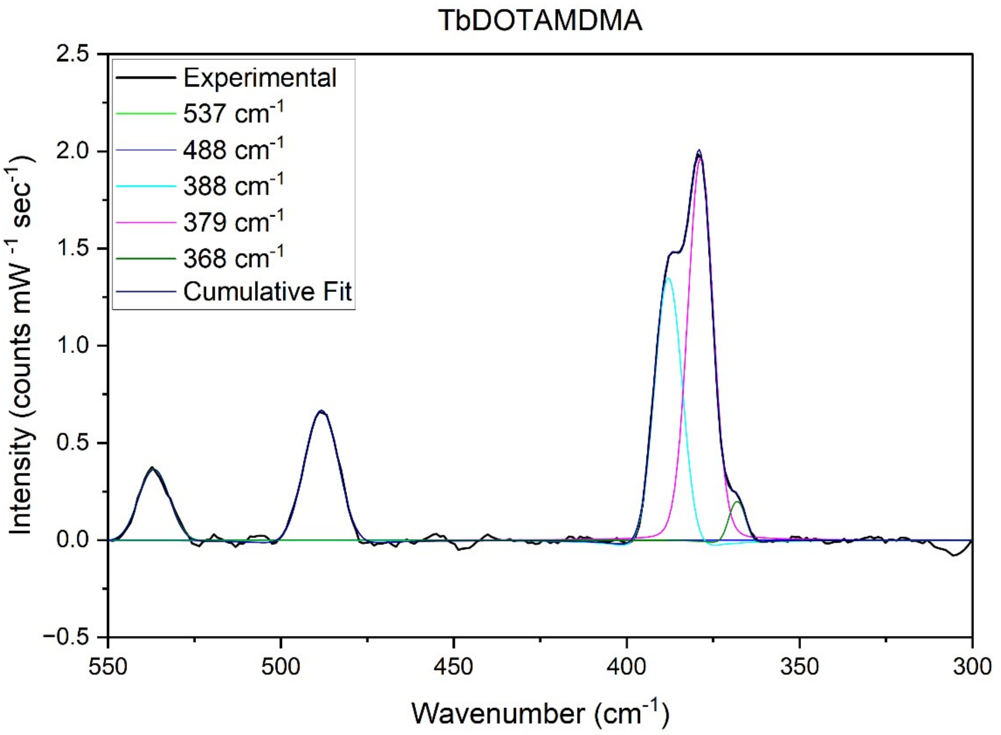

Figure S24: Raman overlay of DOTAM and TbDOTAMDMA spectra for comparison Fitted Raman and fitting statistics of TbDOTAMDMA in the spectral region of interest (550-300 cm^{-1}) with a R^2 of 0.9979 and reduced $\chi 2$ of 3.8 x 10^{-4}.

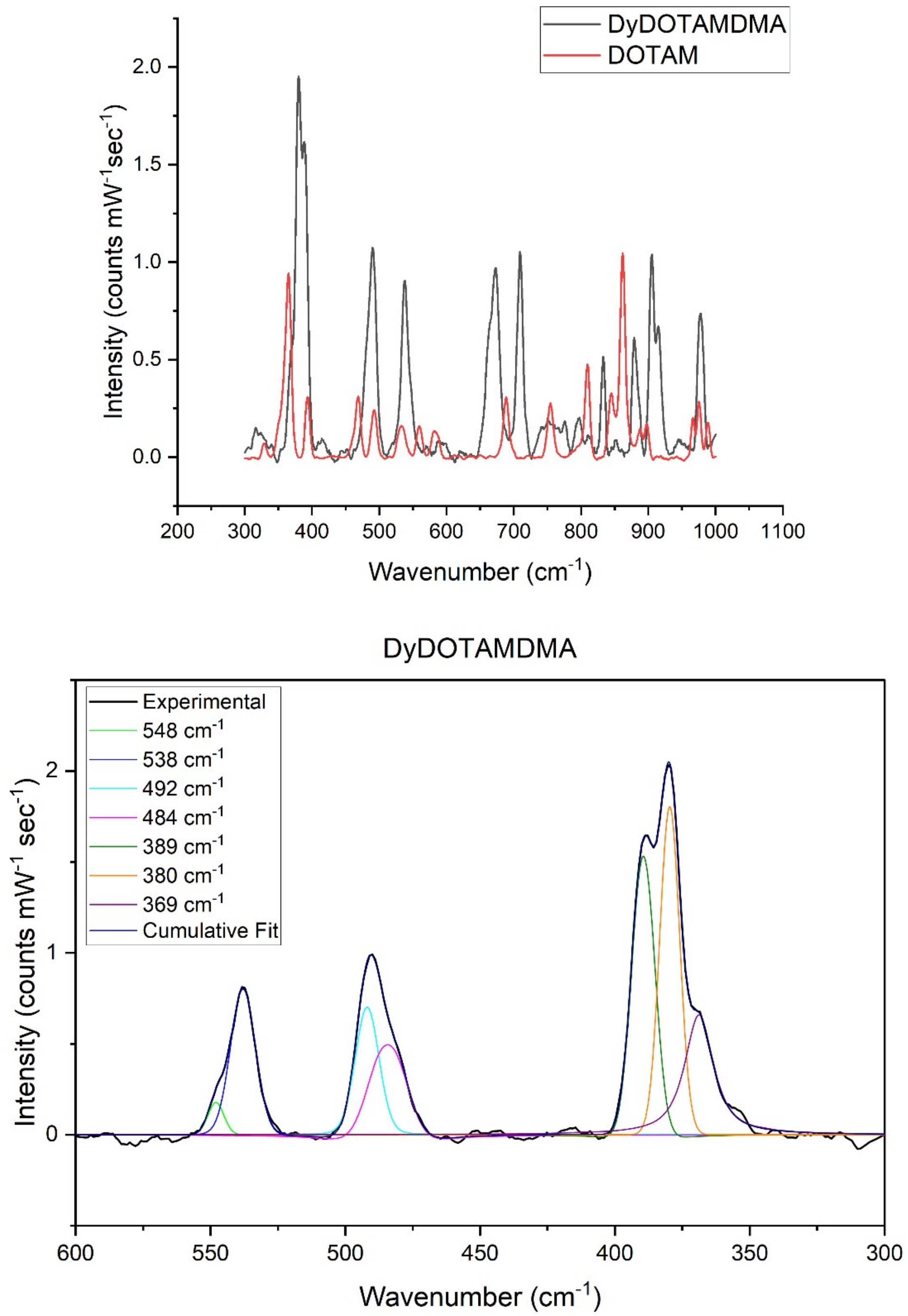

Figure S25: Raman overlay of DOTAM and DyDOTAMDMA spectra for comparison Fitted Raman and fitting statistics of DyDOTAMDMA in the spectral region of interest (600-300 cm^{-1}) with a R^2 of 0.9978 and reduced $\chi 2$ of 4.8 x 10^{-4}.

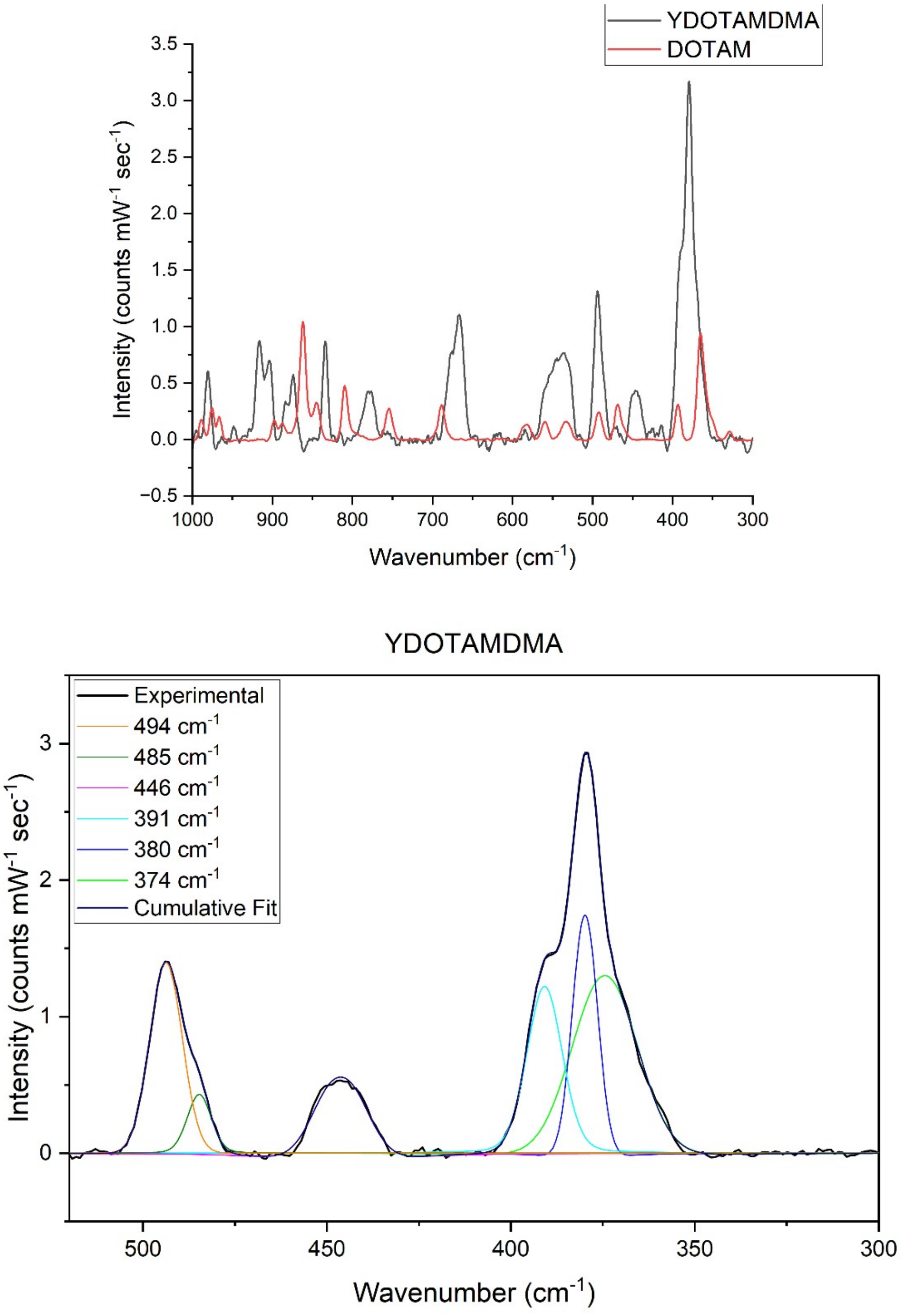

Figure S26: Raman overlay of DOTAM and YDOTAMDMA spectra for comparison Fitted Raman and fitting statistics of YDOTAMDMA in the spectral region of interest (520-300 cm^{-1}) with a R^2 of 0.9988 and reduced $\chi 2$ of 5.6 x 10^{-4}.

Table S7: The average and maximum/minimum twist angle of 9- and 10-coordinate Ln DOTAM complexes, respectively. Square anti-prism, twisted square antiprism, and distorted geometries are highlighted in pink, green, and purple, respectively. Angles were found using the angle tool in CrystalMaker 11.

9 coordinate complex twist angles

DMF/H_2O

	Europium	Terbium	Dysprosium	Yttrium
Average twist angle (°)	38.6 (2.0)	38.8 (4.8)	39.2 (4.4)	39.1 (1.6)

DMSO/H_2O

	Neodymium	Europium	Dysprosium	Yttrium
Average twist angle (°)	23.8 (4.4)	38.5 (1.5)	39.0 (3.5)	39.7 (1.7)

DMA/H_2O

	Cerium	Neodymium	Europium	Terbium	Dysprosium	Yttrium
Average twist angle (°)	22.9 (4.4)	38.8 (3.5)	38.8 (1.0)	38.7 (5.7)	39.4 (4.2)	39.4 (1.5)

10 coordinate complex twist angles

	LaDMF	CeDMF	LaDMSO	CeDMSO	LaDMA
Maximum twist angle (°)	38.3	37.3	37.1	36.1	33.1
Minimum twist angle (°)	6.4	7.3	3.0	9.7	9.4

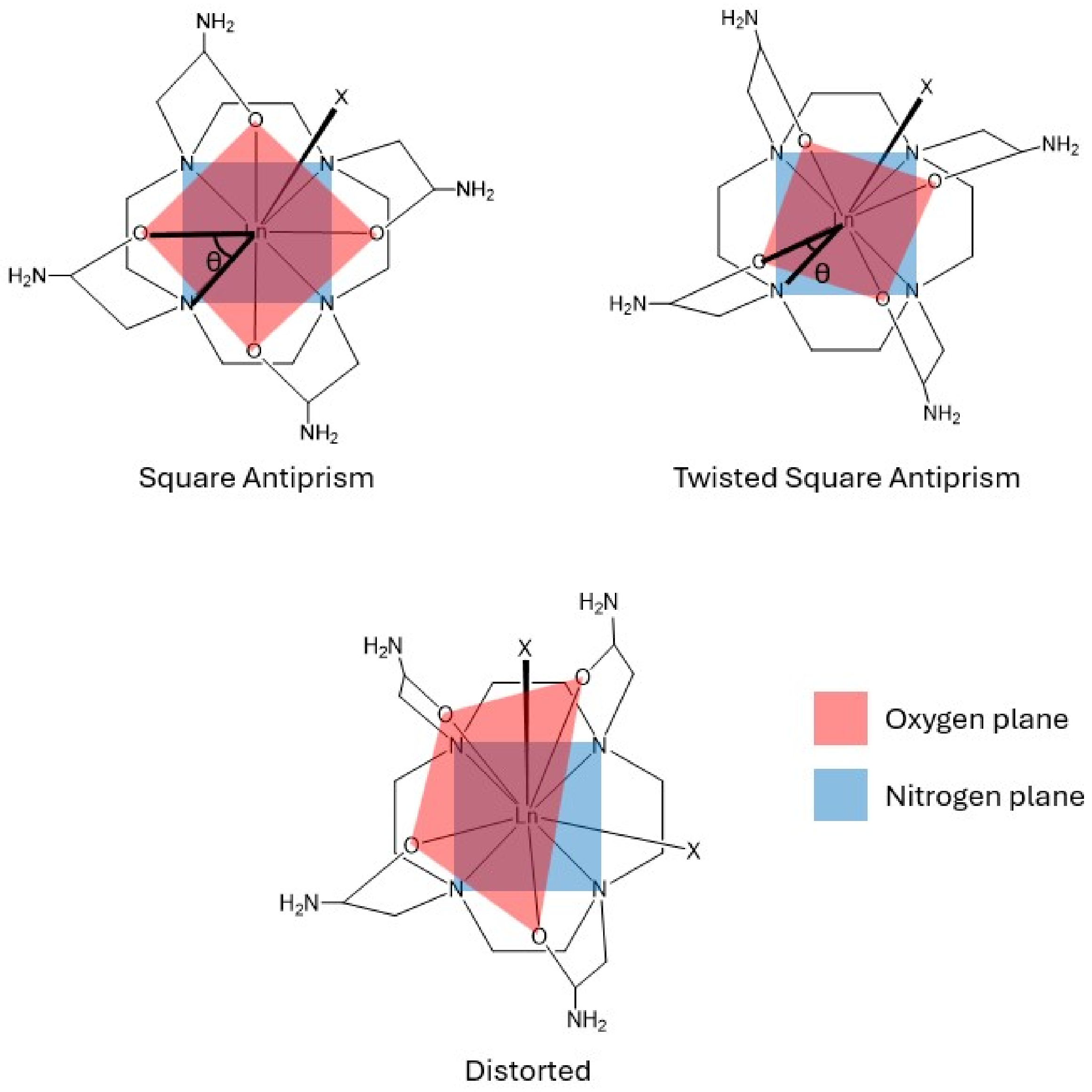

Figure S27: The twist angle of the complexes is determined by the angle of rotation between the oxygen and nitrogen planes. A twist angle closer to 45° corresponds to a square antiprism geometry while a smaller twist angle corresponds to a twisted square antiprism geometry**.**[26]

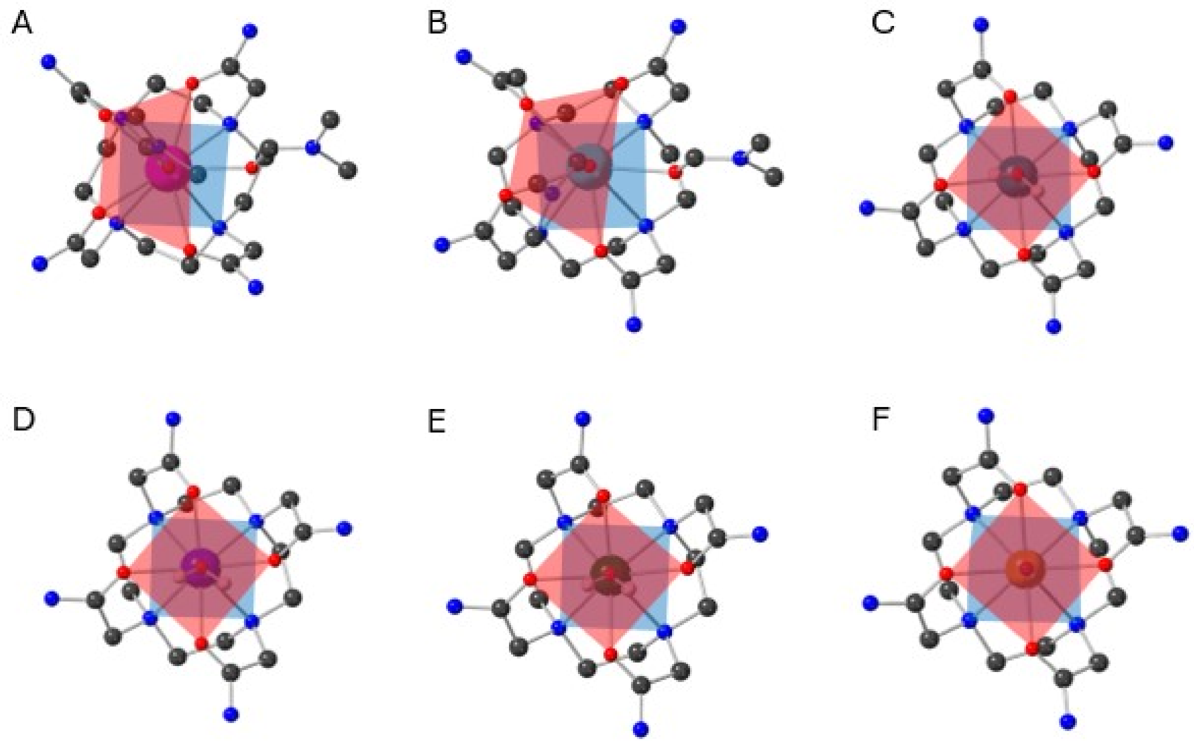

Figure S28: Top-down images of the six DMF complexes A) La, B) Ce, C) Eu, D) Tb, E) Dy, F) Y. Red boxes indicate the oxygen plane. Blue boxes indicate the nitrogen plane.

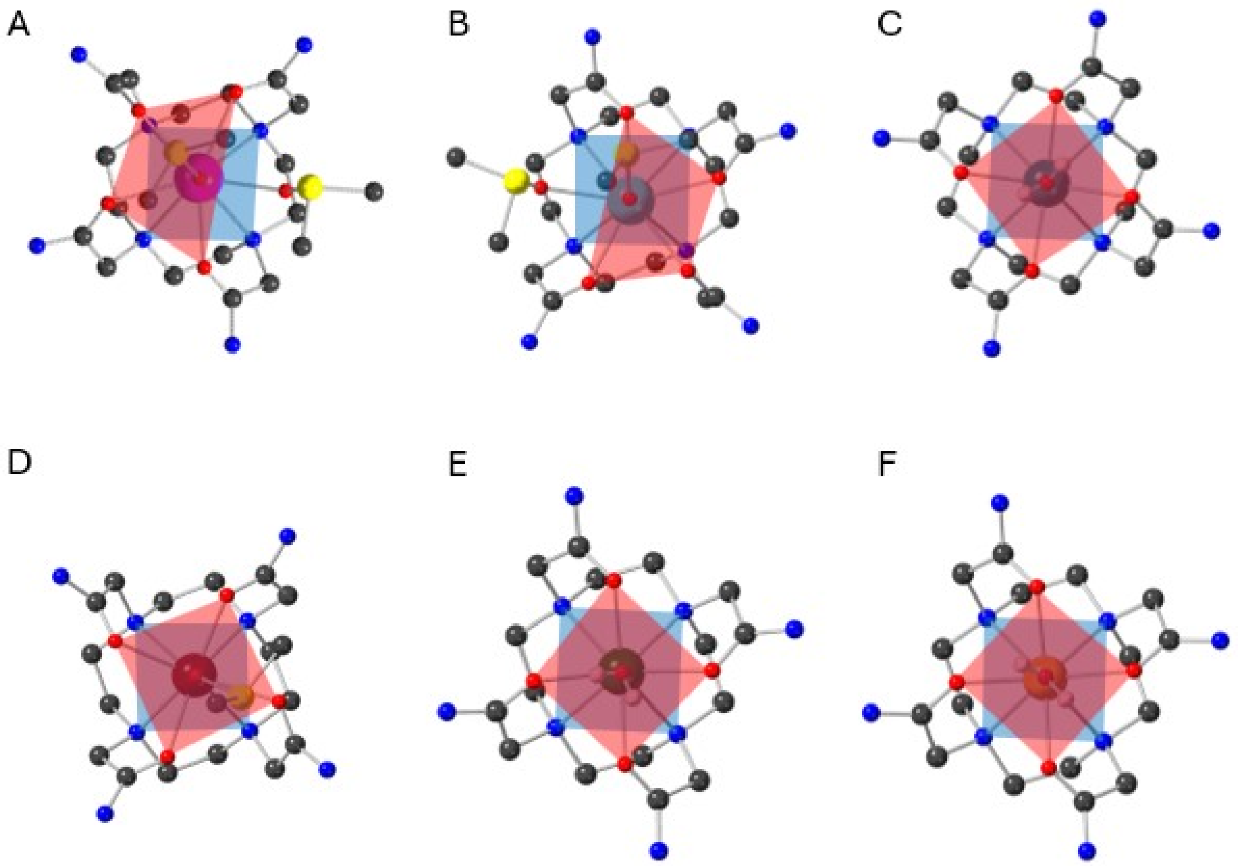

Figure S29: Top-down images of the six DMSO complexes A) La, B) Ce, C) Nd, D) Eu, E) Dy, F) Y. Red boxes indicate the oxygen plane. Blue boxes indicate the nitrogen plane.

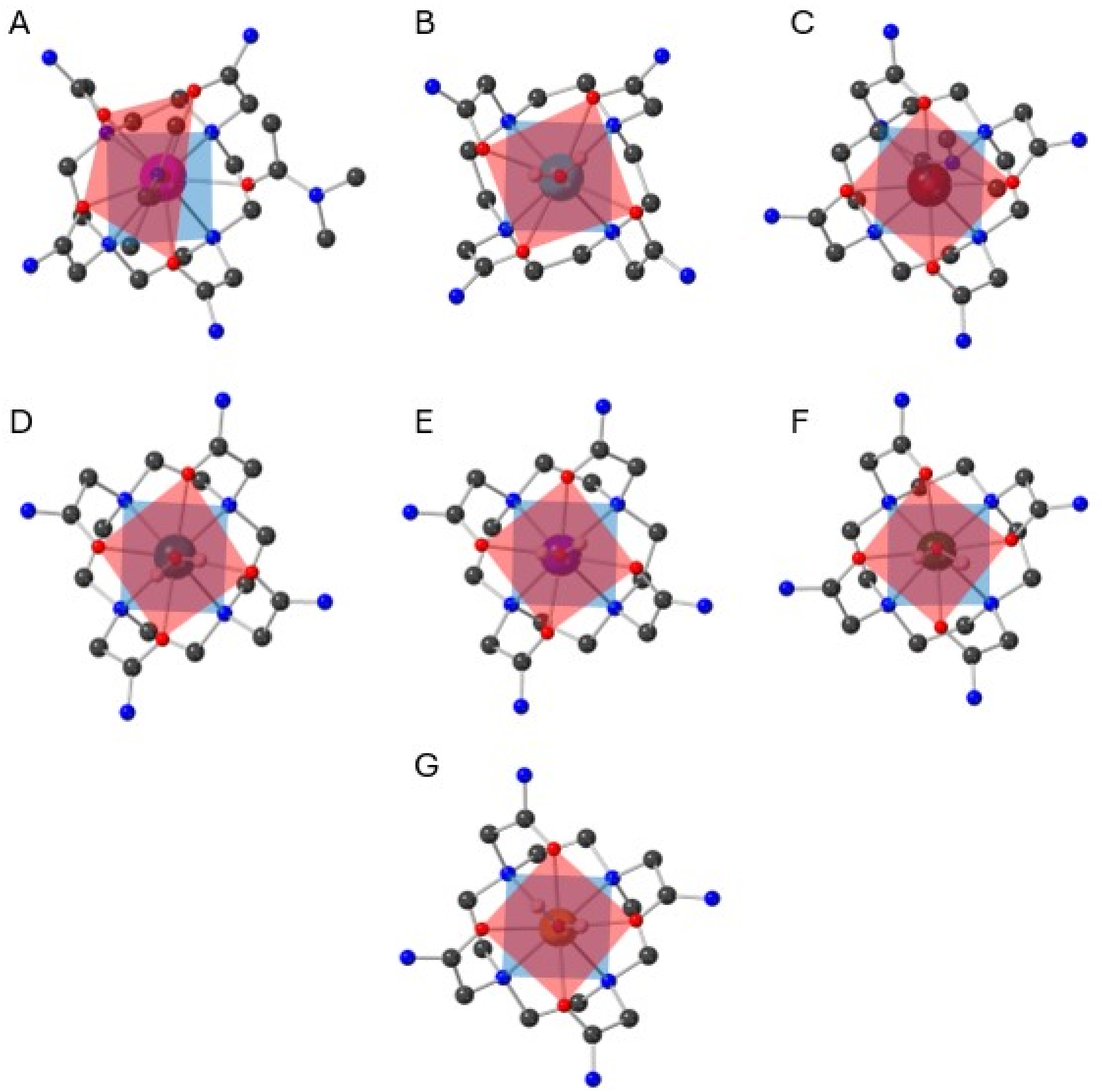

Figure S30: Top-down images of the seven DMA complexes A) La, B) Ce, C) Nd, D) Eu, E) Tb, F) Dy, G) Y. Red boxes indicate the oxygen plane. Blue boxes indicate the nitrogen plane.

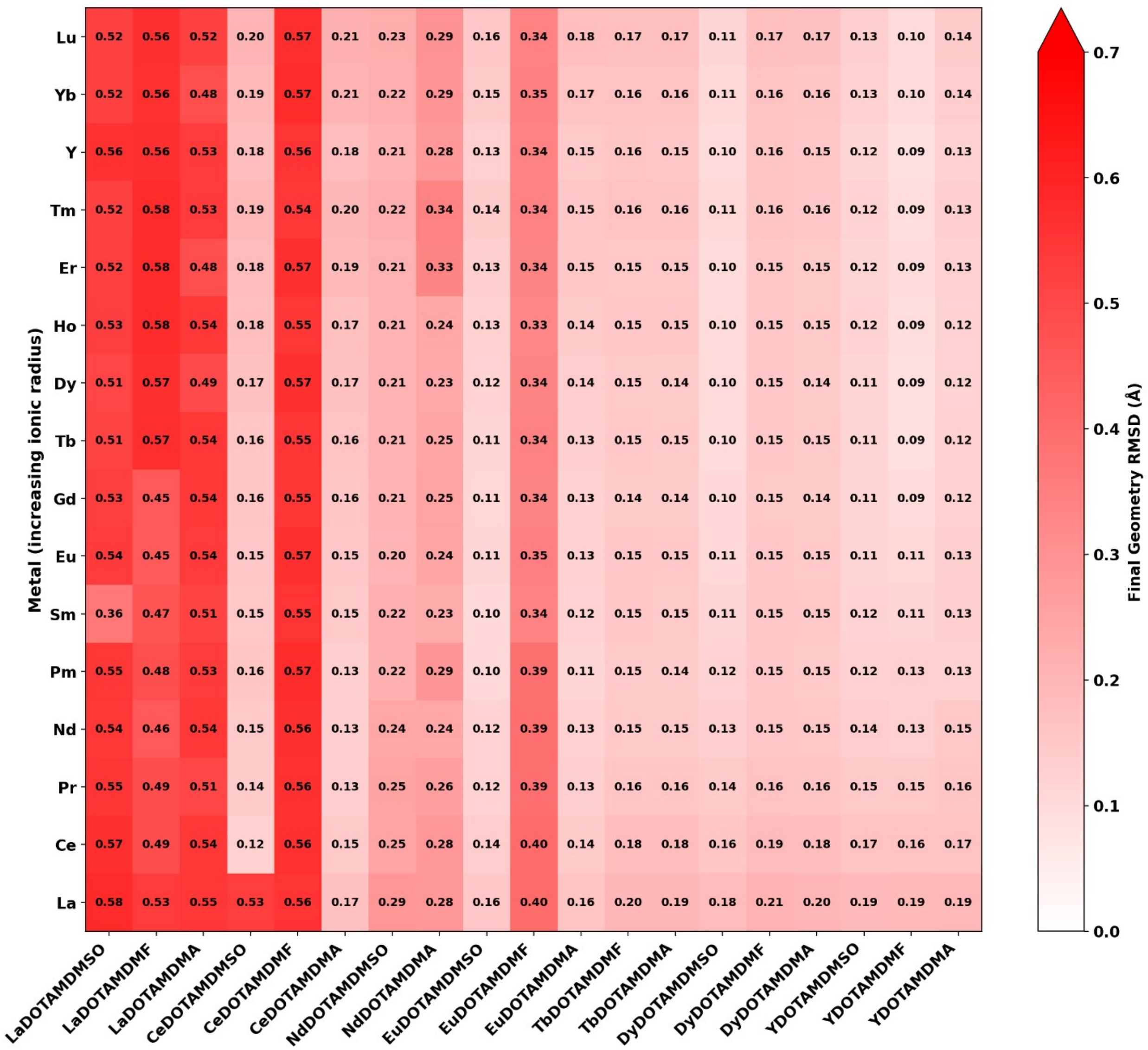

Figure S31: RMSD comparing the geometries of the scenario (a, *perfect template*) with scenario (b, *relaxed template*).

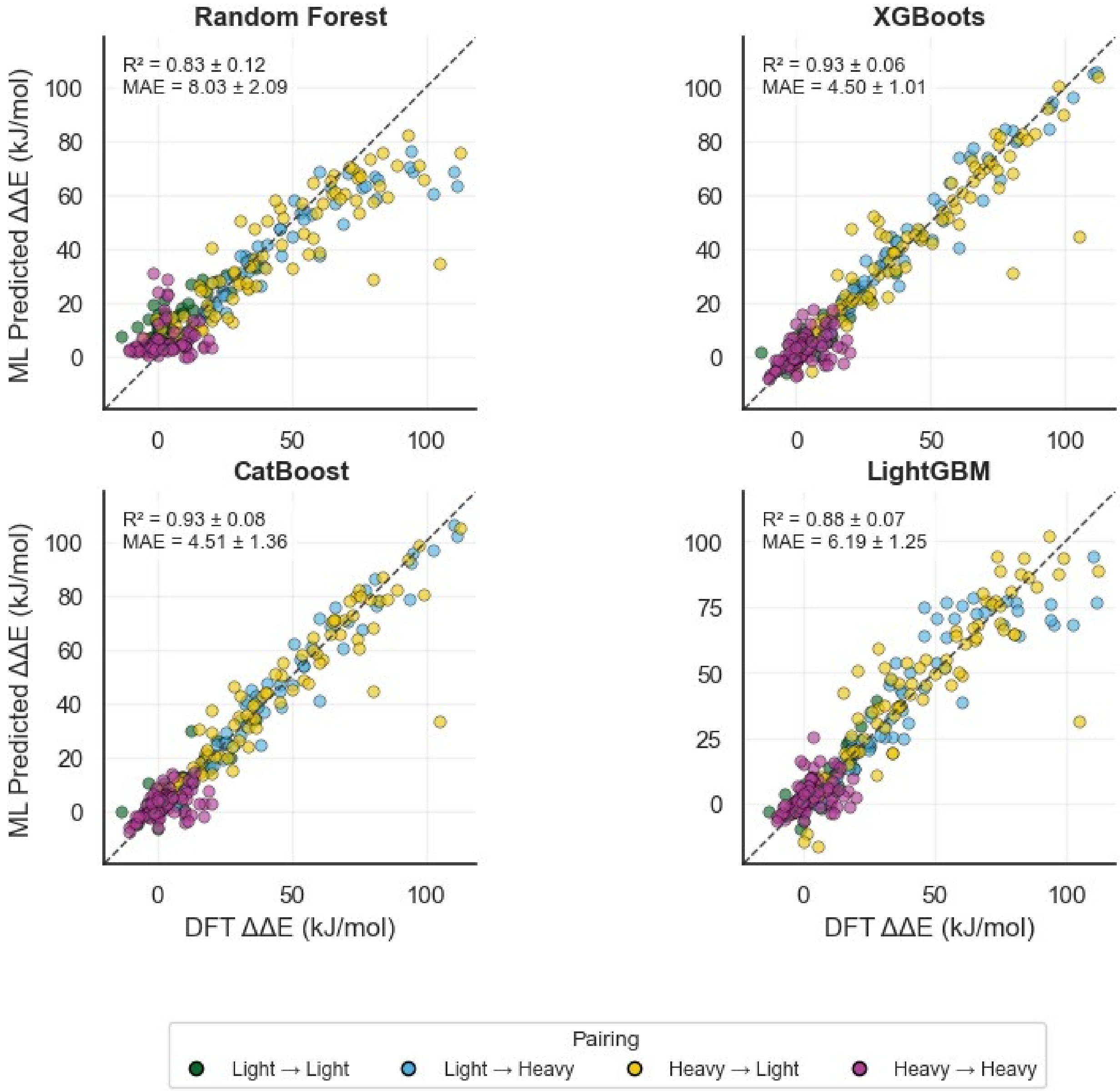

Figure S32: ΔΔE prediction by the four machine learning models vs DFT calculated ΔΔE under scenario (a), with *perfect template*.

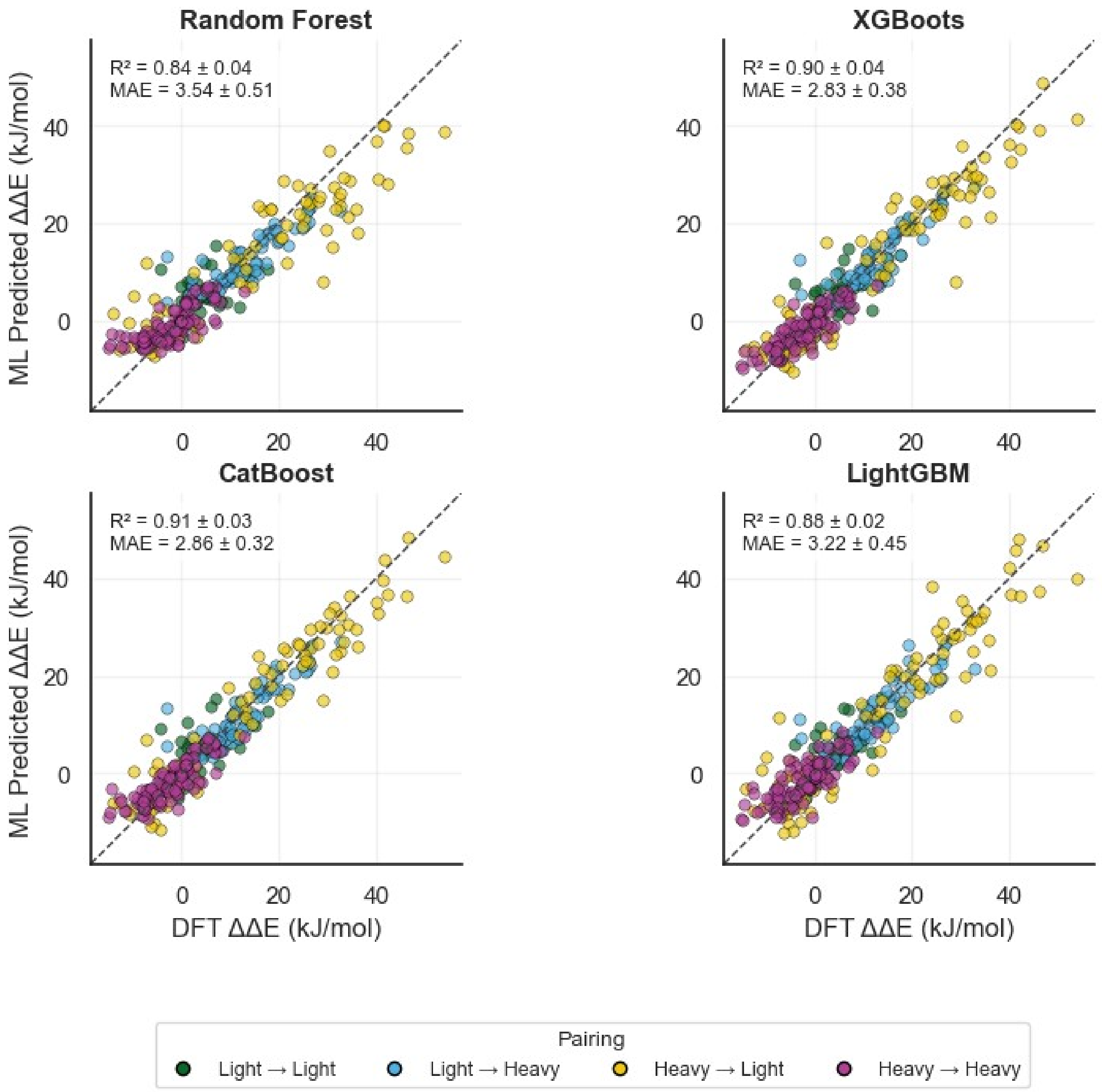

Figure S33: ΔΔE prediction by the four machine learning models vs DFT calculated ΔΔE under scenario (b), with *relaxed template*.

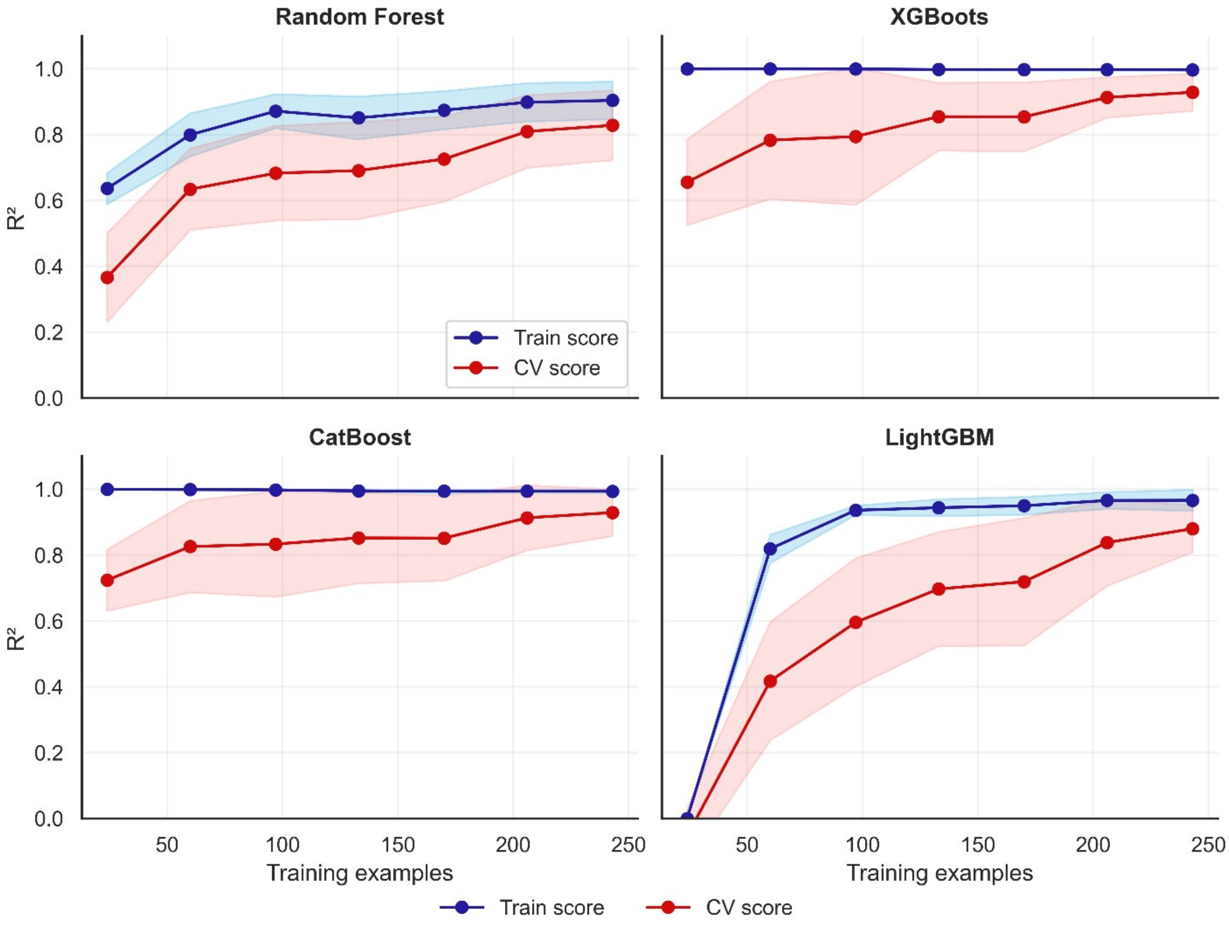

Figure S34: Learning curves of the four machine learning models used under scenario (a), with *perfect template*.

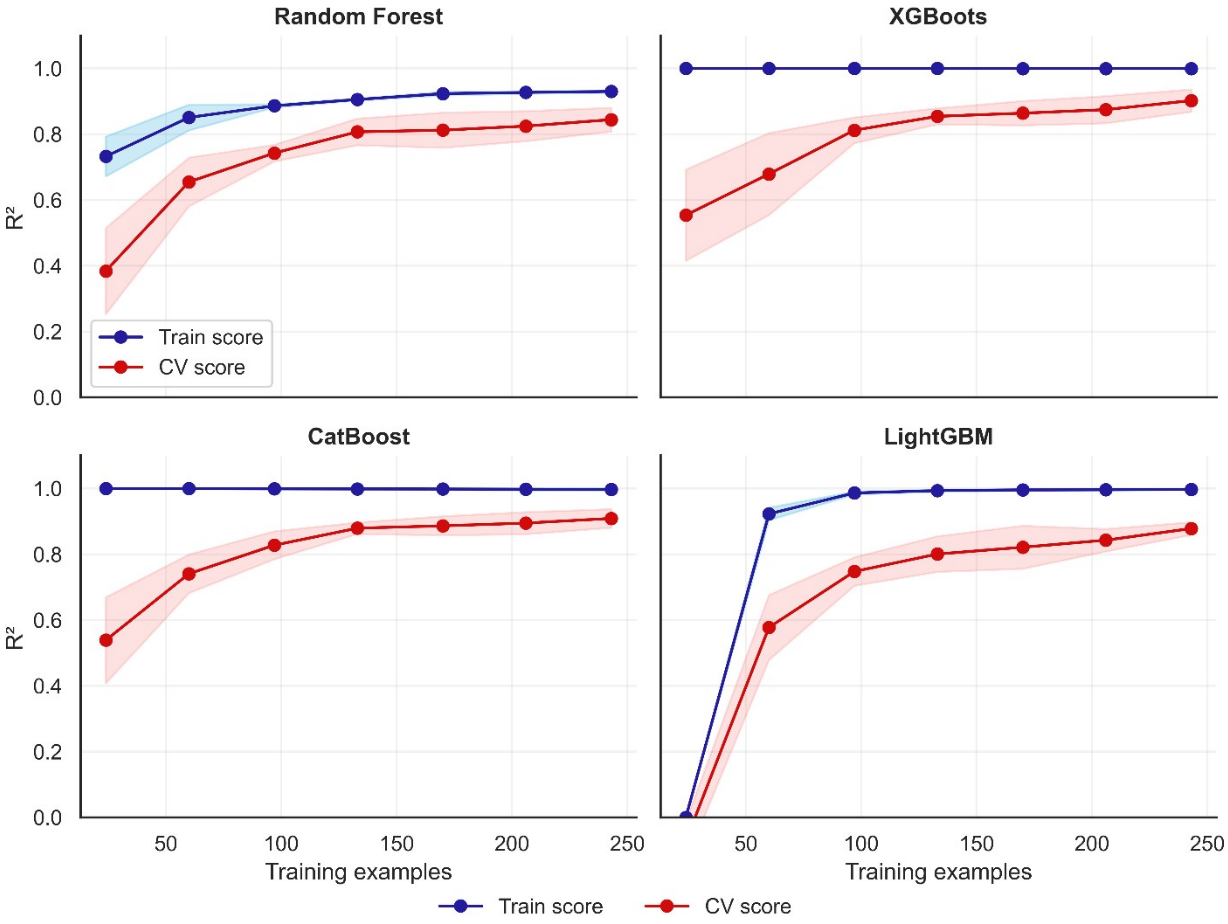

Figure S35: Learning curves of the four machine learning models used under scenario (b), with *relaxed template*.

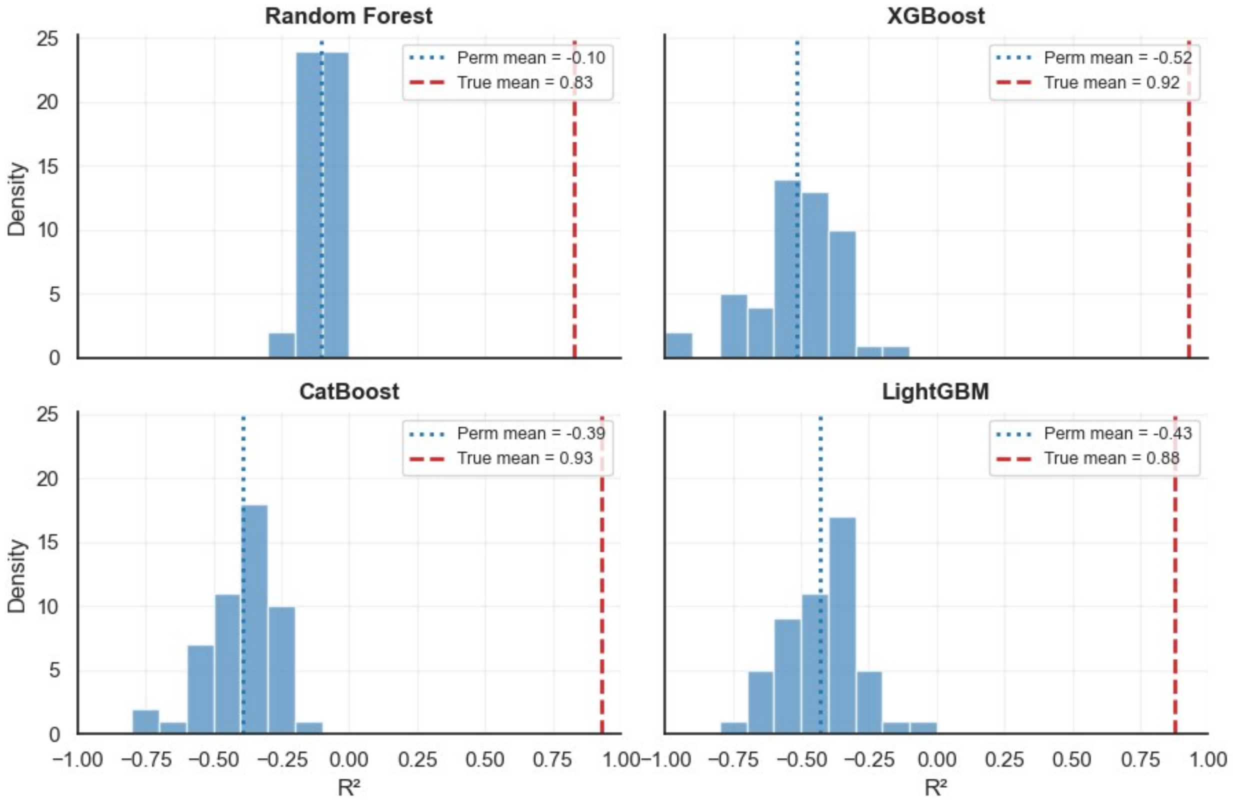

Figure S36: Y-Scramble test for the four machine learning models used under scenario (a), with *perfect template*.

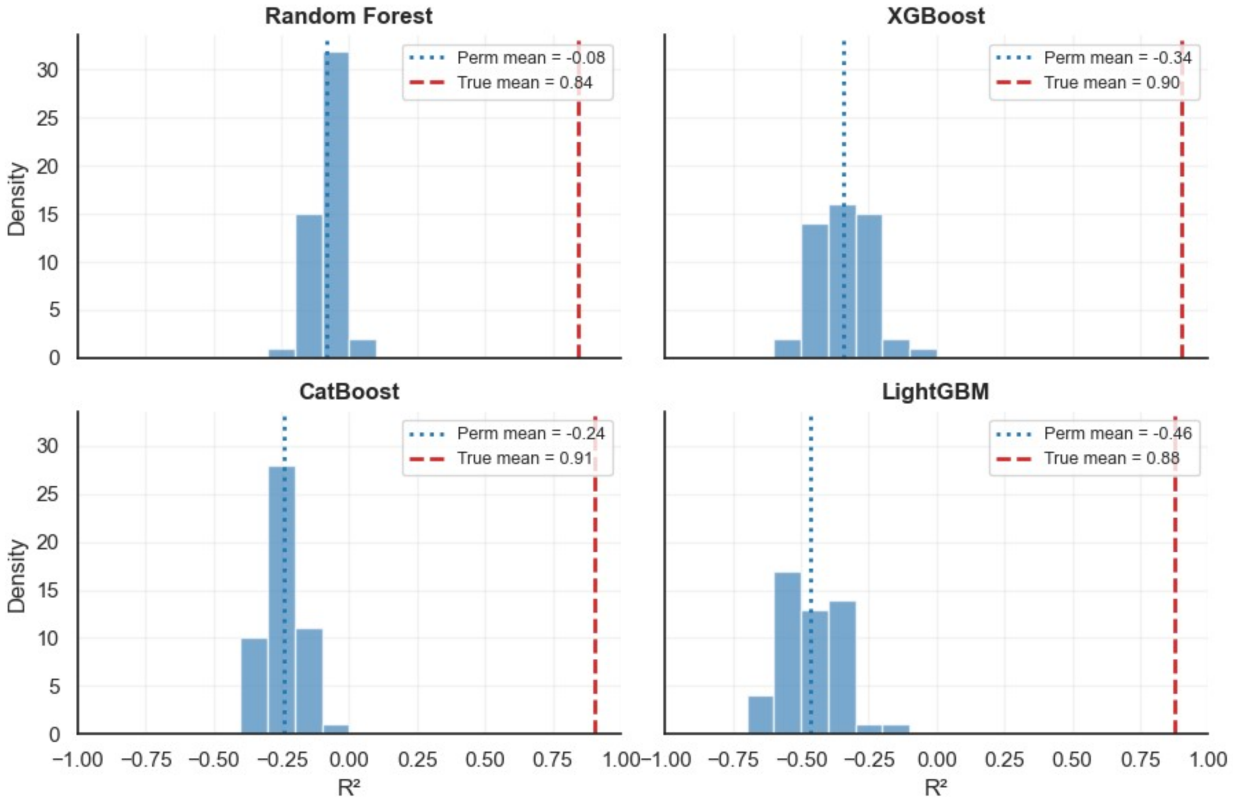

Figure S37: Y-Scramble test for the four machine learning models used under scenario (b), with *relaxed template*.

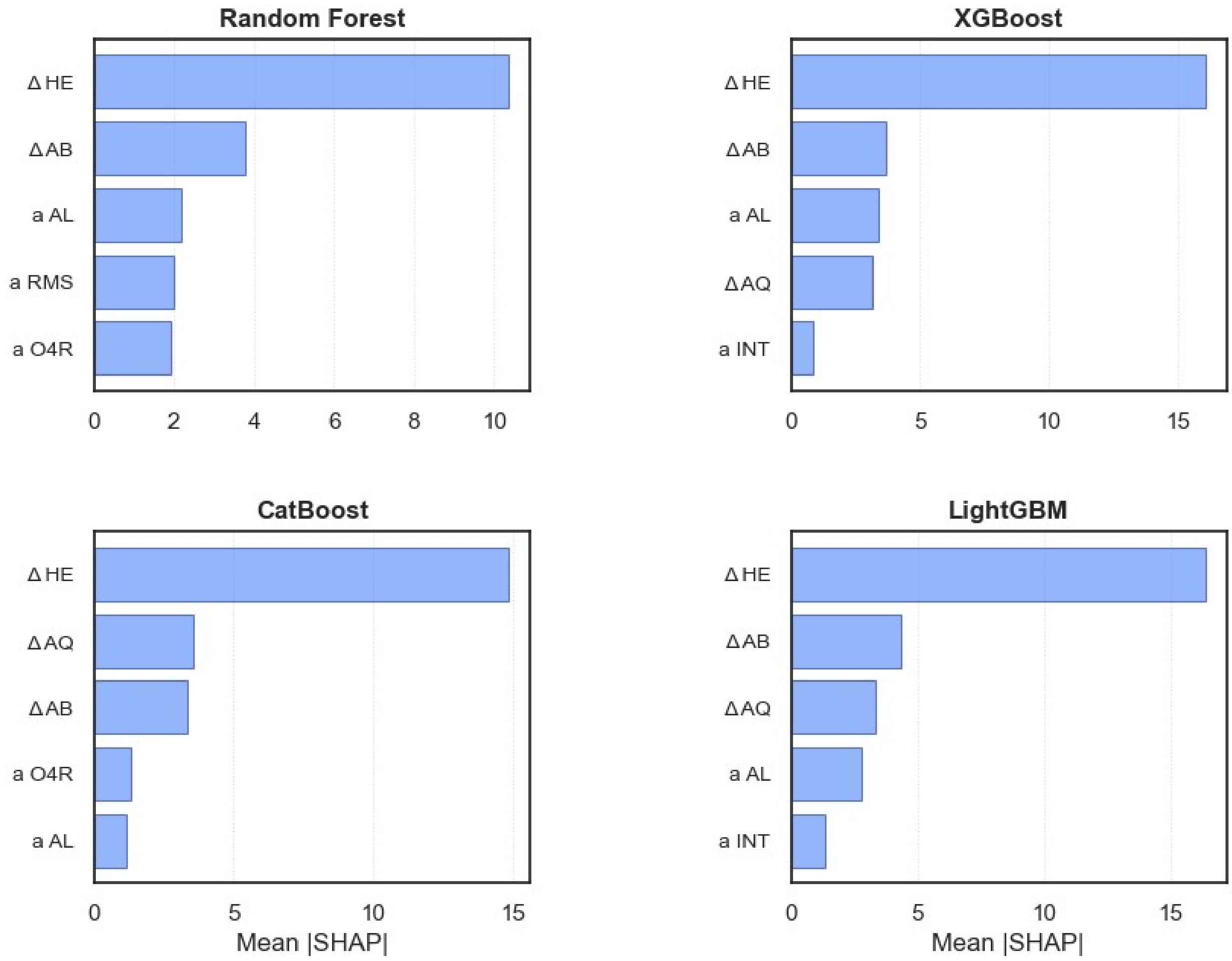

Figure S38: Mean |SHAP| value for the top 5 variables under scenario (a), with *perfect template*.

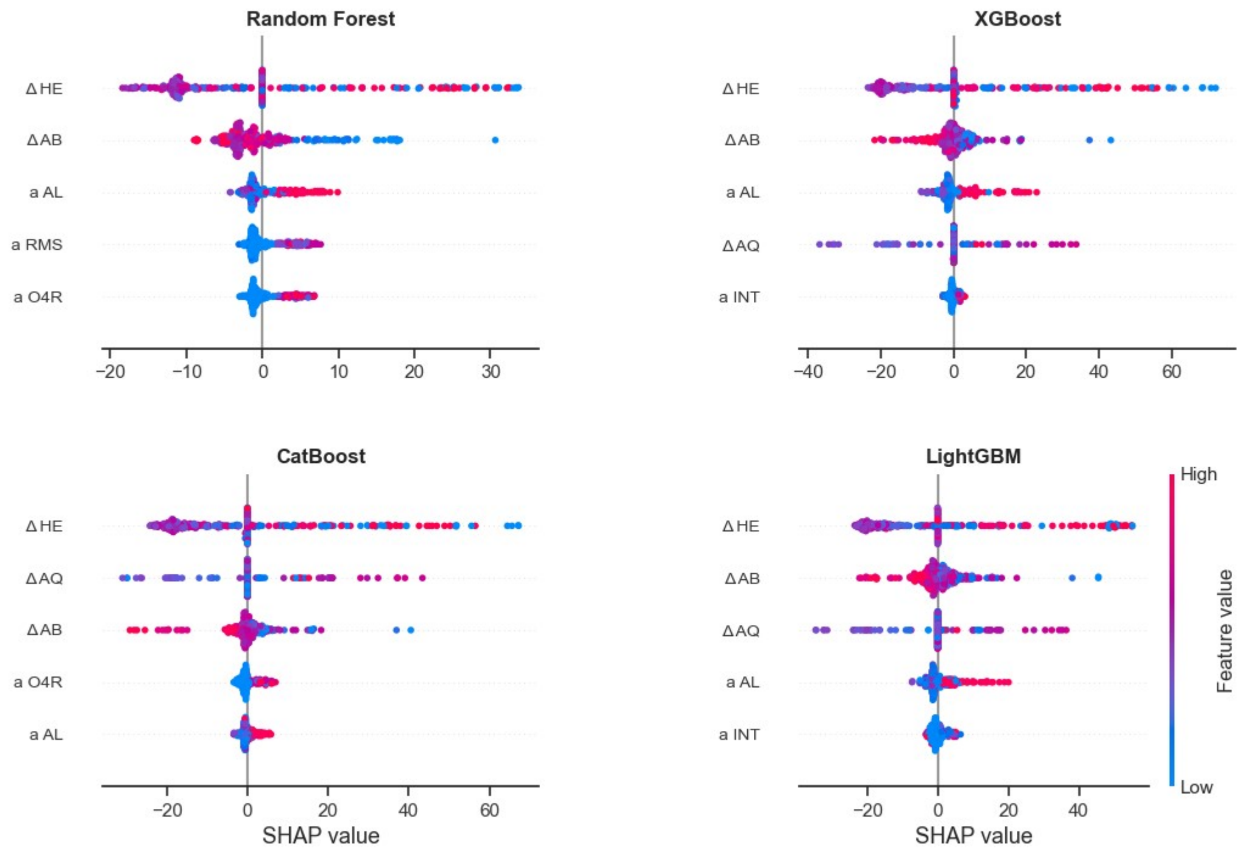

Figure S39: SHAP Beeswarm for the top 5 variables under scenario (a), with *perfect template.*

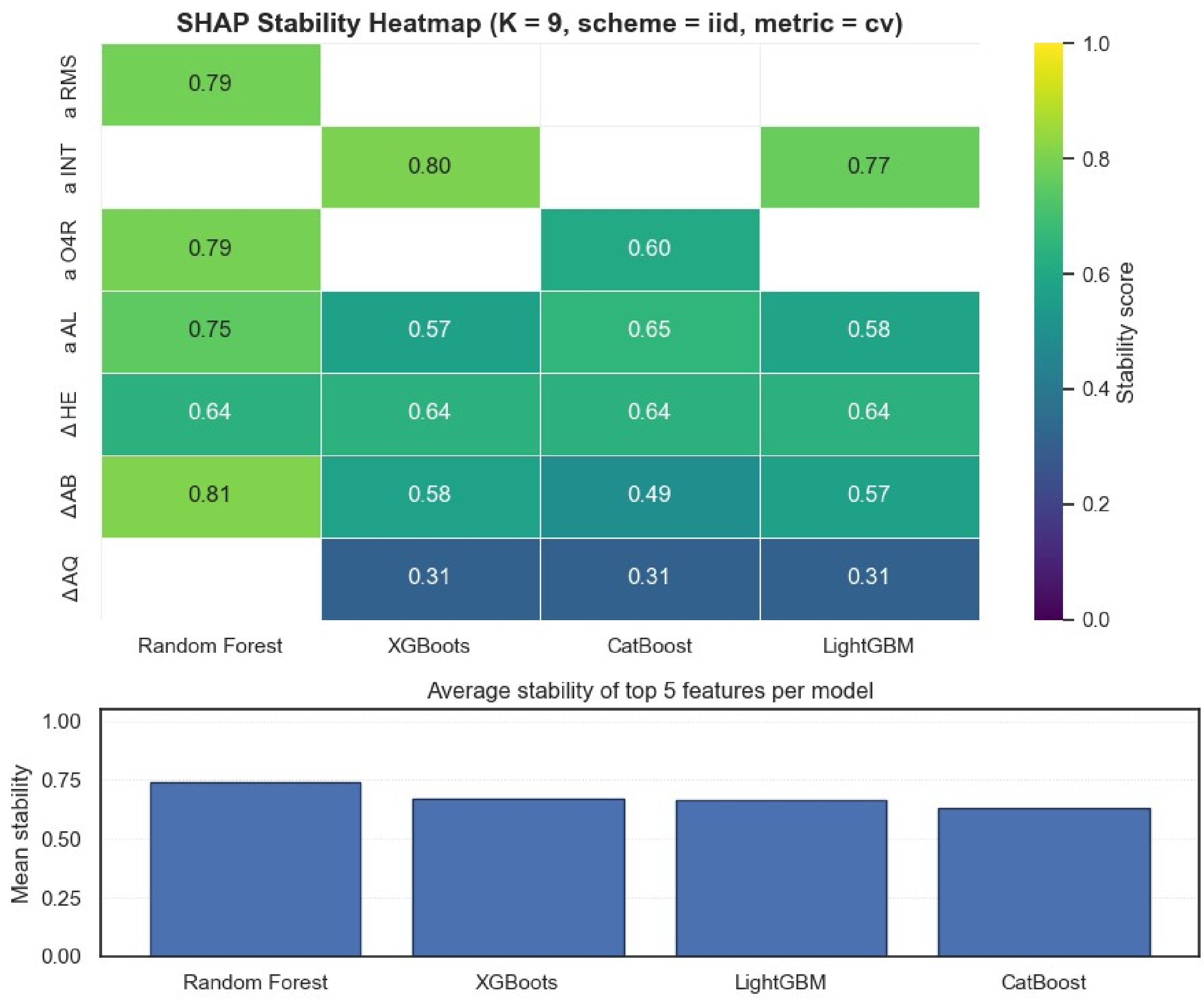

Figure S40: Stability of the SHAP top 5 feature across folds under scenario (a), with *perfect template*. The stability score was calculated by $s = {}^{1}/_{(1+cv)}$, $cv = \frac{SD|SHAP|}{mean|SHAP|}$

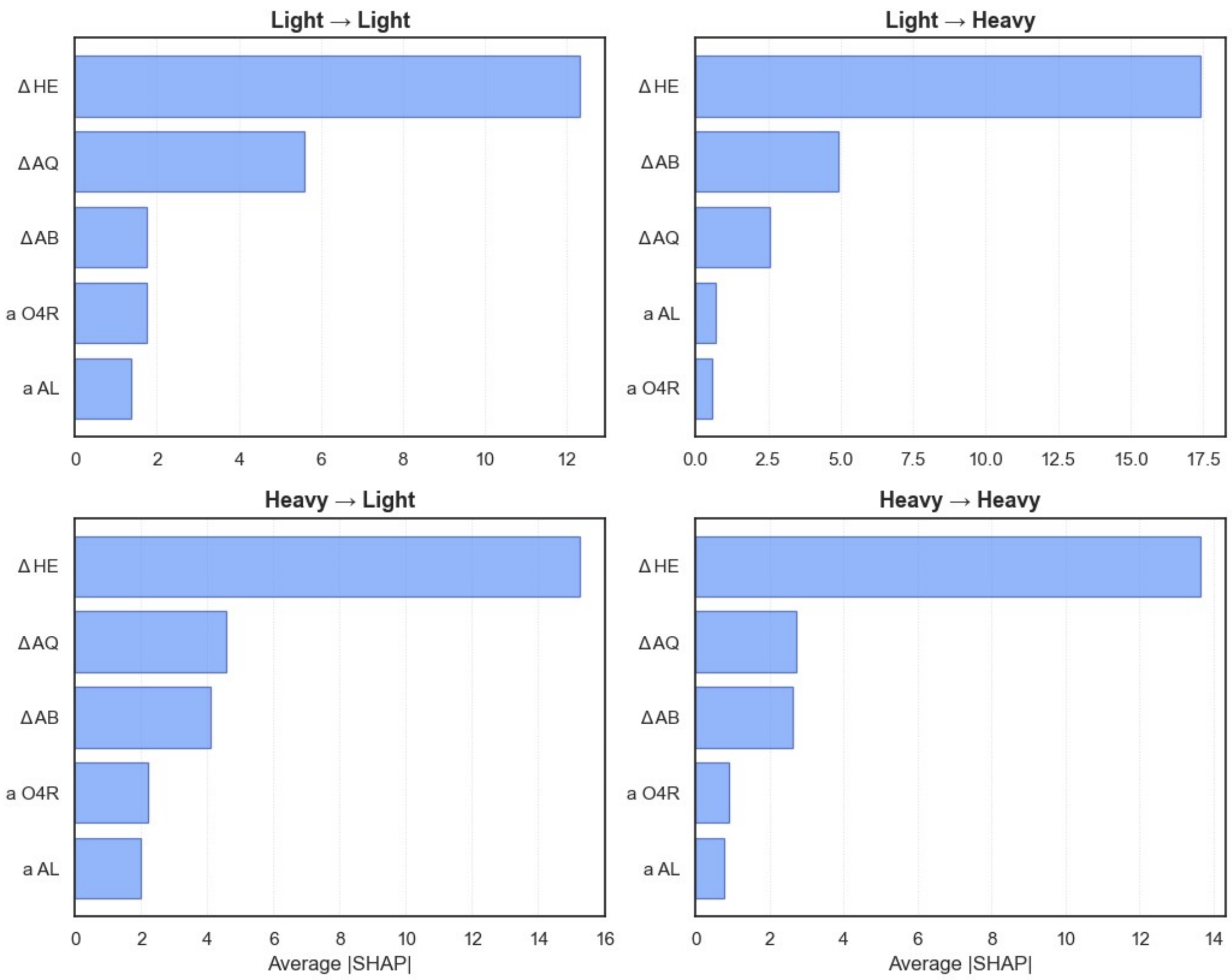

Figure S41: Mean |SHAP| value with CatBoost for the top 5 variables under scenario (a), with *perfect template*, subdivided into four substitution types (LRE→LRE, LRE→HRE, HRE→LRE, and HRE→HRE).

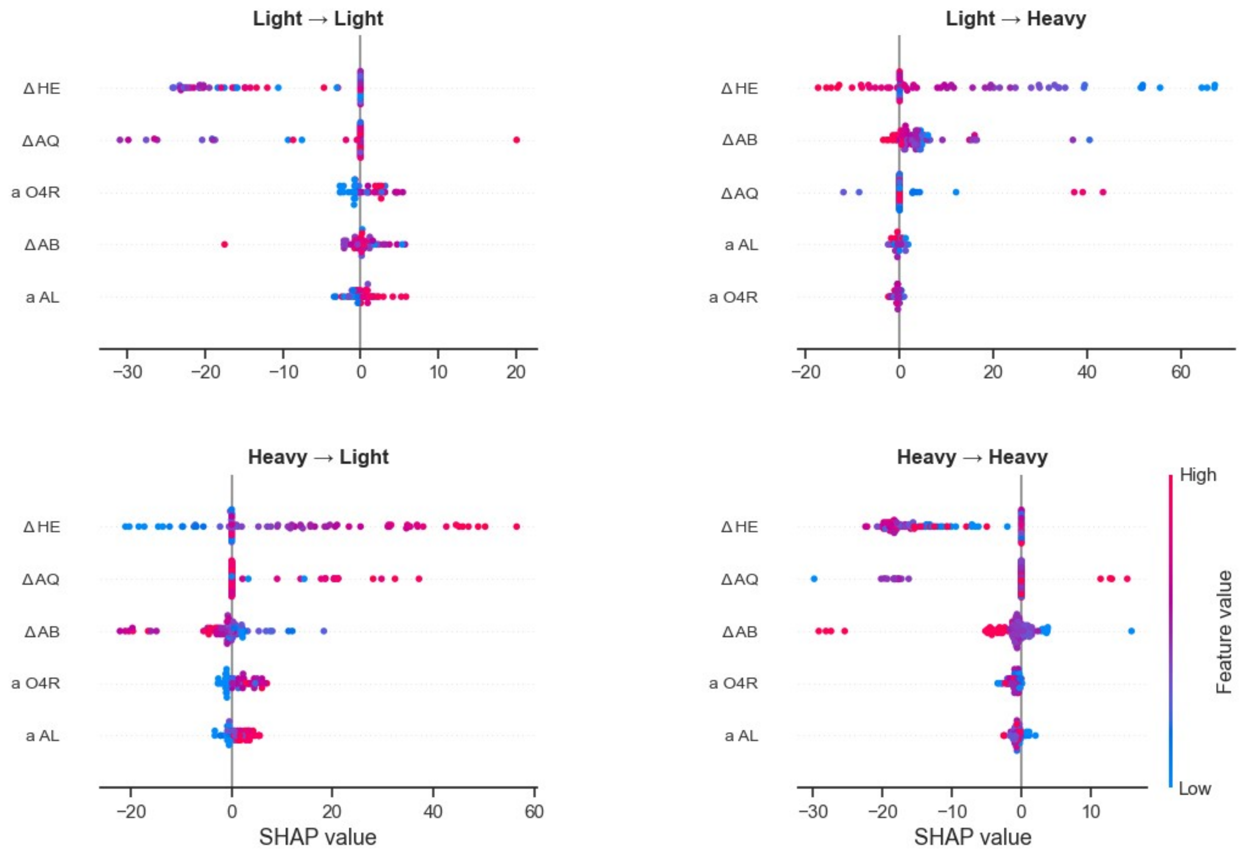

Figure S42: Beeswarm plots CatBoost for the top 5 variables under scenario (a), with *perfect template*, subdivided into four substitution types (LRE→LRE, LRE→HRE, HRE→LRE, and HRE→HRE).

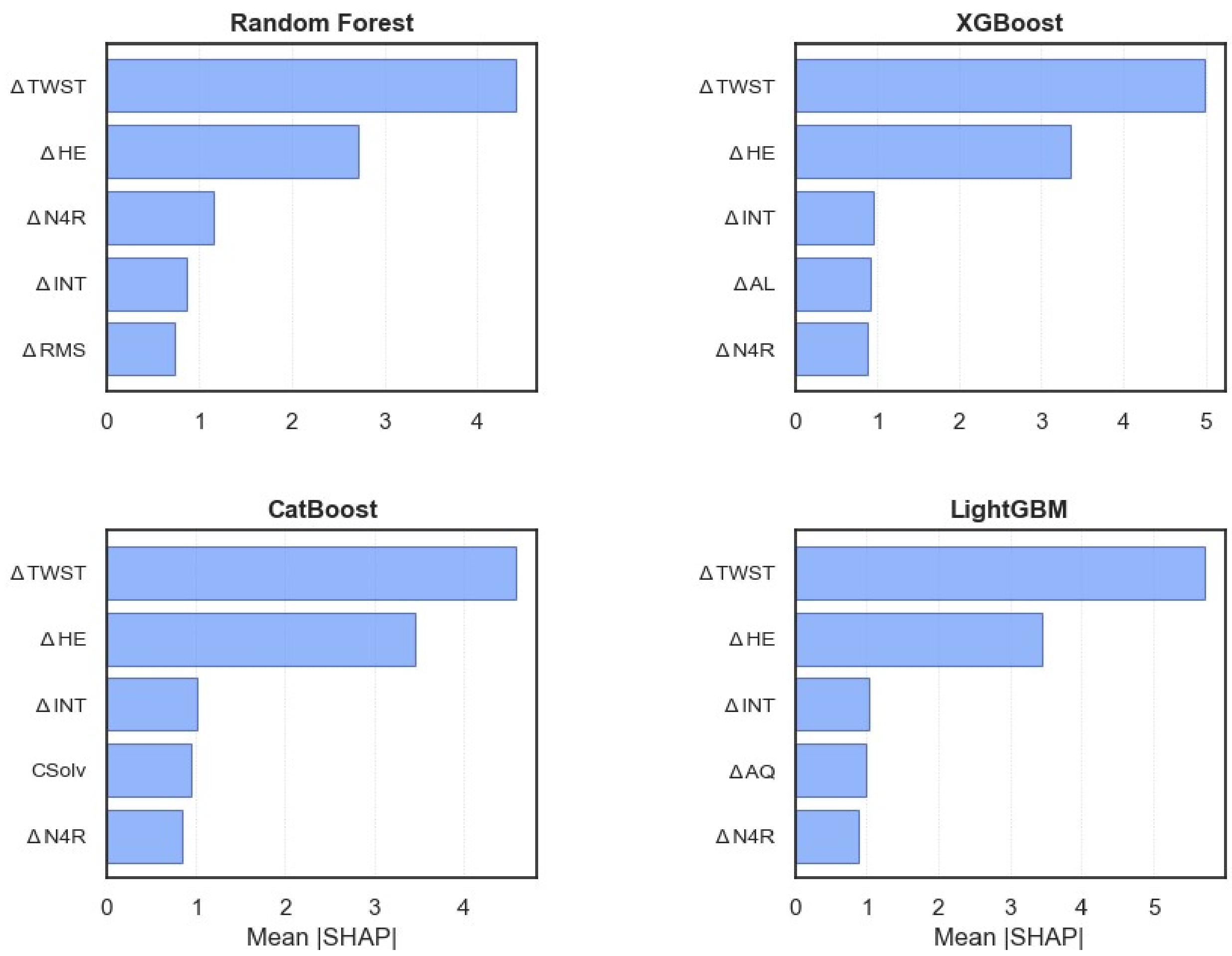

Figure S43: Mean |SHAP| value for the top 5 variables under scenario (b), with *relaxed template*.

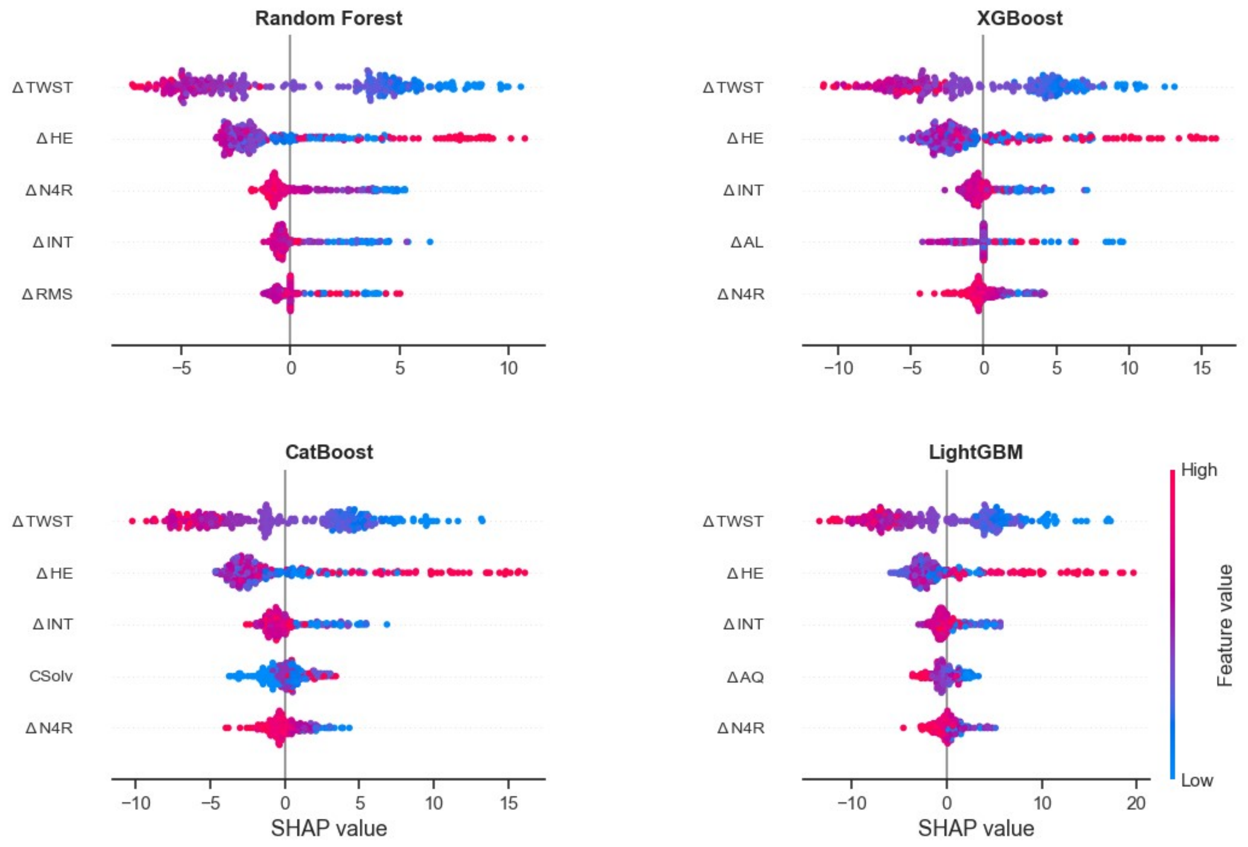

Figure S44: SHAP Beeswarm for the top 5 variables under scenario (b), with *relaxed template.*

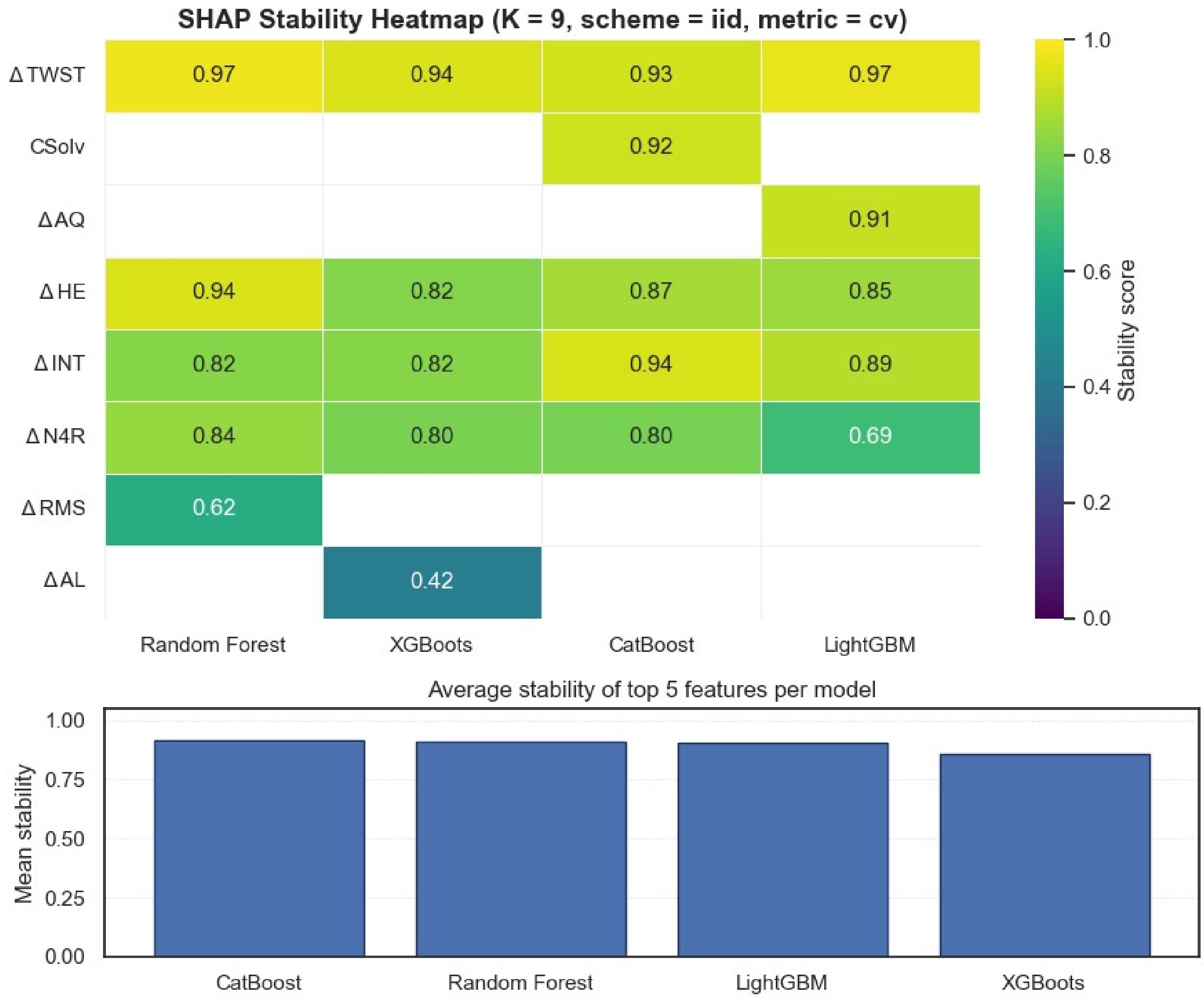

Figure S45: Stability of the SHAP top 5 feature across folds under scenario (b), *with relaxed template*. The stability score was calculated by $s = {}^{1}/_{(1+cv)}$, $cv = \dfrac{SD|SHAP|}{mean|SHAP|}$

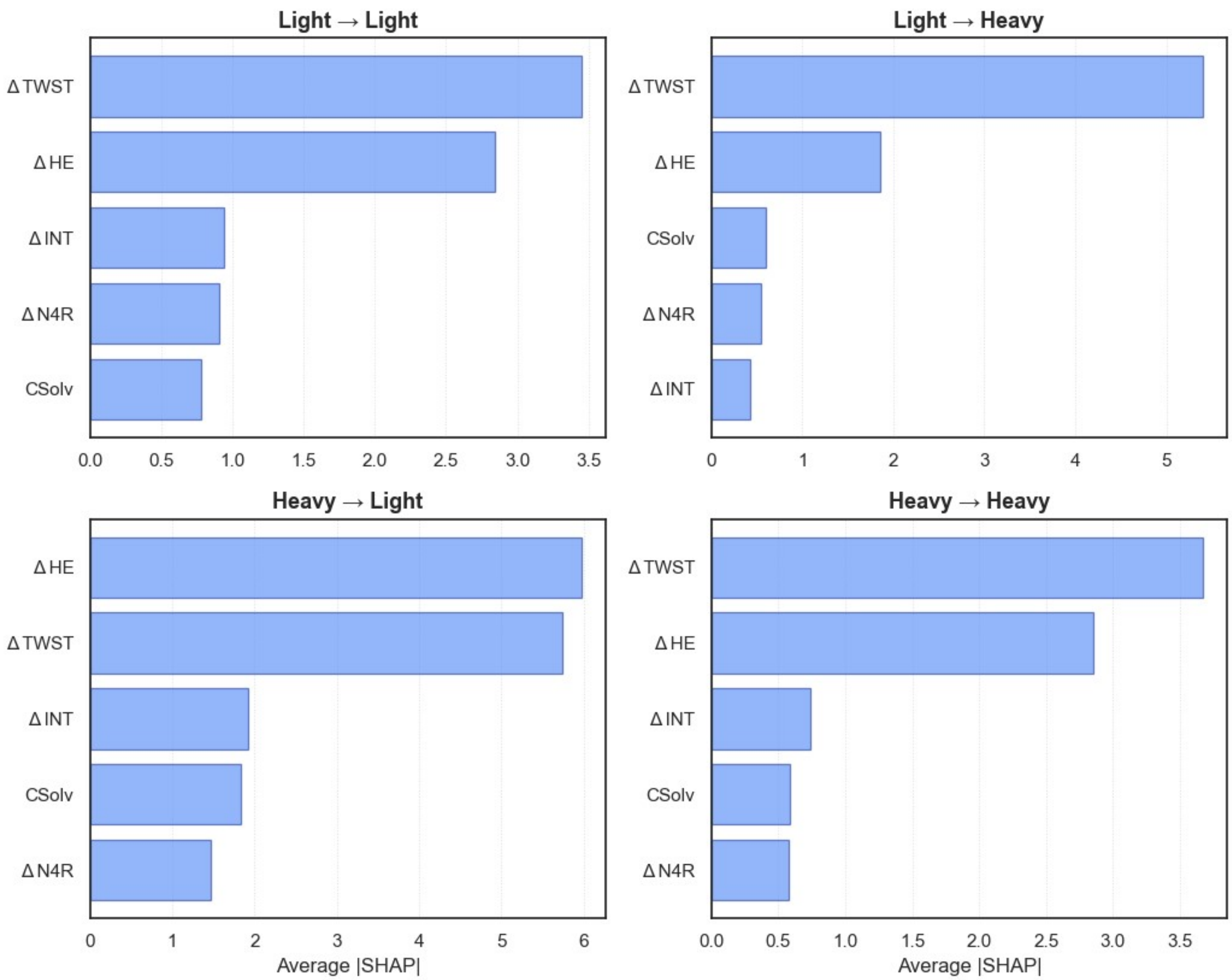

Figure S46: Mean |SHAP| value with CatBoost for the top 5 variables under scenario (b), with *relaxed template*, subdivided into four substitution types (LRE→LRE, LRE→HRE, HRE→LRE, and HRE→HRE).

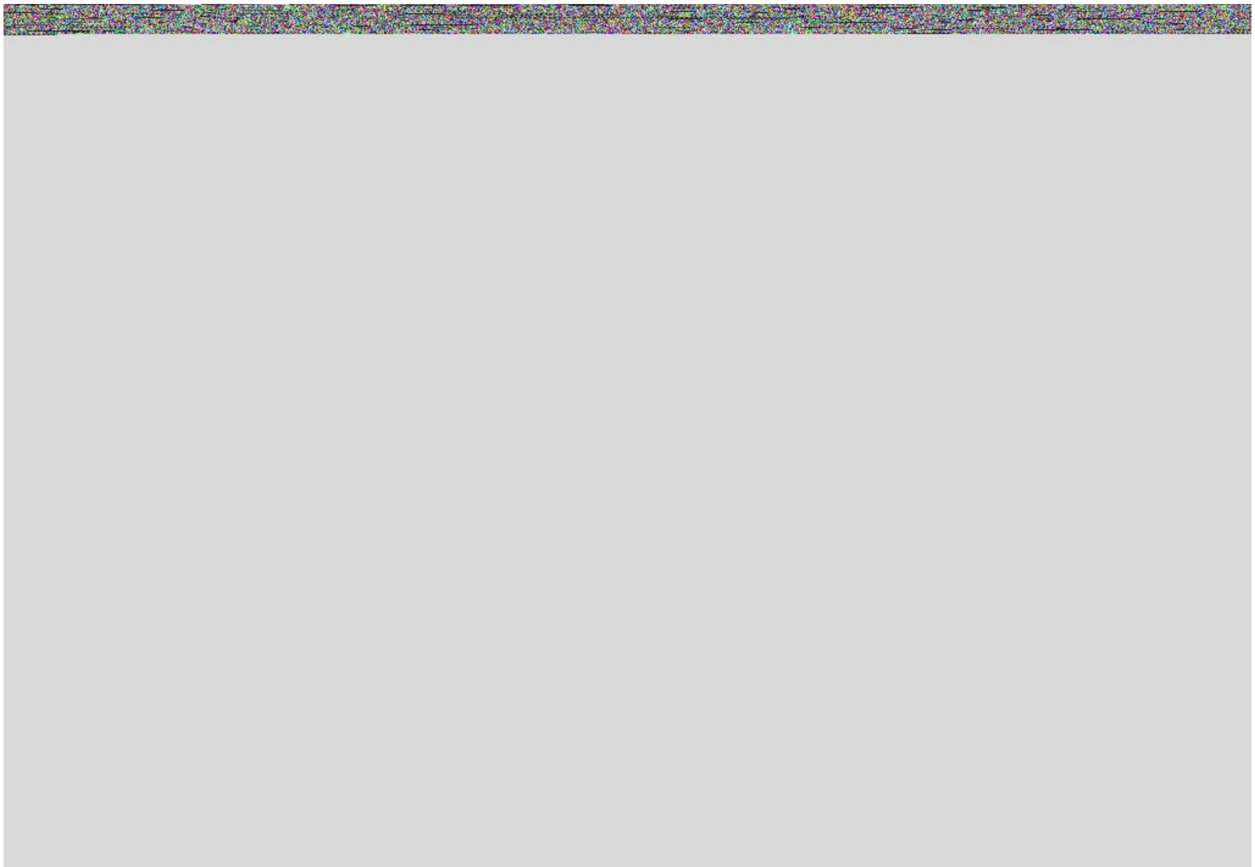

Figure S47: Beeswarm plots CatBoost for the top 5 variables under scenario (b), with *relaxed template*, subdivided into four substitution types (LRE→LRE, LRE→HRE, HRE→LRE, and HRE→HRE).
